# Tailored 3‐Alkoxy‐*N*,*N*,*N*,2,2‐Pentamethylpropan‐1‐Ammonium *Bis*(trifluoromethylsulfonyl)Imide Ionic Liquids for Room‐Temperature Fluoride‐Ion Batteries

**DOI:** 10.1002/anie.202422299

**Published:** 2025-04-14

**Authors:** Tiancheng Tan, Richard Murdey, Shunsuke Sumitomo, Kazuyuki Sato, Takeshi Abe, Atsushi Wakamiya

**Affiliations:** ^1^ Institute for Chemical Research Kyoto University Gokasho Uji Kyoto 611‐0011 Japan; ^2^ Office of Institutional Advancement and Communications Kyoto University Gokasho Uji Kyoto 611‐0011 Japan; ^3^ Graduate School of Engineering Kyoto University Nishikyo Kyoto 615–8510 Japan

**Keywords:** Electrolyte, Fluoride anion, Fluoride ion battery, Ionic liquid, NMR dynamic

## Abstract

Two novel liquid electrolytes for room‐temperature fluoride ion batteries are presented. These electrolytes are based on ionic liquids with quaternary ammonium cations 3‐methoxy‐*N*,*N*,*N*,2,2‐pentamethylpropan‐1‐aminium (MNPA) and *N*,*N*,*N*,2,2‐pentamethyl‐3‐(2,2,2‐trifluoroethoxy)propane‐1‐aminium (NPPA) combined with a *bis*(trifluoromethylsulfonyl)‐imide (TFSI) counterion. Quaternary ammonium fluorides can be added at concentrations up to 0.7 M to a functional fluoride electrolyte with a total diffusivity of 4.99 × 10^−12^ m^2^ s^−1^, a viscosity of 260 mP⋅s and a wide operational voltage range exceeding 5.4 V. The liquid ion electrolyte with the MNPA cation supports stable fluoride ion shuttling for up to 100 h.

## Graphical abstract

Two new quaternary ammonium TFSI ionic liquids, [MNPA][TFSI] and [NPPA][TFSI] were designed and synthesized. These ionic liquids readily dissolve quaternary ammonium fluorides (>0.7 M), and the resulting electrolytes have a wide operational voltage range (>5.4 V). Battery performance using Np_2_F/[MNPA][TFSI] demonstrated stable and reversible (de)fluorination reaction, with stable fluoride ion shuttling for 100 h.

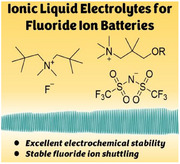

## Introduction

**Figure 1:**
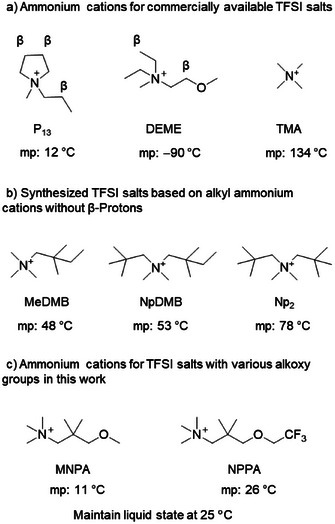
a) Some commercially available TFSI salts with quaternary ammonium cations, b) the synthesized TFSI salts based on alkyl ammonium cations without β‐protons, and c) the cations for ionic liquid in this work, MNPA, and NPPA.

Fluoride ion batteries (FIBs) are a promising class of energy storage devices distinguished by their high energy density.^[^ ^1^, ^2^, ^3^, ^4^, ^5^, ^6^, ^7^, ^8^, ^9^ ^]^ Since solid‐state electrolytes typically exhibit poor conductivity at low temperatures, recent attention has focused on high‐conductivity liquid electrolytes for FIBs.^[^ ^10^, ^11^, ^12^, ^13^, ^14^, ^15^, ^16^, ^17^, ^18^, ^19^, ^20^, ^21^, ^22^, ^23^, ^24^, ^25^, ^26^, ^27^, ^28^ ^]^ Solvating fluoride ions is challenging, however, due to several factors relating to solubility and chemical stability. For example, the high dissociation energy of the fluoride salts makes them hard to dissolve in aprotic solvents, resulting in poor conductivity. Additionally, the highly reactive fluoride salts can deprotonate solvents, leading to poor electrochemical stability resulting from the formation of HF₂⁻. Even the most successful aprotic solvent for ammonium fluorides, the fluorinated ether *bis*(2,2,2‐trifluoroethyl) ether (BTFE), is only able to dissolve a few quaternary ammonium fluoride salts, notably *N*,*N*‐dimethyl‐*N*,*N*‐dineopentylammonium fluoride (Np_2_F).^[^ ^15^, ^29^, ^30^ ^]^ The appeal of BTFE is further limited by its low boiling point and poor electrochemical stability.^[^ ^15^, ^29^ ^]^ Other candidate liquid‐state electrolytes are, therefore, highly desirable. Here, we note that ionic liquids combined with lithium salts have been successfully used as electrolytes for lithium‐ion batteries.^[^ ^31^, ^32^, ^33^, ^34^, ^35^, ^36^ ^]^ In particular, ionic liquids comprised of a quaternary ammonium cation and *bis*(trifluoromethylsulfonyl)imide (TFSI) anion are well known for their good electrochemical stability.^[^ ^37^, ^38^, ^39^, ^40^ ^]^ Therefore, it is reasonable to expect that quaternary ammonium TFSI ionic liquids mixed with fluoride salts might make suitable electrolytes for fluoride ion batteries. When applied to FIBs, however, ammonium cations with no β‐protons are needed as these protons would otherwise react with the fluoride ion through Hofmann elimination.^[^ ^41^ ^]^ As a result of this requirement, many cations used in commercial ionic liquids, such as 1‐methyl‐1‐propylpyrrolidinium (P_13_),^[^ ^42^ ^]^ and *N*,*N*‐diethyl‐*N*‐methyl‐*N*‐(2‐methoxyethyl)ammonium (DEME, Figure 1a)^[^ ^43^ ^]^ will be unsuitable for FIBs.

This work presents the design and synthesis of new TFSI salts utilizing ammonium cations without β‐protons. TFSI salts with a liquid state at room temperature were achieved by introducing alkoxy groups to the ammonium cations. These ionic liquids demonstrate good solubility and stability toward Np_2_F fluoride salt. Both the Np_2_F/[MNPA][TFSI] and Np_2_F/[NPPA][TFSI] electrolytes also have wide operational voltage ranges exceeding 5.4 V. The wide voltage window of the electrolytes supports the use of cathodes with high working potentials, such as Ag. Np_2_F/[MNPA][TFSI] supports stable fluoride anion shuttling for up to 100 h. These favorable results highlight the promising possibilities of electrolytes based on ionic liquids for FIBs operating at room temperature.

## Results and Discussion

### Design and Synthesis

Although tetramethylammonium (TMA, Figure 1a) TFSI has no β‐protons, the high melting point of 134 °C makes it unsuitable for room‐temperature electrolytes.^[^ ^44^ ^]^ We found that the melting point could be reduced to 48 °C by substituting a flexible 2,2‐dimethylbutyl group for one of the methyl groups in the TMA backbone (MeDMB, Figure 1b). Substituting two neopentyl groups (Np_2_), or one neopentyl group and one 2,2‐dimethylbutyl group (NpDMB), resulted in melting points of 78 and 53 °C, respectively (Figure 1b). These results confirm that flexible substituents on the quaternary ammonium cations are crucial to keeping the TFSI salt in a liquid state at room temperature. Indeed, alkoxy functional groups have long been used to synthesize low melting point, low viscosity, and high conductivity ionic liquids.^[^ ^45^, ^46^, ^47^, ^48^, ^49^ ^]^ Therefore, we substituted a 1‐alkoxy‐2,2‐dimethylpropyl group into the tetramethylammonium (TMA) to lower the melting point to near room temperature. Two variants were synthesized: One with a simple methoxy group, which gave 3‐methoxy*‐N,N,N,2,2‐*pentamethylpropan‐1‐aminium (MNPA), and the other with a trifluoroethoxy group (CF_3_CH_2_O‐), giving *N,N,N*,2,2‐pentamethyl‐3‐(2,2,2‐trifluoroethoxy)propan‐1‐aminium (NPPA). As anticipated, [MNPA][TFSI] and [NPPA][TFSI] are both liquids at room temperature (Figure 1c).

We chose the NPPA cation expecting that the highly acidic protons on the trifluoroethoxy group would enhance the solubility of quaternary ammonium fluoride salts through hydrogen bonding.

**Scheme 1:**
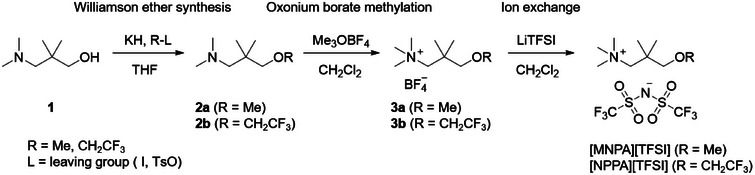
Synthetic route to the ionic liquids used in this study.

The alkoxy‐substituted quaternary ammonium TFSI salts were synthesized according to Scheme 1. To prepare the amino ether intermediates (**2a**, **2b**), the commercially available starting material, 3‐(dimethylamino)‐2,2‐dimethylpropan‐1‐ol (**1**), was first deprotonated with potassium hydride (KH). Electrophiles (R‐L) were added to substitute the terminal group to yield the amino ether intermediates. The products of the Williamson ether synthesis were then methylated through oxonium borate methylation using Meerwein's salt (trimethyloxonium tetrafluoroborate) in dichloromethane, giving the ammonium borate salts (**3a**, **3b**). The solutions of TFSI salt were formed by mixing the borate salts with LiTFSI in dichloromethane. After removing LiBF_4_ and excess LiTFSI by extraction, the solutions were dried under vacuum at 80 °C to yield the TFSI salts ([MNPA][TFSI] and [NPPA][TFSI]) as colorless liquids. Additional details of the synthesis are provided in the Supporting Information. Purity was confirmed by ^1^H, ^13^C, and ^19^F NMR in deuterated organic solvents, and low‐temperature solid‐state structures were obtained by single‐crystal X‐ray diffraction analysis.

This synthetic route has merit at both laboratory and industrial scales, as 1) no toxic chemicals are used, 2) no special handling considerations are required, 3) all reagents are commercially available, 4) reactions proceed rapidly, and, 5), no complex or expensive purification methods are necessary for product isolation.

### Thermodynamic Properties

**Figure 2:**
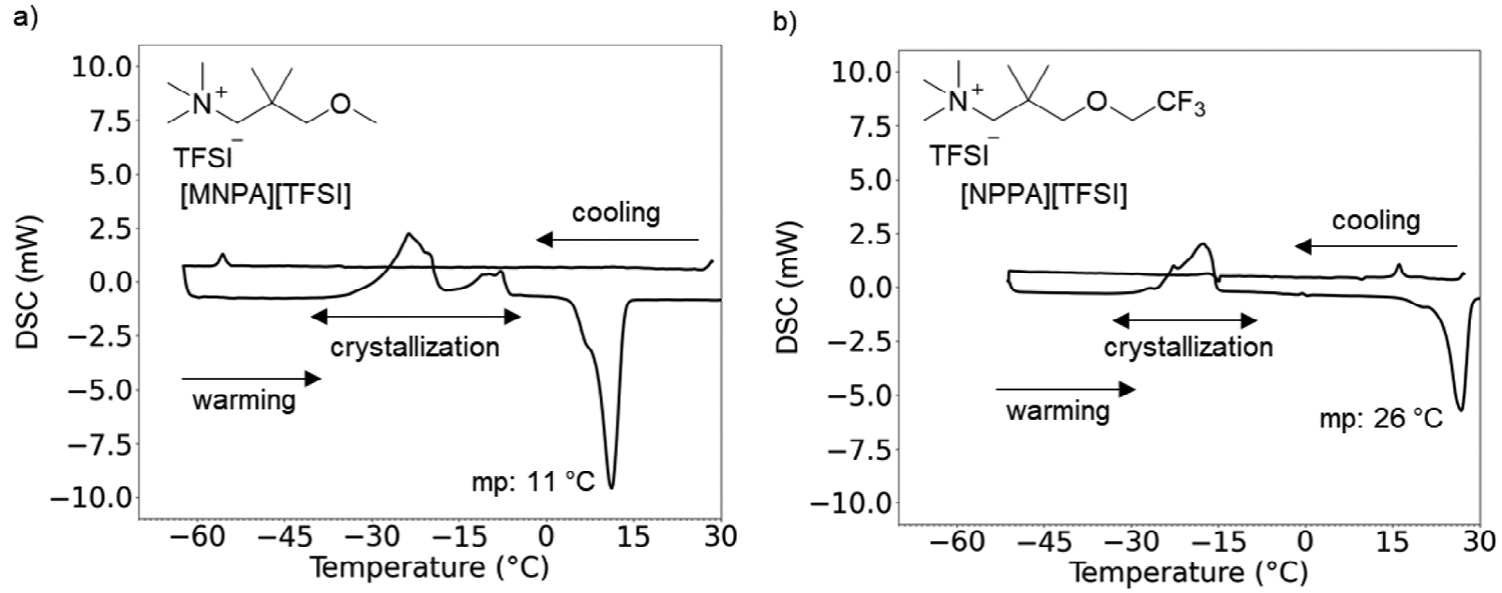
DSC trace of a) [MNPA][TFSI] and b) [NPPA][TFSI] with 3 °C min^−1^ scan rate.

The phase transitions of [MNPA][TFSI] and [NPPA][TFSI] were investigated using differential scanning calorimetry (DSC). Although no clear indication of crystallization was observed when cooling the sample to −50 °C, crystallization was observed from −30 to −5 °C on warming (Figure 2). Once solidified, the melting point of [MNPA][TFSI] and [NPPA][TFSI] could be estimated at 11 and 26 °C, respectively. These phase transitions did not significantly change under multiple temperature cycles (Figure S47). Both [MNPA][TFSI] and [NPPA][TFSI] remain in a liquid state at room temperature (25 °C) with behavior reminiscent of supercooled liquids. From thermogravimetric analysis, thermal stability is assessed to be relatively high as the mass loss at 292 °C was less than 1% for both [MNPA][TFSI] and [NPPA][TFSI] (Figure S1).

### Single Crystal X‐Ray Diffraction Analysis

The crystal structure of [MNPA][TFSI] and [NPPA][TFSI] provides insight into ion‐ion arrangements and interactions in both the solid and liquid states.^[^ ^50^, ^51^, ^52^, ^53^, ^54^, ^55^ ^]^ Guided by the DSC results, colorless plate‐like single crystals were prepared by slowly warming the ionic liquids from −70 °C to their crystallization temperature. Samples were stored below 0 °C and measured at −173 °C. The detailed methods are given in the Supporting Information.

**Figure 3:**
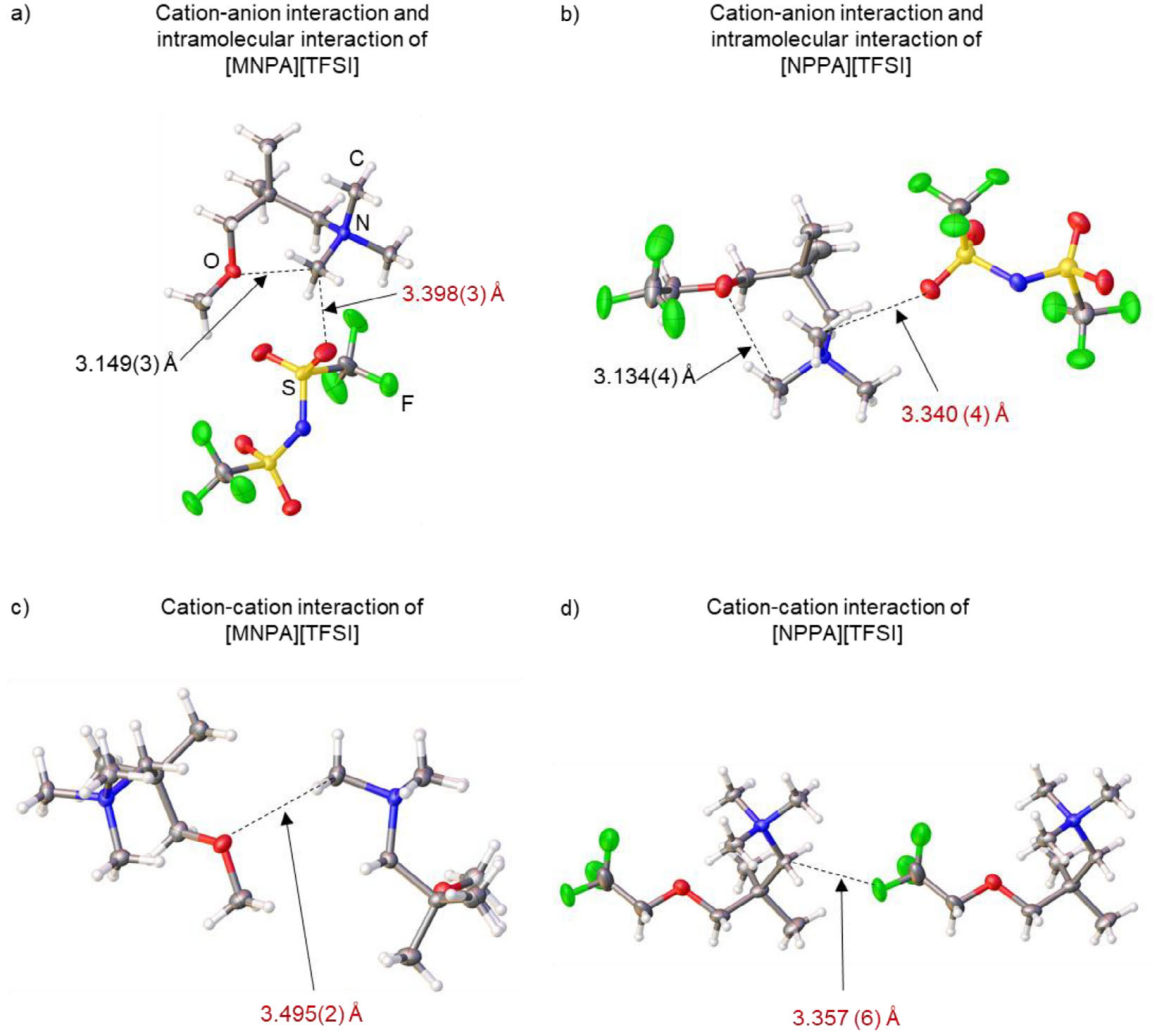
Crystal structures of [MNPA][TFSI] (a,c) and [NPPA][TFSI] (b,d) with displacement ellipsoids drawn at 50% probability. The dotted lines indicate the axis of the principal intermolecular and intramolecular interactions discussed in the text.

As shown in Figure 3a,b, the ammonium cations in both [MNPA][TFSI] and [NPPA][TFSI] have intermolecular interactions through C─H⋯O hydrogen bonds, with C⋯O distances of 3.149(3) and 3.134(4) Å, respectively. The TFSI anions are in the *trans* conformation with a C─S⋯S─C angle of 180° for both compounds, as previously reported for other TFSI‐based ionic liquids.^[^ ^51^, ^52^ ^]^ Cation–anion interactions through C─H⋯O, C─H⋯N, and C─H⋯F hydrogen bonds are observed, as well as a cation–cation interaction through the C─H⋯O hydrogen bond for [MNPA][TFSI] and the C─H⋯F hydrogen bond for [NPPA][TFSI]. For [MNPA][TFSI], the cation–anion interaction through C─H⋯O hydrogen bonds has a minimum C⋯O distance of 3.398(3) Å. In [NPPA][TFSI], the corresponding C⋯O distance is shorter, at 3.340(4) Å.

The cation–cation interaction for [MNPA][TFSI] (Figure 3c) forms between the oxygen of the methoxy group and the α‐proton of the methyl group. The C⋯O distance was determined to be 3.495(2) Å. Meanwhile, for [NPPA][TFSI] (Figure 3d), the cation–cation interaction forms between the fluorine on the trifluoroethoxy group and the α proton of the ammonium with a C⋯F distance of 3.357(6) Å. The shorter interaction distances in [NPPA][TFSI] compared with [MNPA][TFSI] arise from the trifluoroethoxy group present in the NPPA cation.

To determine the energy associated with these ion‐ion interactions, theoretical calculations were performed using the DLPNO‐CCSD(T) method with the def2‐TZVPP basis set. The initial geometries were extracted from the single crystal data. In the case of [NPPA][TFSI], the cation–anion attraction was calculated to be −274.0 kJ mol^−1^, while the cation–cation repulsion was 100.2 kJ mol^−1^. In contrast, [MNPA][TFSI] exhibited a lower cation–anion attraction of −253.2 kJ mol^−1^ and a higher cation–cation repulsion of 122.2 kJ mol^−1^. Although no significant differences were observed in the partial charge of the α‐proton on the MNPA and NPPA cations (Figure S2), the strongly electron‐withdrawing trifluoroethoxy group is thought to enhance ion‐ion attraction by providing additional points of interaction rather than affecting the partial charge on the α‐proton of the ammonium cations through inductive effect. Specifically, the trifluoroethoxy group introduces an extra hydrogen bond donor (the proton on the trifluoroethoxy group) and an additional hydrogen bond acceptor (the fluorine atoms on the trifluoroethoxy group). These interactions lead to stronger cation–anion attraction and reduced cation–cation repulsion in [NPPA][TFSI], resulting in a slightly higher melting point of 26 °C for [NPPA][TFSI], compared to 11 °C for [MNPA][TFSI].

### Solubility, Stability, and Conductivity

The solubility of the ammonium fluoride salt Np_2_F was confirmed to be 0.8 M in [MNPA][TFSI] and 1.1 M in [NPPA][TFSI]. For comparison, the solubility in the commercial ionic liquid [P_13_][TFSI] is only 0.3 M. This highlights the critical contribution of the alkoxy group in [MNPA][TFSI] and [NPPA][TFSI] to the solubility of ammonium fluoride salts.

The chemical stability of the ionic liquids toward Hofmann elimination was studied using ^1^H NMR. After dissolving Np_2_F, the resulting electrolytes Np_2_F/[MNPA][TFSI] and Np_2_F/[NPPA][TFSI] showed good stability toward fluoride at room temperature (Figures S4, S5). For comparison, [DEME][TFSI], which has β‐protons on the ammonium cation, underwent decomposition via Hofmann elimination, resulting in the formation of tertiary amine (Figure S3). This observation confirms how ammonium cations without β‐protons are crucial for maintaining the chemical stability of the electrolytes.

Another concern with liquid fluoride electrolytes is the creation of bifluoride anions. Trace amounts of bifluoride anion were observed in the ^1^H and ^19^F NMR data (Figures S37–S40). However, the corresponding signal from the fluoride anion is obscured by the overlapping signal from TFSI. Unreacted fluoride anions in the electrolytes can be confirmed by sampling the electrolyte in deuterated solvent DMSO‐*d*_6_ as any fluoride anions will deprotonate the solvent to form DF_2_^–^. The ^19^F NMR spectrum (Figures S41, S42) confirms the presence of F^–^ and DF_2_^–^. In contrast, only a trace amount of HF_2_^–^ was observed. Meanwhile, no decomposition of the Np_2_F/[MNPA][TFSI] and Np_2_F/[NPPA][TFSI] electrolytes were detected by ^13^C NMR (Figures S43, S44). These results show that HF_2_^–^ exists in trace amounts that are not expected to influence the stability of the electrolyte system.

From electrochemical impedance measurements, the conductivity at 25 °C of both 0.5 M Np_2_F/[MNPA][TFSI] and 0.5 M Np_2_F/[NPPA][TFSI] was estimated to be 0.8 mS cm^−1^. The conductivity of pure ionic liquids is slightly higher, 1.9 mS cm^−1^ for [MNPA][TFSI] and 1.3 mS cm^−1^ for [NPPA][TFSI].

### Electrochemical Stability

**Figure 4:**
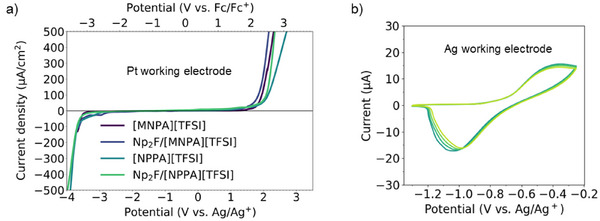
a) Voltage windows of [MNPA][TFSI], 0.1 M Np_2_F/[MNPA][TFSI], [NPPA][TFSI], and 0.1 M Np_2_F/[NPPA][TFSI] with a scan rate of 1 mV s^−1^. b) Cyclic voltammograms of the Ag electrode in 0.1 M Np_2_F/[MNPA][TFSI] with a scan rate of 1 mV s^−1^.

Turning to the electrochemical stability, the voltage window of the ionic liquids and the corresponding electrolytes was evaluated by linear sweep voltammetry. The voltages are referenced to a double junction electrode based on Ag/silver trifluoromethanesulfonate (AgOTf) in [P_13_][FSI] (0.38 V vs. Fc/Fc^+^; see Supporting Information). The voltage limits are defined by an arbitrary cutoff, *J*_cutoff_, of 100 µA per cm^2^. The neat ionic liquid, [MNPA][TFSI], showed broad stability from −3.6 V (vs. Ag/Ag^+^) to 2.0 V (vs. Ag/Ag^+^) for a voltage window of 5.6 V (Figure 4a). The oxidative stability of [NPPA][TFSI] was slightly higher at 2.2 V (vs. Ag/Ag^+^), while the reductive stability was unchanged at −3.6 V (vs. Ag/Ag^+^) for a voltage window of 5.8 V. The enhanced oxidative stability of [NPPA][TFSI] is attributed to the electron‐withdrawing fluorinated ether functional group in the molecular backbone.^[^ ^56^, ^57^, ^58^, ^59^ ^]^ The high electrochemical stability was retained after the addition of Np_2_F, with a voltage window of 5.4 V (−3.6, 1.8 V vs. Ag/Ag^+^) for Np_2_F/[MNPA][TFSI] and 5.7 V (−3.6, 2.1 V vs. Ag/Ag^+^) for Np_2_F/[NPPA][TFSI]. This high oxidation stability could, in the future, enable electrodes such as Ag to be used in fluoride ion batteries. As a preliminary examination, the cyclic voltammograms using Ag wire as the working electrode in 0.1 M Np_2_F/[MNPA][TFSI] are given in Figure 4b. In the first cycle, the oxidation peak is observed at −0.4 V, corresponding to Ag oxidation (fluorination). The reduction (defluorination) peak is observed at −1.0 V. Subsequent cyclic voltammograms overlap to a significant extent, demonstrating that stable defluorination/fluorination reactions of the Ag electrode are realized in the Np_2_F/[MNPA][TFSI] electrolyte. The high oxidation stability could also facilitate using high‐capacity electrodes, such as copper, and promote the development of new cathode materials that operate at even higher potentials.

### NMR Dynamics

**Figure 5:**
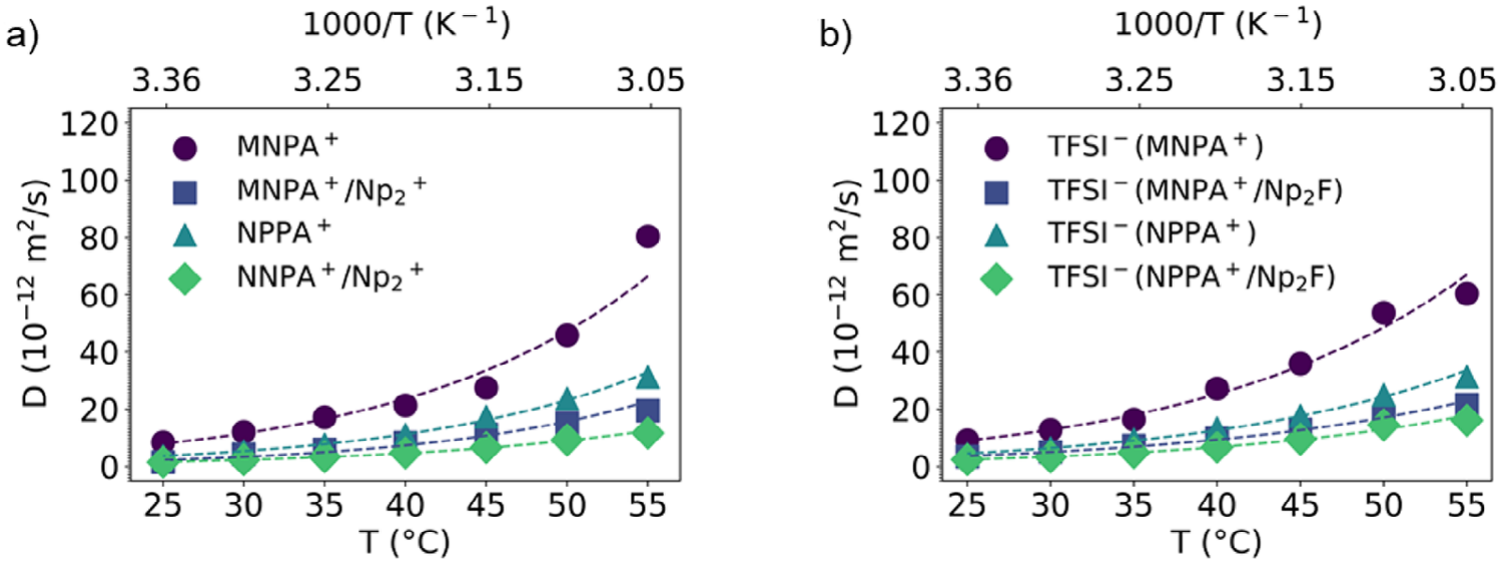
Temperature dependence of the diffusion coefficients, *D*, for a) cations and b) anions.

NMR diffusion and relaxation measurements were conducted to understand how the alkoxy substituents influence the viscosity and diffusivity of the ionic liquid electrolytes. In particular, diffusion‐ordered spectroscopy (DOSY) measurements provide helpful information about the translational motion of the ions in the ionic liquid‐based electrolytes. The diffusion coefficients, *D*, and viscosities, *η*, of [MNPA][TFSI] and [NPPA][TFSI] with and without 0.5 M Np_2_F were determined from 25 to 55 °C. Figure 5a,b shows the dependence of *D* on temperature. The data was fitted with the Arrhenius equation to obtain the activation energy barriers for ion translational motion, *E*_cation,_ and *E*_anion_. The diffusion coefficients, viscosities, and activation energies are listed in Table 1. The details are given in the Supporting Information.

**Table 1:** Diffusion coefficient (*D*), viscosity (*η*), and transference number of the anion (*t*_anion_) at 25 °C and activation energy (*E*).

	*D*_cation_	*D*_anion_	*D*_total_ ^c)^	*t*_anion_ ^d)^	*η*	*E*_cation_	*E*_anion_
	10^−12^ (m^2^ s^−1^)				(mPa⋅s)	(kJ mol^−1^)	(kJ mol^−1^)
[MNPA][TFSI]	8.45	9.31	17.76	0.52	235	74.6	50.5
Np_2_F/[MNPA][TFSI]	1.55^a)^	3.44^b)^	4.99	0.69	260	51.8	46.9
[NPPA][TFSI]	3.67	4.27	7.94	0.54	353	57.2	50.9
Np_2_F/[NPPA][TFSI]	1.52^a)^	2.41^b)^	3.93	0.61	550	52.2	50.5

^a)^ As many peaks overlap in the ^1^H NMR spectra of the ionic liquid electrolytes, these diffusion coefficients are an average of the two cation species.

^b)^ The peaks of the fluoride anion and TFSI overlap in the ^19^F NMR spectra, so the diffusion coefficients are an average over the two cation species.

^c)^ *D*_total_ = *D*_cation _+ *D*_anion_.

^d)^ *t*_anion_ *= D*_anion_/*D*_total_.

For [MNPA][TFSI] and [NPPA][TFSI], the TFSI anion diffuses faster than MNPA or NPPA cations, with anion transference numbers *t*_anion_ of 0.52 for [MNPA][TFSI] and 0.54 for [NPPA][TFSI]. The values are slightly higher for the ionic liquid‐based electrolytes, 0.69 for Np_2_F/[MNPA][TFSI] and 0.61 for Np_2_F/[NPPA][TFSI]. This is due to the interactions between the fluoride anion and the ammonium cations being stronger than the corresponding interaction between the ammonium cation and TFSI. The activation energy of the anions in ionic liquids containing Np_2_F is lower than that of neat ionic liquids. Overall, these values indicate the higher mobility of the anions than the cations in ionic liquid electrolytes containing Np_2_F.

The total diffusion coefficient, *D*_total_ of [MNPA][TFSI] is 17.16 × 10^−12^ m^2^ s^−1^, diffusing faster than [NPPA][TFSI] with *D*_total_ of 7.94 × 10^−12^ m^2^ s^−1^. The same trend was observed for the ionic liquid electrolytes with Np_2_F and closely correlates with the measured viscosities. These results suggest electrolytes using [MNPA][TFSI] have higher mobility than those using [NPPA][TFSI]. The rationale for the higher mobility of electrolytes with [MNPA][TFSI] was evaluated by examining the spin−lattice (longitudinal, *T*_1_) and spin−spin (transverse, *T*_2_) relaxation times. *T*_1_ and *T*_2_ are sensitive to intra‐ and intermolecular relaxation mechanisms and can be used to probe the reorientation dynamics of the system.^[^ ^60^, ^61^, ^62^, ^63^, ^64^, ^65^, ^66^ ^]^ The *T*_1_/*T*_2_ ratio is a helpful parameter for investigating local order.^[^ ^67^, ^68^, ^69^ ^]^ For an isotropic fluid, the *T*_1_/*T*_2_ ratio equals one, while higher values indicate the presence of local structures.^[^ ^67^, ^68^, ^69^, ^70^ ^]^The *T*_1_/*T*_2_ ratios at room temperature are summarized in Table 2. The *T*_1_/*T*_2_ ratio for cations and anions in ionic liquid electrolytes with Np_2_F is higher than in ionic liquids without ammonium fluoride salt. Meanwhile, the *T*_1_/*T*_2_ ratio for Np_2_F/[NPPA][TFSI] is higher than for Np_2_F/[MNPA][TFSI]. Notably, the *T*_1_/*T*_2_ ratio for the TFSI anion in Np_2_F/[NPPA][TFSI] increases to over 10 after dissolving Np_2_F, indicating the presence of localized structures. These results are consistent with the stronger ion–ion interactions observed in the single‐crystal structures. (Figure 3) Although [NPPA][TFSI] with the trifluoroethoxy group has a higher solubility for fluoride salts, the associated increase in viscosity leads to lower diffusivity compared with [MNPA][TFSI]. Based on this evaluation, we conclude that Np_2_F/[MNPA][TFSI] is the more suitable electrolyte for fluoride‐ion batteries.

**Table 2:** Spin−lattice (longitudinal, *T*_1_) and spin−spin (transverse, *T*_2_) relaxation time at 25 °C.

	*T*_1_ (cation, ms)	*T*_2_ (cation, ms)	*T*_1_/*T*_2_ (cation)	*T*_1_ (anion, ms)	*T*_2_ (anion, ms)	*T*_1_/*T*_2_ (anion)
[MNPA][TFSI]	363	135	2.69	594	208	2.86
Np_2_F/[MNPA][TFSI]	357^a)^	95^a)^	3.76	669^b)^	90^b)^	7.43
[NPPA][TFSI]	255	79	3.23	557	117	4.76
Np_2_F/[NPPA][TFSI]	307^a)^	66^a)^	4.65	650^b)^	62^b)^	10.48

*T*_1_ and *T*_2_ were estimated using the inversion recovery and the Carr–Purcell–Meiboom–Gill (CPMG) method, respectively.

^a)^ As many peaks overlap in the ^1^H NMR spectra of the ionic liquid electrolytes, these relaxation times are the average of the two kinds of cations.

^b)^ In ^19^F NMR spectra, *T*_1_ and *T*_2_ of fluoride anions could not be determined due to the relevant peaks overlapping with those from the TFSI anions.

### Battery Performance

**Figure 6:**
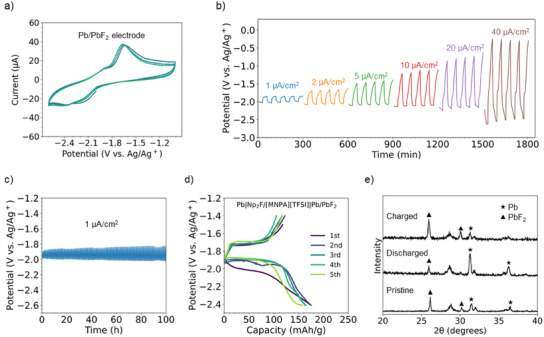
a) Cyclic voltammograms of the Pb/PbF_2_ electrode in 0.5 M Np_2_F/[MNPA][TFSI] electrolyte at 298 K with a 1 mV s^−1^ scan rate, b) voltage profiles of the Pb/PbF_2_ electrode upon charging/discharging at current densities of 1, 2, 5, 10, 20, 40 µA per cm^2^ in 0.5 M Np_2_F/[MNPA][TFSI] electrolyte with 30 min discharge and 30 min charge, c) long term voltage profiles of the Pb/PbF_2_ electrode upon charging/discharging 1 µA per cm^2^ in 0.5 M Np_2_F/[MNPA][TFSI] electrolyte with 30 min discharge and 30 min charge, d) charge/discharge curve (0.05 C) for the Pb/PbF_2_ electrode in 0.5 M Np_2_F/[MNPA][TFSI], and e) X‐ray diffraction patterns for the Pb/PbF_2_ electrode before and after charge/discharge (pristine, discharged, and charged).

The performance of a 0.5 M Np_2_F/[MNPA][TFSI] electrolyte was evaluated in a test cell at 25 °C using Pb/PbF_2_, Pb foil, and Ag/AgOTf electrode as the working electrode, counter electrode, and reference electrode, respectively. Cyclic voltammograms are shown in Figure 6a. In the first cycle, an oxidation peak is observed at −1.7 V versus Ag/Ag^+^, corresponding to the fluorination of Pb. The corresponding peak for reducing PbF_2_ to metallic Pb is at −2.2 V versus Ag/Ag^+^. These potentials remain constant over multiple cycles. To examine the stability under different current loading, charge/discharge voltage plateaus were measured at current densities of 1, 2, 5, 10, 20, and 40 µA per cm^2^ (Figure 6b). The cell remains relatively stable at 40 µA per cm^2^ charge/discharge current density. Long‐term cycling at 1 µA per cm^2^ could be maintained for over 100 h (Figure 6c). The overall performance of the same cell with 0.5 M Np_2_F/[NPPA][TFSI] electrolyte was less satisfactory, with charge/discharge voltage plateaus at 1 µA per cm^2^ resulting in higher overpotential (Figure S45). This may be attributed to the reactive nature of the highly acidic protons of the trifluoroethoxy group.

Returning to the 0.5 M Np_2_F/[MNPA][TFSI] electrolyte, the charge/discharge performance at 0.05 C is shown in Figure 6d. The initial discharge capacity was 175 mAh g^−1^, falling to 158 mAh g^−1^ after five cycles. The loss of capacity is most likely caused by the degradation of the Pb/PbF_2_ electrode. Irrespective of the electrode instability, defluorination/fluorination reactions were confirmed by structural characterization of the Pb/PbF_2_ electrode using X‐ray diffraction: As shown in Figure 6e, the peaks corresponding to PbF_2_ diminished after discharge, while the peak for Pb increased. The PbF_2_ peaks increased during the charging process, while the Pb peak decreased. This stable fluoride anion shuttling strongly suggests that Np_2_F/[MNPA][TFSI] is a promising electrolyte candidate for fluoride ion batteries.

## Conclusion

Two new quaternary ammonium TFSI ionic liquids, [MNPA][TFSI] and [NPPA][TFSI], based on 3‐alkoxy‐*N*,*N*,*N*,2,2‐pentamethylpropan‐1‐aminium backbone were designed and synthesized. No β protons were present on the quaternary cations, ensuring chemical stability against Hofmann elimination. Both salts are liquid at room temperature and, when mixed with ammonium fluoride salts, make promising electrolytes for FIBs with high ionic conductivity and good stability. Crucially, Np_2_F/[MNPA][TFSI] and Np_2_F/[NPPA][TFSI] electrolytes exhibited excellent electrochemical stability with a wide voltage window of 5.4 and 5.7 V, respectively. Stable defluorination/fluorination reactions are realized with the combination of Np_2_F/[MNPA][TFSI] electrolyte and a silver electrode. The good electrochemical stability could also facilitate using high‐capacity electrodes, such as copper, and promote the development of new cathode materials that operate at even higher potential. Battery performance using Np_2_F/[MNPA][TFSI] demonstrated a stable and reversible (de)fluorination reaction. Stable fluoride ion shuttling for up to 100 h was demonstrated.

These results on synthetic pathways to room‐temperature ion liquids offer crucial insights for developing advanced liquid electrolytes based on quaternary ammonium fluoride, paving the way for high‐performance fluoride batteries.

## Supporting Information

Synthetic details, data, and additional analysis of NMR experiments and electrochemical data, ^1^H, ^19^F, ^13^C NMR spectra for all compounds. Deposition Numbers 2393058 (for [MNPA][TFSI]) and 2393059 (for [NPPA][TFSI]) contain the supplementary crystallographic data for this paper. The joint Cambridge Crystallographic Data Centre and Fachinformationszentrum Karlsruhe Access Structures service provides these data free of charge. The authors have cited additional references within the Supporting Information.^[^ ^71^, ^72^, ^73^, ^74^, ^75^, ^76^, ^77^, ^78^, ^79^, ^80^, ^81^, ^82^, ^83^, ^84^, ^85^, ^86^, ^87^, ^88^, ^89^, ^90^, ^91^ ^]^

## Conflict of Interests

The authors declare no conflict of interest.

## Supporting information



Supporting Information

Supporting Information

## Data Availability

The data that support the findings of this study are available from the corresponding author upon reasonable request.

## References

[1] A. W. Xiao , G. Galatolo , M. Pasta , Joule 2021, 5, 2823–2844.

[2] F. Gschwind , G. Rodriguez‐Garcia , D. J. S. Sandbeck , A. Gross , M. Weil , M. Fichtner , N. Hörmann , J. Fluor. Chem. 2016, 182, 76–90.

[3] M. A. Nowroozi , I. Mohammad , P. Molaiyan , K. Wissel , A. R. Munnangi , O. Clemens , J. Mater. Chem. A 2021, 9, 5980–6012.

[4] S. V. Gopinadh , P. V. R. L. Phanendra , B. John , T. D. Mercy , Sustain. Mater. Technol. 2022, 32, e00436.

[5] M. A. Reddy , M. Fichtner , J. Mater. Chem. 2011, 21, 17059.

[6] D. T. Thieu , M. H. Fawey , H. Bhatia , T. Diemant , V. S. K. Chakravadhanula , R. J. Behm , C. Kübel , M. Fichtner , Adv. Funct. Mater. 2017, 27, 1701051.

[7] Y. Wang , X. Yang , Y. Meng , Z. Wen , R. Han , X. Hu , B. Sun , F. Kang , B. Li , D. Zhou , C. Wang , G. Wang , Chem. Rev. 2024, 124, 3494–3589. 38478597 10.1021/acs.chemrev.3c00826

[8] M. Zhang , X. Cao , Y. Hao , H. Wang , J. Pu , B. Chi , Z. Shen , Energy Rev. 2024, 3, 100083.

[9] J. Li , M. Li , H. Weng , S. Xu , Energy Technol. 2025, 13, 2401374.

[10] F. Gschwind , Z. Zao‐Karger , M. Fichtner , J. Mater. Chem. A 2013, 2, 1214–1218.

[11] O. Alshangiti , G. Galatolo , G. J. Rees , H. Guo , J. A. Quirk , J. A. Dawson , M. Pasta , ACS Energy Lett. 2023, 8, 2668–2673. 37324537 10.1021/acsenergylett.3c00493 PMC10262201

[12] S. Kawauchi , H. Nakamoto , R. Takekawa , T. Kobayashi , T. Abe , ACS Appl. Energy Mater. 2022, 5, 2096–2103.

[13] K. Okazaki , Y. Uchimoto , T. Abe , Z. Ogumi , ACS Energy Lett. 2017, 2, 1460–1464.

[14] T. Yamamoto , K. Matsumoto , R. Hagiwara , T. Nohira , ACS Appl. Energy Mater. 2019, 2, 6153–6157.

[15] V. K. Davis , C. M. Bates , K. Omichi , B. M. Savoie , N. Momčilović , Q. Xu , W. J. Wolf , M. A. Webb , K. J. Billings , N. H. Chou , S. Alayoglu , R. K. McKenney , I. M. Darolles , N. G. Nair , A. Hightower , D. Rosenberg , M. Ahmed , C. J. Brooks , T. F. Miller , R. H. Grubbs , S. C. Jones , Science 2018, 362, 1144–1148. 30523107 10.1126/science.aat7070

[16] G. Galatolo , O. Alshangiti , C. Di Mino , G. Matthews , A. W. Xiao , G. J. Rees , M. Schart , Y. A. Chart , L. F. Olbrich , M. Pasta , ACS Energy Lett. 2024, 9, 85–92. 38230375 10.1021/acsenergylett.3c02228 PMC10789089

[17] Y. Yu , M. Lei , C. Li , Mater. Horiz 2024, 11, 480–489. 37965817 10.1039/d3mh01039b

[18] R. Yaokawa , T. Shiga , S. Moribe , K. Mukai , RSC Adv. 2022, 12, 31786–31791. 36380965 10.1039/d2ra05753k PMC9639212

[19] Y. Yu , A. Lin , M. Lei , C. Lai , C. Wu , Y.‐Y. Sun , C. Li , ACS Energy Lett. 2024, 9, 1008–1016.

[20] P. Zou , C. Wang , Y. He , H. L. Xin , R. Lin , Nano Lett. 2024, 24, 5429–5435. 38682885 10.1021/acs.nanolett.4c00244

[21] C. Rongeat , M. Anji Reddy , R. Witter , M. Fichtner , ACS Appl. Mater. Interfaces 2014, 6, 2103–2110. 24444763 10.1021/am4052188

[22] Y. Yu , M. Lei , D. Li , C. Li , Adv. Energy Mater. 2023, 13, 2203168.

[23] I. Mohammad , R. Witter , M. Fichtner , M. A. Reddy , ACS Appl. Energy Mater. 2018, 1, 4766–4775.

[24] K. Shimoda , Y. Morita , K. Noi , T. Fukunaga , Z. Ogumi , T. Abe , ACS Energy Lett. 2023, 8, 2570–2575.

[25] H. Miki , K. Yamamoto , H. Nakaki , T. Yoshinari , K. Nakanishi , S. Nakanishi , H. Iba , J. Miyawaki , Y. Harada , A. Kuwabara , Y. Wang , T. Watanabe , T. Matsunaga , K. Maeda , H. Kageyama , Y. Uchimoto , J. Am. Chem. Soc. 2024, 146, 3844–3853. 38193701 10.1021/jacs.3c10871

[26] V. Vanita , A. Iqbal Waidha , S. Vasala , P. Puphal , R. Schoch , P. Glatzel , M. Bauer , O. Clemens , J. Mater. Chem. A 2024, 12, 8769–8784.

[27] Q. Nie , Y. Hao , L. Cheng , Y. Fu , G. Wang , M. Zhang , Z. Shen , Solid State Ion 2024, 405, 116454.

[28] J. Liu , L. Yi , X. Chen , D. Li , S. Ni , J. Xia , L. Yang , X. Wang , Sustain. Mater. Technol. 2024, 39, e00810.

[29] T. Tan , R. Murdey , S. Sumitomo , T. Nakamura , M. A. Truong , A. Wakamiya , Chem. Mater. 2024, 36, 4553–4560.

[30] T. Tan , R. Murdey , S. Sumitomo , A. Wakamiya , Sustain. Energy Fuels 2025, 9, 1525–1533.

[31] X. Liu , A. Mariani , T. Diemant , M. E. Di Pietro , X. Dong , A. Mele , S. Passerini , Adv. Mater. 2024, 36, 2309062. 10.1002/adma.202309062 37956687

[32] G.‐T. Kim , S. S. Jeong , M.‐Z. Xue , A. Balducci , M. Winter , S. Passerini , F. Alessandrini , G. B. Appetecchi , J. Power Sources 2012, 199, 239–246.

[33] A. Eftekhari , Y. Liu , P. Chen , J. Power Sources 2016, 334, 221–239.

[34] X. Ma , J. Yu , Y. Hu , J. Texter , F. Yan , Ind. Chem. Mater. 2023, 1, 39–59.

[35] G. A. Giffin , J. Mater. Chem. A 2016, 4, 13378–13389.

[36] M. Liu , S. Zhang , E. R. H. van Eck , C. Wang , S. Ganapathy , M. Wagemaker , Nat. Nanotechnol. 2022, 17, 959–967. 35864168 10.1038/s41565-022-01162-9

[37] M. Watanabe , M. L. Thomas , S. Zhang , K. Ueno , T. Yasuda , K. Dokko , Chem. Rev. 2017, 117, 7190–7239. 28084733 10.1021/acs.chemrev.6b00504

[38] E. Piatti , L. Guglielmero , G. Tofani , A. Mezzetta , L. Guazzelli , F. D'Andrea , S. Roddaro , C. S. Pomelli , J. Mol. Liq. 2022, 364, 120001.

[39] N. de Vos , C. Maton , C. V. Stevens , ChemElectroChem 2014, 1, 1258–1270.

[40] S. Kazemiabnavi , Z. Zhang , K. Thornton , S. Banerjee , J. Phys. Chem. B 2016, 120, 5691–5702. 27266487 10.1021/acs.jpcb.6b03433

[41] H. Sun , S. G. DiMagno , J. Am. Chem. Soc. 2005, 127, 2050–2051. 15713075 10.1021/ja0440497

[42] G. B. Appetecchi , M. Montanino , D. Zane , M. Carewska , F. Alessandrini , S. Passerini , Electrochim. Acta 2009, 54, 1325–1332.

[43] K. Yuyama , G. Masuda , H. Yoshida , T. Sato , J. Power Sources 2006, 162, 1401–1408.

[44] A. I. Bhatt , I. May , V. A. Volkovich , M. E. Hetherington , B. Lewin , R. C. Thied , N. Ertok , J. Chem. Soc. Dalton Trans. 2002, 4532–4534.

[45] L. J. A. Siqueira , M. C. C. Ribeiro , J. Phys. Chem. B 2009, 113, 1074–1079. 19119804 10.1021/jp807833a

[46] J. H. Lee , J.‐B. Ryu , A. S. Lee , W. Na , H.‐S. Yoon , W.‐J. Kim , C. M. Koo , Electrochim. Acta 2016, 222, 1847–1852.

[47] A. Tsurumaki , H. Ohno , S. Panero , M. A. Navarra , Electrochim. Acta 2019, 293, 160–165.

[48] J. Wang , Y. Li , C. Guo , Y. Zhao , J. Tong , J. Mol. Liq. 2022, 349, 118200.

[49] Z. J. Chen , T. Xue , J.‐M. Lee , RSC Adv. 2012, 2, 10564–10574.

[50] Y. K. J. Bejaoui , F. Philippi , H.‐G. Stammler , K. Radacki , L. Zapf , N. Schopper , K. Goloviznina , K. A. M. Maibom , R. Graf , J. A. P. Sprenger , R. Bertermann , H. Braunschweig , T. Welton , N. V. Ignat'ev , M. Finze , Chem. Sci. 2023, 14, 2200–2214. 36845914 10.1039/d2sc06758g PMC9945419

[51] A. R. Choudhury , N. Winterton , A. Steiner , A. I. Cooper , K. A. Johnson , CrystEngComm 2006, 8, 742–745.

[52] A. R. Choudhury , N. Winterton , A. Steiner , A. I. Cooper , K. A. Johnson , J. Am. Chem. Soc. 2005, 127, 16792–16793. 16316218 10.1021/ja055956u

[53] G. R. Desiraju , J. Am. Chem. Soc. 2013, 135, 9952–9967. 23750552 10.1021/ja403264c

[54] A.‐V. Mudring , Aust. J. Chem. 2010, 63, 544.

[55] R. Hayes , G. G. Warr , R. Atkin , Chem. Rev. 2015, 115, 6357–6426. 26028184 10.1021/cr500411q

[56] K. A. See , H.‐L. Wu , K. C. Lau , M. Shin , L. Cheng , M. Balasubramanian , K. G. Gallagher , L. A. Curtiss , A. A. Gewirth , ACS Appl. Mater. Interfaces 2016, 8, 34360–34371. 27998132 10.1021/acsami.6b11358

[57] X. Fan , L. Chen , O. Borodin , X. Ji , J. Chen , S. Hou , T. Deng , J. Zheng , C. Yang , S.‐C. Liou , K. Amine , K. Xu , C. Wang , Nat. Nanotechnol. 2018, 13, 715–722. 30013215 10.1038/s41565-018-0183-2

[58] S. Chen , J. Zheng , D. Mei , K. S. Han , M. H. Engelhard , W. Zhao , W. Xu , J. Liu , J.‐G. Zhang , Adv. Mater. 2018, 30, 1706102. 10.1002/adma.201706102 29575163

[59] O. Borodin , Curr. Opin. Electrochem. 2019, 13, 86–93.

[60] S. H. Chung , R. Lopato , S. G. Greenbaum , H. Shirota , E. W. Castner , J. F. Wishart , J. Phys. Chem. B 2007, 111, 4885–4893. 17441766 10.1021/jp071755w

[61] N. Bloembergen , E. M. Purcell , R. V. Pound , Phys. Rev. 1948, 73, 679–712.

[62] P. M. Richardson , A. M. Voice , I. M. Ward , J. Chem. Phys. 2013, 139, 214501. 24320385 10.1063/1.4832038

[63] R. Nanda , K. Damodaran , Magn. Reson. Chem. 2018, 56, 62–72. 28921712 10.1002/mrc.4666

[64] V. P. Ananikov , Chem. Rev. 2011, 111, 418–454. 20973480 10.1021/cr9000644

[65] R. Giernoth , in Ion. Liq. (Ed.: B. Kirchner ), Springer Berlin Heidelberg, Berlin, Heidelberg 2010.

[66] R. C. Remsing , G. Hernandez , R. P. Swatloski , W. W. Massefski , R. D. Rogers , G. Moyna , J. Phys. Chem. B 2008, 112, 11071–11078. 18693699 10.1021/jp8042895

[67] M. E. Di Pietro , F. Castiglione , A. Mele , J. Phys. Chem. B 2020, 124, 2879–2891. 32186377 10.1021/acs.jpcb.0c00026 PMC7997561

[68] V. Klimavicius , V. Bacevicius , Z. Gdaniec , V. Balevicius , J. Mol. Liq. 2015, 210, 223–226.

[69] P. G. Gordon , D. H. Brouwer , J. A. Ripmeester , ChemPhysChem 2010, 11, 260–268. 19924756 10.1002/cphc.200900624

[70] D. D. Traficante , in eMagRes, John Wiley & Sons, Ltd, 2007.

[71] M. D. Hanwell , D. E. Curtis , D. C. Lonie , T. Vandermeersch , E. Zurek , G. R. Hutchison , J. Cheminformatics 2012, 4, 17. 10.1186/1758-2946-4-17 PMC3542060 22889332

[72] G. Knizia , J. E. M. N. Klein , Angew. Chem., Int. Ed. 2015, 54, 5518–5522. 10.1002/anie.201410637 25737294

[73] G. Knizia , J. Chem. Theory Comput. 2013, 9, 4834–4843. 26583402 10.1021/ct400687b

[74] G. M. Sheldrick , Acta Crystallogr. Sect. Found. Adv. 2015, 71, 3–8. 10.1107/S2053273314026370 PMC4283466 25537383

[75] G. M. Sheldrick , Acta Crystallogr. Sect. C Struct. Chem. 2015, 71, 3–8. 25567568 10.1107/S2053229614024218 PMC4294323

[76] O. V. Dolomanov , L. J. Bourhis , R. J. Gildea , J. A. K. Howard , H. Puschmann , J. Appl. Crystallogr. 2009, 42, 339–341. 10.1107/S0021889811041161 PMC3236671 22199401

[77] S. Grimme , S. Ehrlich , L. Goerigk , J. Comput. Chem. 2011, 32, 1456–1465. 21370243 10.1002/jcc.21759

[78] S. Grimme , J. Antony , S. Ehrlich , H. Krieg , J. Chem. Phys. 2010, 132, 154104. 20423165 10.1063/1.3382344

[79] F. Weigend , R. Ahlrichs , Phys. Chem. Chem. Phys. 2005, 7, 3297–3305. 16240044 10.1039/b508541a

[80] F. Weigend , Phys. Chem. Chem. Phys. 2006, 8, 1057–1065. 16633586 10.1039/b515623h

[81] F. Neese , WIREs Comput. Mol. Sci. 2022, 12, e1606.

[82] H. Konishi , T. Minato , T. Abe , Z. Ogumi , Chem. Lett. 2018, 47, 1346–1349.

[83] H. Konishi , R. Takekawa , T. Minato , Z. Ogumi , T. Abe , Chem. Phys. Lett. 2020, 755, 137785.

[84] A. C. Kucuk , T. Minato , T. Yamanaka , T. Abe , J. Mater. Chem. A 2019, 7, 8559–8567.

[85] H. Konishi , T. Minato , T. Abe , Z. Ogumi , J. Electrochem. Soc. 2017, 164, A3702.

[86] A. Celik Kucuk , T. Abe , J. Fluor. Chem. 2020, 240, 109672.

[87] D. Li , G. Li , Y. Yu , C. Li , Adv. Mater 2025, 37, 2415106, accepted, 10.1002/adma.202415106. 39962814

[88] Z. Fu , X. Yang , Y. Tian , X. Hu , Y. Wang , L. Lin , F. Kang , G. Wang , B. Li , D. Zhou , Energy Storage Mater. 2024, 70, 103533.

[89] K. Okazaki , H. Nakamoto , T. Yamanaka , T. Fukunaga , Z. Ogumi , T. Abe , Chem. Mater. 2022, 34, 8280–8288.

[90] O. Alshangiti , G. Galatolo , C. Di Mino , T. F. Headen , J. Christianson , S. Merotto , G. J. Rees , Y. Delavoux , M. Swadźba‐Kwaśny , M. Pasta , ACS Energy Lett. 2024, 9, 6104–6108. 39698338 10.1021/acsenergylett.4c02663 PMC11651116

[91] Jmol: an open‐source Java viewer for chemical structures in 3D. http://www.jmol.org/.

