# Supplementary material for: Tailored 3‐Alkoxy‐N,N,N,2,2‐Pentamethylpropan‐1‐Ammonium Bis(trifluoromethylsulfonyl)Imide Ionic Liquids for Room‐Temperature Fluoride‐Ion Batteries
Source: Angew Chem Int Ed Engl. 2025 Apr 14;64(23):e202422299. doi: 10.1002/anie.202422299 (PMC12124448; doi:10.1002/anie.202422299)
Supplement: Supplementary file 1 — Supporting Information [file ANIE-64-e202422299-s002.docx]

Supporting Information for

# Tailored 3-Alkoxy-*N*,*N*,*N*,2,2-Pentamethylpropan-1-Ammonium Bis(trifluoromethylsulfonyl)imide Ionic Liquids for Room-Temperature Fluoride-Ion Batteries

Tiancheng Tan^1^, Richard Murdey^1^, Shunsuke Sumitomo^2^, Kazuyuki Sato,^2^ Takeshi Abe,^3^ and Atsushi Wakamiya^1^*

^1^Institute for Chemical Research, Kyoto University, Gokasho, Uji, Kyoto 611-0011, Japan

^2^Office of Institutional Advancement and Communications, Kyoto University, Gokasho, Uji, Kyoto 611-0011, Japan

^3^Graduate School of Engineering, Kyoto University, Nishikyo, Kyoto 615-8510, Japan

Contents

[Materials 3](#_Toc192134369)

[Single Crystal X-Ray Diffraction Analysis 3](#_Toc192134370)

[X-ray diffraction measurements 5](#_Toc192134371)

[Thermodynamic measurements 5](#_Toc192134372)

[NMR Measurements 5](#_Toc192134373)

[Electrochemical Measurements 6](#_Toc192134374)

[Theoretical Calculations 7](#_Toc192134375)

[Battery Performance 7](#_Toc192134376)

[Synthesis of [MNPA][TFSI] 8](#_Toc192134377)

[Synthesis of [NPPA][TFSI] 9](#_Toc192134378)

[Synthesis of TFSI Salts Based on Alkyl Ammonium Cations without β-Protons 10](#_Toc192134379)

[Thermogravimetric Analysis (TGA) 12](#_Toc192134380)

[Mulliken Atomic Charges 13](#_Toc192134381)

[NMR Charts 14](#_Toc192134382)

[Battery Performance using 0.5 M Np_2_F/[NPPA][TFSI] 35](#_Toc192134383)

[Impedance Data 35](#_Toc192134384)

[Differential Scanning Calorimetry (DSC) 36](#_Toc192134385)

[Voltage Window 37](#_Toc192134386)

[Author Contributions 44](#_Toc192134387)

[Reference 45](#_Toc192134388)

## Materials

3-(Dimethylamino)-2,2-dimethylpropan-1-ol, MeI, TsOCH_2_CF_3_, trimethyloxonium tetrafluoroborate, and LiTFSI were purchased from Tokyo Chemical Industry Co., Ltd. (TCI). [P_13_][TFSI], [DEME][TFSI], dehydrated solvents, dichloromethane, and THF were purchased from Kanto Chemical Co., Inc. Potassium hydride, KH, PbF_2_, and trimethyloxonium tetrafluoroborate were purchased from Sigma-Aldrich. Pb powder and Ag wire (2 mm dia) were purchased from Nilaco Corporation. All chemicals were used as received. Deuterated solvents were purchased from Eurisotop or Cambridge Isotope Laboratories, Inc. All deuterated solvents were used as received, except CD_3_CN, which was dried over 4 Å molecular sieves. Np_2_F was prepared based on an earlier report.^[1]^ Pb/PbF_2_ electrode was prepared based on an earlier report.^[2]^

## Single Crystal X-Ray Diffraction Analysis

Crystal structures were obtained using an XtaLAB Synergy R HyPix diffractometer using Mo K*_a_* radiation. The crystal was kept at a constant temperature of *T* = 100.00(10) K during data collection. The CrysAlisPro program (Rigaku, V1.171.43.64a, 2023) determined the total number of runs and images, which was also used to index the diffraction peaks. The structure was solved with the ShelXT structure solution program using the intrinsic phasing solution method and Olex2 as the graphical interface.^[3,4]^ The model was refined with version 2018/3 of ShelXL 2018/3 using least squares minimization.^[5]^

The supplementary crystallographic data for this paper were deposited as deposition numbers 2393058 (for [MNPA][TFSI]) and 2393059 (for [NPPA][TFSI]). The joint Cambridge Crystallographic Data Centre and Fachinformationszentrum Karlsruhe Access Structures service provides these data free of charge.

For [MNPA][TFSI], clear light colorless plate-shape crystal with dimensions 0.51×0.42×0.37 mm^3^ was mounted. Data were collected using an XtaLAB Synergy R HyPix diffractometer operating at *T* = 100.00(10) K. Data were measured using *ω* scans of 0.5° per frame for 1.5 s using Mo K*_a_* radiation (*λ* = 0.71073 Å). The diffraction pattern was indexed and the total number of runs and images was based on the strategy calculation from the program CrysAlisPro (Rigaku, V1.171.42.72a, 2022) The maximum resolution that was achieved was 2*θ* = 50.1° (0.84 Å). The diffraction pattern was indexed and the total number of runs and images was based on the strategy calculation from the program CrysAlisPro (Rigaku, V1.171.42.72a, 2022) and the unit cell was refined using CrysAlisPro (Rigaku, V1.171.42.72a, 2022). A total of 13934 reflections were measured, 3359 were independent (*R_int_* = 0.0511). All hydrogen atoms were placed using AFIX instructions. The C alerts in the CIF Check are caused by the residual peaks of sulfur. Crystal Data of [MNPA][TFSI] (CCDC: 2393058), C_11_H_22_F_6_N_2_O_5_S_2_, *M_r_*= 440.42, monoclinic, *P*2_1_/*n*, *a* = 12.0990(8) Å, *b* = 12.1838(7) Å, *c*= 13.3584(9) Å, *β* = 105.221(7)°, *α* = γ = 90°, *V* = 1900.1(2) Å^3^, *T* = 100.00(10) K, *Z* = 4, *Z'* = 1, μ = 0.360 mm^‑1^. The refinement converged to *wR_2_* = 0.0888 (all data), *R_1_* = 0.0419 (I > 2(I)), GOF = 1.039.

For [NPPA][TFSI], clear light colorless plate-shape crystal with dimensions 0.17×0.16×0.14 mm^3^ was mounted. Data were collected using an XtaLAB Synergy R HyPix diffractometer operating at *T* = 100.00(10) K. Data were measured using *ω* scans of 0.5° per frame for 0.5 s using Mo K*_a_* radiation (*λ* = 0.71073 Å). The diffraction pattern was indexed and the total number of runs and images was based on the strategy calculation from the program CrysAlisPro (Rigaku, V1.171.42.72a, 2022) The maximum resolution that was achieved was 2*θ* = 50.7° (0.83 Å). The diffraction pattern was indexed and the total number of runs and images was based on the strategy calculation from the program CrysAlisPro (Rigaku, V1.171.42.72a, 2022) and the unit cell was refined using CrysAlisPro (Rigaku, V1.171.42.72a, 2022). A total of 40757 reflections were measured, 7582 were independent (*R_int_* = 0.0457). The structure was refined with disorder models for CF_3_ groups. The occupancies of (C0A, F1A, F2A, F3A: C0B, F1B, F2B, F3B), (F4A, F5A, F6A: F4B, F5B, F6B), (F7A, F8A, F9A: F7B, F8B, F9B), (F10A, F11A, F12A: F10B, F11B, F12B) and (F13A, F14A, F15A: F13B, F14B, F15B) were refined as (0.63:0.37), (0.92:0.08), (0.92:0.08), (0.72:0.28) and (0.91:0.09), respectively. All hydrogen atoms were placed using AFIX instructions. Crystal Data of [NPPA][TFSI] (CCDC: 2393059), C_12_H_21_F_9_N_2_O_5_S_2_, *M_r_*= 508.43, triclinic, *P*-1, *a* = 9.5235(3) Å, *b* = 13.7392(3) Å, *c*= 17.2461(3) Å, *α* = 76.384(2)°, *β* = 76.799(2)°, γ = 85.756(5)°, *V* = 2134.71(9) Å^3^, *T* = 100.00(10) K, *Z* = 4, *Z'* = 2, μ = 0.352 mm^‑1^. The refinement converged to *wR_2_* = 0.2077 (all data), *R_1_* = 0.0771 (I > 2(I)), GOF = 1.056.

## X-ray Diffraction Measurements

X-ray diffraction (XRD) measurements were performed on a Rigaku RINT 2500 (Rigaku Co.) with Cu Kα radiation (λ= 1.5406 Å). The power supply was operated at 300 mA and 40 kV.

## Thermodynamic Measurements

Thermogravimetric analysis (TGA) was performed on a Shimadzu TGA-50 apparatus (Shimadzu Co.). The scan rate was initially 10 °C/min until the temperature reached 250 °C, after which it was reduced to 1 °C/min until the temperature reached 400 °C.

Differential scanning calorimetry (DSC). performed on a Shimadzu DSC-60 Plus apparatus (Shimadzu Co.). The scan rate was 3 °C/min.

## NMR Measurements

The ^1^H, ^13^C, and ^19^F NMR spectra were recorded with a Bruker Advance II 400 MHz spectrometer equipped with a BBFO probe. Chemical shifts are reported in δ ppm using a residual protonated solvent in the deuterated solvents for ^1^H as an internal standard. Deuterated solvent peaks were used as the internal standard for ^13^C NMR, whereas CFCl_3_ (0 ppm) was used as the external standard for ^19^F NMR. Sample solutions were prepared in the argon gas-filled glove box (H_2_O < 0.1 ppm, O_2_ < 0.1 ppm) for DOSY and relaxation NMR measurements. Wilmad low pressure/vacuum NMR tubes (LPV) were used.

Diffusion coefficient (m^2^ s^−1^) was obtained from the α protons on ammonium cation (^1^H) or TFSI anion (^19^F) using the following equation.

$$I=I_{0}\exp[-D\gamma^{2}g^{2}\delta^{2}\left( \Delta-\frac{\delta}{3} \right)]$$

$I$ is the observed intensity, $I_{0}$ is the reference intensity (unattenuated signal intensity), $\gamma$ is the gyromagnetic ratio of the observed nucleus, g is the gradient strength, δ is the length of the gradient, ∆ is the diffusion time.

Spin−Lattice (Longitudinal, *T*_1_) relaxation time was obtained from the α protons on ammonium cation (^1^H NMR) or TFSI anion (^19^F NMR) using the inversion recovery method with the following pulse sequence.

$$\pi(+x) - \tau- \pi/2(+x))$$

The magnitude of the free induction decay (FID), *M_z_*, after the π/2(+ x) pulse was measured as a function of time and then fitted using the following equation.

$$M_{z}=M_{0}(1-2A\exp\left[ -\frac{t}{T_{1}} \right])$$

*M_0_* is the magnetization at thermal equilibrium, and A is a fitting parameter.

Spin−Spin (Transverse, *T*_2_) relaxation time was obtained from the α protons on ammonium cation (^1^H NMR) or TFSI anion (^19^F NMR) using Carr-Purcell-Meiboom-Gill (CPMG) method with a different number of echo pulses the intensity can be mapped out as a function of time in the form of the following pulse sequence.

$$\pi/2(+x) - [\tau- \pi(+x)]_{n}$$

The magnitude of the FID, *M*_xy_, after each second echo was measured as a function of time then fitted using following equation.

$$M_{xy}=M_{0}\exp\left[ -\frac{t}{T_{2}} \right]$$

*M_0_* is the magnetisation at thermal equilibrium.

## Electrochemical Measurements

Linear sweep voltammetry and battery performance measurements were performed in the glove box (H_2_O < 0.1 ppm, O_2_ < 0.1 ppm) using an HZ-Pro analyzer (Hokuto Denko). A three-electrode cell using Pt as the working electrode and the counter electrode, A silver wire immersed in 1-methyl-1-propylpyrrolidinium bis(fluorosulfonyl)imide [P_13_][FSI] containing 0.1 M silver trifluoromethanesulfonate then placed in a compartment that was filled with [P_13_][FSI ] and immersed in electrolyte as the reference electrode (vs. Ag/Ag^+^). The potential of ferrocene in the ionic liquid was also determined (–0.38 V vs. Ag/Ag^+^) and added as a secondary axis. Due to fluoride anion reactivity towards ferrocene, this was done in the neat ionic liquid without adding fluoride salt.

## Theoretical Calculations

DFT calculations were carried out using the ORCA 5.0.3 software package.^[6]^ DLPNO-CCSD(T) method with the def2-TZVPP basis set to determine the energy associated with the ion-ion interactions and Mulliken atomic charges.^[7–10]^ Visualizations were generated by IboView, Jmol, and Avogadro.^[11–13]^

## Battery Performance

Pb/PbF_2_ Working electrode:

Pb, PbF_2_, and acetylene black (AB) were mixed in a 4:4:1 weight ratio. This mixture was then suspended in *N*-methylpyrrolidone (NMP) to form a smooth slurry with a 4:4:1:1 weight ratio of Pb, PbF_2_, AB, and polyvinylidene difluoride (PVDF). The slurry was coated onto a Ti current collector. The mixture was heated at under 80 °C for 0.5 hours, then dried overnight under vacuum. Electrodes with a 5-mm diameter were punched out from the dried mixture. The average weight of PbF_2_ per cell is 0.3 mg. The obtained capacities were calculated based on the weight of PbF_2_.

PbF_2_ Working electrode:

PbF_2_ and AB were mixed in a 7:1.5 weight ratio. This mixture was then suspended in *N*- NMP to form a smooth slurry with a 7:1.5:1.5 weight ratio of PbF_2_, AB, and PVDF. The slurry was coated onto a Ti current collector. The mixture was heated at under 80 °C for 0.5 hours, then dried under vacuum. Electrodes with a 5-mm diameter were punched out from the dried mixture. The average weight of PbF_2_ per cell is 0.4 mg.

Counter electrode:

Electrodes with a 5-mm diameter were punched out from a lead sheet.

Charge/discharge performance:

The charge/discharge performance of the Pb/PbF_2_ working electrodes was measured using a three-electrode cell (SB9 cells purchased from EC Frontier Co., Ltd) with PTFE separator. The entire cell assembly process was conducted inside a N_2_ glovebox. Pb was used as counter electrodes. A silver wire immersed in 1-methyl-1-propylpyrrolidinium bis(fluorosulfonyl)imide (P_13_-FSI) containing 0.1 M silver trifluoromethanesulfonate then placed in a compartment that was filled with P_13_-FSI and immersed in electrolyte as the reference electrode (vs. Ag/Ag^+^). The charge/discharge rate was measured at 0.05 C. The potential range of the charge/discharge cycle was set from −2.4 V to −1.4 V (vs. Ag/Ag^+^).

## Synthesis of [MNPA][TFSI]

### Scheme S1 Synthesis of [MNPA][TFSI]

KH (26.2 g, 30 wt% dispersion in mineral oil, 0.196 mol) was suspended in 300 mL THF at 0 °C and 3-(dimethylamino)-2,2-dimethylpropan-1-ol (**1**) (29.0 mL, 24.9 g, 0.190 mol) was slowly added. Methyl iodide (12.9 mL, 29.4 g, 0.209 mol) was slowly added at 0 °C. The mixture was stirred at room temperature for 18 hours. 3-Methoxy-*N*,*N*,2,2-tetramethylpropan-1-amine product (**2a**) (17.2 g, 0.119 mol) was purified by distillation at low pressure. The obtained amine (9.5 g, 0.065 mol) was slowly added to a suspension of Meerwein’s salt (trimethyloxonium tetrafluoroborate, 10.0 g, 0.0680 mol) in 150 mL dry dichloromethane. The suspension was stirred at room temperature for 2 hours, and LiTFSI (19.7 g, 0.0686 mol) was added into the solution and stirred at room temperature for 1 hour, then the reaction mixture was washed with water, and the solvent was removed by vacuum evaporation to obtain a colorless liquid which was further dried under vacuum at 80  °C for 14 hours to obtain [MNPA][TFSI] (26.7 g, 0.0607 mol) in 93% yield.

3-Methoxy-*N*,*N*,2,2-tetramethylpropan-1-amine: ^1^H NMR (400 MHz, CDCl_3_): δ3.17 (s, 3H), 2.97 (s, 2H), 2.14 (s, 6H), 2.01 (s, 2H), 0.73 (s, 6H); ^13^C NMR (101 MHz, CDCl_3_): δ 80.23, 67.42, 59.17, 48.85, 36.86, 23.64.

MNPABF_4_: ^1^H NMR (400 MHz, DMSO-*d*_6_): δ 3.30 (s, 2H), 3.28 (s, 3H), 3.21 (s, 2H), 3.13 (s, 9H), 1.08 (s, 6H); ^13^C NMR (101 MHz, DMSO-*d*_6_): δ 78.96, 72.58, 58.41, 54.57, 36.91, 24.94; ^19^F NMR (376 MHz, DMSO-*d*_6_): δ –148.

[MNPA][TFSI]: ^1^H NMR (400 MHz, DMSO-*d*_6_): δ 3.31 (s, 3H), 3.28 (s, 2H), 3.22 (s, 2H), 3.14 (s, 9H), 1.09 (s, 6H); ^13^C NMR (101 MHz, DMSO-*d*_6_): δ 119.62 (q, *J* =322 Hz), 78.92, 72.63, 58.34, 54.53, 36.87,24.89; ^19^F NMR (376 MHz, DMSO-*d*_6_): δ –79; HMRS (ESI): *m*/*z* calcd for C_9_H_22_ON^+^: 160.1696 [*M*]^+^; found: 160.1696.

## Synthesis of [NPPA][TFSI]

### Scheme S2 Synthesis of [NPPA][TFSI]

KH (20.0 g, 30 wt% dispersion in mineral oil, 0.150 mol) was suspended in 400 mL THF at 0 °C and 3-(dimethylamino)-2,2-dimethylpropan-1-ol (**1**) (29.0 mL, 24.9 g, 0.190 mol) was slowly added. 2,2,2-Trifluoroethyl 4-methylbenzenesulfonate (37.0 g, 0.168 mol) was then slowly added at 0 °C. The mixture was stirred at 70 °C for 18 hours. *N*,*N*,2,2-Tetramethyl-3-(2,2,2-trifluoroethoxy)propan-1-amine product (**2b**) (22.1 g, 0.104 mol) was purified by distillation at low pressure. **2b** (13.8 g, 0.0647 mol) was slowly added to a suspension of Meerwein’s salt (trimethyloxonium tetrafluoroborate, 10.0 g, 0.0680 mol) in 150 mL dry dichloromethane. The suspension was stirred at room temperature for 2 hours, LiTFSI (19.7 g, 0.0686 mmol) was added into the solution and stirred at room temperature for 1 hours, then the reaction mixture was washed with water, and the solvent was removed by vacuum evaporation to obtain a colorless liquid which was further dried under vacuum at 80  °C for 14 hours to obtain [NPPA][TFSI] (29.1 g, 0.0573 mol) in 88% yield.

*N*,*N*,2,2-Tetramethyl-3-(2,2,2-trifluoroethoxy)propan-1-amine: ^1^H NMR (400 MHz, CDCl_3_): δ 3.65 (q, 2H, *J* _H-F_=9.4 Hz), 3.22 (s, 2H), 2.14 (s, 6H), 2.03 (s, 2H), 0.75 (s, 6H); ^13^C NMR (101 MHz, CDCl_3_): δ 124.39 (q, *J* =280 Hz), 79.74, 69.04 (q, *J* =34 Hz), 66.95, 48.79, 37.16, 23.36; ^19^F NMR (376 MHz, CDCl_3_): δ –74 (t, *J* =9 Hz).

NPPABF_4_: ^1^H NMR (400 MHz, DMSO-*d*_6_): δ 4.12 (q, 2H, *J* =9.4 Hz), 3.52 (s, 2H), 3.32 (s, 2H), 3.15 (s, 9H), 1.11 (s, 6H); ^13^C NMR (101 MHz, DMSO-*d*_6_): δ 124.44 (q, *J* =280 Hz), 78.40, 72.24, 67.52 (q, *J* =33 Hz), 54.64, 37.01, 24.54; ^19^F NMR (376 MHz, DMSO-*d*_6_): δ–73 (t, *J* =9 Hz), –148.

[NPPA][TFSI]: ^1^H NMR (400 MHz, DMSO-*d*_6_): δ 4.11 (q, 2H, *J* _H-F_=9.4 Hz), 3.52 (s, 2H), 3.33 (s, 2H), 3.15 (s, 9H), 1.11 (s, 6H); ^13^C NMR (101 MHz, DMSO-*d*_6_): δ 125.10 (q, *J* =280 Hz), 119.09 (q, *J* =323 Hz), 78.41, 72.28, 67.56 (q, *J* =33 Hz), 54.65, 37.01, 24.52; ^19^F NMR (376 MHz, DMSO-*d*_6_): δ –73 (t, *J* =9 Hz), –79; HMRS (ESI): *m*/*z* calcd for C_10_H_21_OF_3_N^+^: 228.1570[*M*]^+^; found: 228.1561.

## Synthesis of TFSI Salts Based on Alkyl Ammonium Cations without β-Protons

### Scheme S3 Synthesis of [MeDMB][TFSI]

Ammonium borate salt [MeDMB][BF_4_] was prepared following established procedures in previous report.^[14]^ [MeDMB][BF_4_] (20 mg, 86 μmol) was dissolved in dichloromethane, and LiTFSI (27 mg, 95 μmol) was added to the solution, which was then shaken. The conversion of BF_4_^–^ ions to TFSI ions was monitored using ^19^F NMR: If BF_4_^–^ ions were detected, additional LiTFSI was added until the impurity was no longer observed. The mixture was washed with water until no Li species were detected by ^7^Li NMR. After removing the solvents through vacuum evaporation, the crude product [MeDMB][TFSI] was further dried under vacuum at 90 °C for 18 hours, giving [MeDMB][TFSI] (23 mg, 71 μmol) as a colorless solid in 83% yield.

mp: 48 °C; ^1^H NMR (400 MHz, CD_2_Cl_2_): 3.22 (s, 2H), 3.21 (s, 9H), 1.48 (q, 2H, *J* =7.2 Hz), 1.17 (s, 6H), 0.92 (t, 3H, *J* =7.2 Hz); ^13^C NMR (101 MHz, CD_2_Cl_2_): δ 121.77 (q, *J* =321 Hz), 78.74, 53.60, 33.98, 30.17; ^19^F NMR (376 MHz, CD_2_Cl_2_): δ –78.

### Scheme S4 Synthesis of [Np_2_][TFSI]

Ammonium borate salt [Np_2_][BF_4_] was prepared following established procedures in previous report.^[14]^ [Np_2_][BF_4_] (20 mg, 73 μmol) was dissolved in dichloromethane, and LiTFSI (23 mg, 80 μmol) was added to the solution, which was then shaken. The conversion of BF_4_^–^ ions to TFSI ions was monitored using ^19^F NMR: If BF_4_^–^ ions were detected, additional LiTFSI was added until the impurity was no longer observed. The mixture was washed with water until no Li species were detected with ^7^Li NMR. After removing the solvents through vacuum evaporation, the crude product [Np_2_][TFSI] was further dried under vacuum at 90 °C for 18 hours, giving [Np_2_][TFSI] (27 mg, 71 μmol) as a colorless solid in 97% yield.

mp: 78 °C; ^1^H NMR (400 MHz, CDCl_3_): δ 3.27 (s, 4H), 3.20 (s, 6H), 1.18 (s, 18H); ^13^C NMR (101 MHz, CDCl_3_): δ 120.02 (q, *J* =321 Hz), 78.58, 58.00, 38.14, 36.83, 28.19, 9.62; ^19^F NMR (376 MHz, CDCl_3_): δ –79.

### Scheme S5 Synthesis of [NpDMB][TFSI]

Ammonium borate salt [NpDMB][BF_4_] was prepared following established procedures in previous report.^[14]^ [NpDMB][BF_4_] (20 mg, 70 μmol) was dissolved in dichloromethane, and LiTFSI (22 mg, 77 μmol) was added to the solution, which was then shaken. The conversion of BF_4_^–^ ions to TFSI ions was monitored using ^19^F NMR: If BF_4_^–^ ions were detected, additional LiTFSI was added until the impurity was no longer observed. The mixture was washed with water until no Li species were detected with ^7^Li NMR. After removing the solvents through vacuum evaporation, the crude product [NpDMB][TFSI] was further dried under vacuum at 90 °C for 18 hours, giving [NpDMB][TFSI] (25 mg, 66 μmol) as a colorless solid in 94% yield.

mp: 53 °C; ^1^H NMR (400 MHz, CDCl_3_): δ 3.31 (s, 2H), 3.29 (s, 2H), 3.25 (s, 6H), 1.48 (q, 2H, *J* =7.6 Hz), 1.22 (s, 9H), 1.18 (s, 6H), 0.92 (t, 3H, *J* =7.6 Hz); ^13^C NMR (101 MHz, CDCl_3_): δ 120.07 (q, *J* =321 Hz), 79.24,77.48, 54.04, 36.81, 35.86, 34.15, 30.40, 27.11,8.24; ^19^F NMR (376 MHz, CDCl_3_): δ –78.

## Thermogravimetric Analysis (TGA)


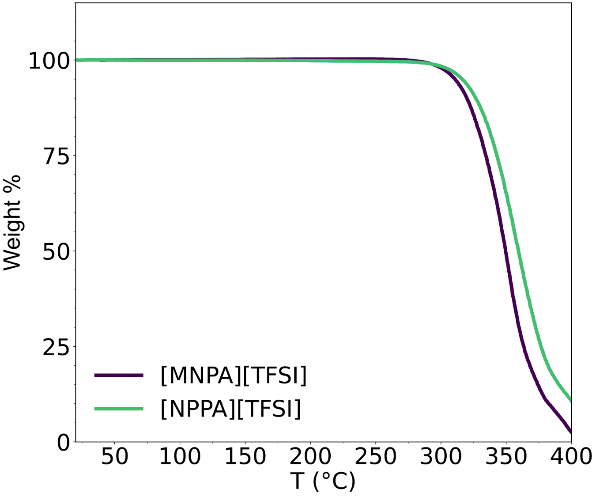


Figure S1. TGA trace of [MNPA][TFSI] and [NPPA][TFSI]. The scan rate was initially 10 °C/min until the temperature reached 250 °C, after which it was reduced to 1 °C/min until the temperature reached 400 °C.

## Mulliken Atomic Charges


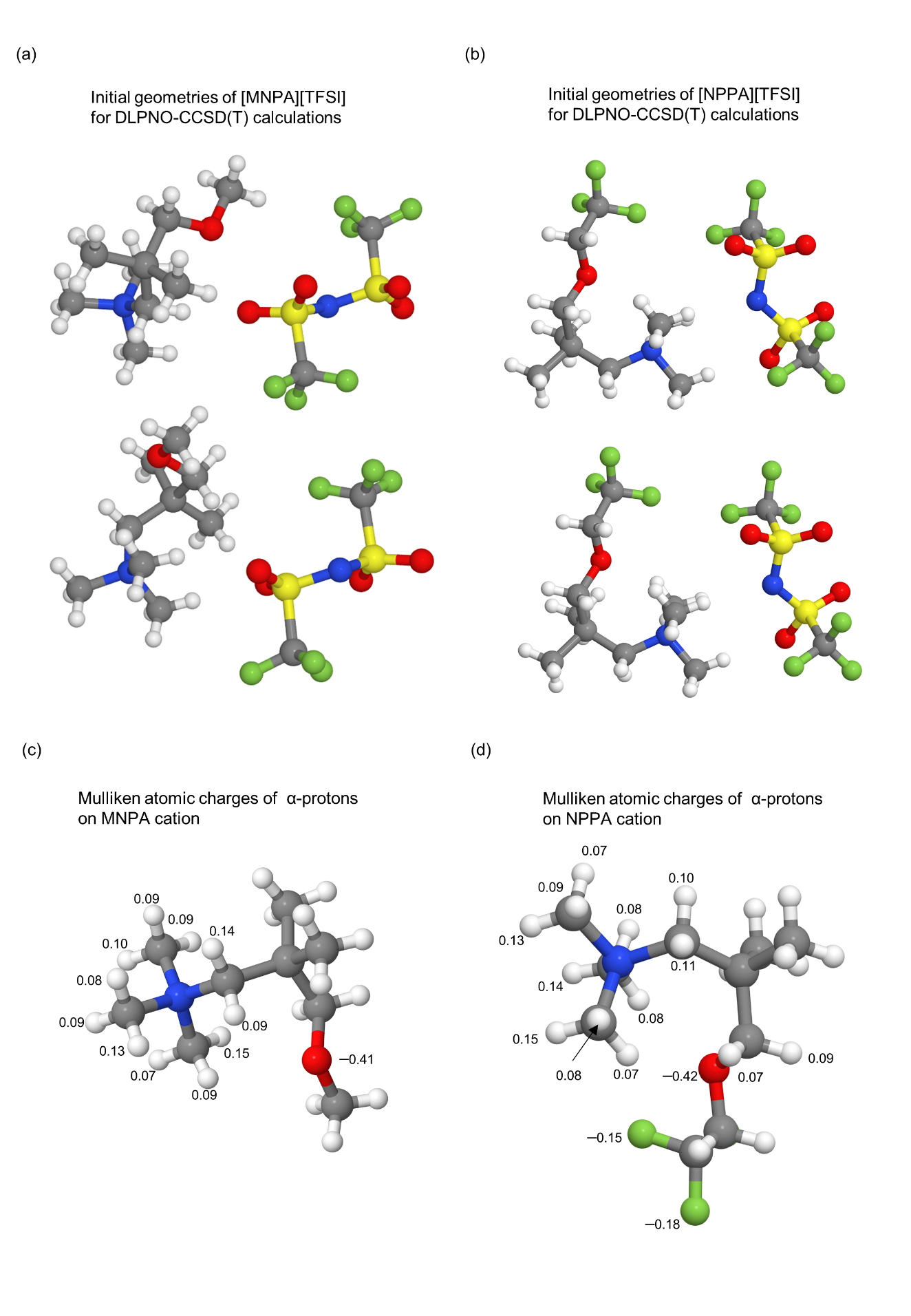


### Figure S2. Initial geometries for DLPNO-CCSD(T) calculations (a) [MNPA][TFSI], (b) [NPPA][TFSI], Mulliken atomic charges of α-protons on cations (c) MNPA cation, and (d) NPPA cation Other components are omitted for clarity.

## NMR Charts


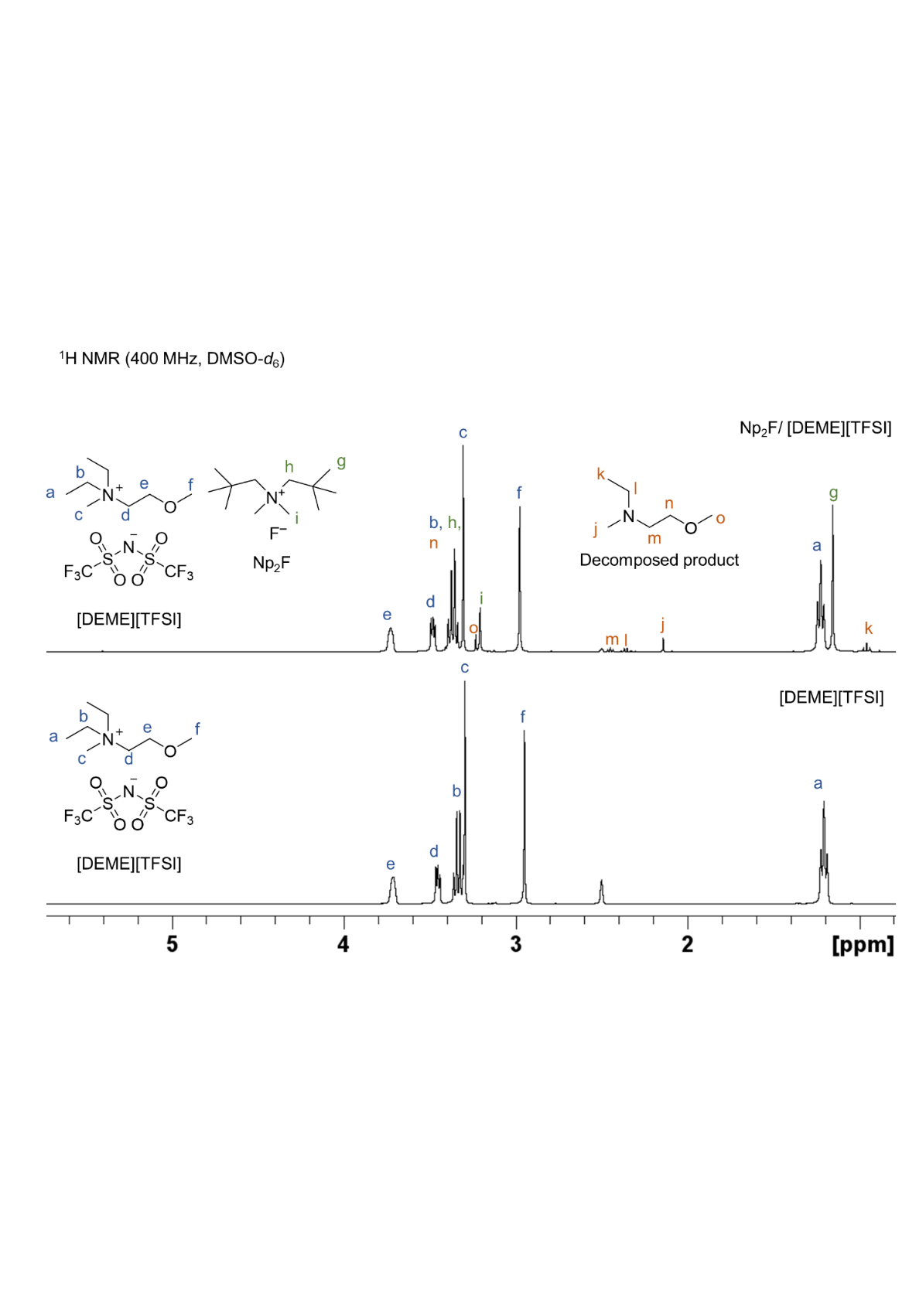


### Figure S3. ^1^HMNR of [DEME][TFSI] (down) and [DEME][TFSI]/Np_2_F (up) at room temperature.


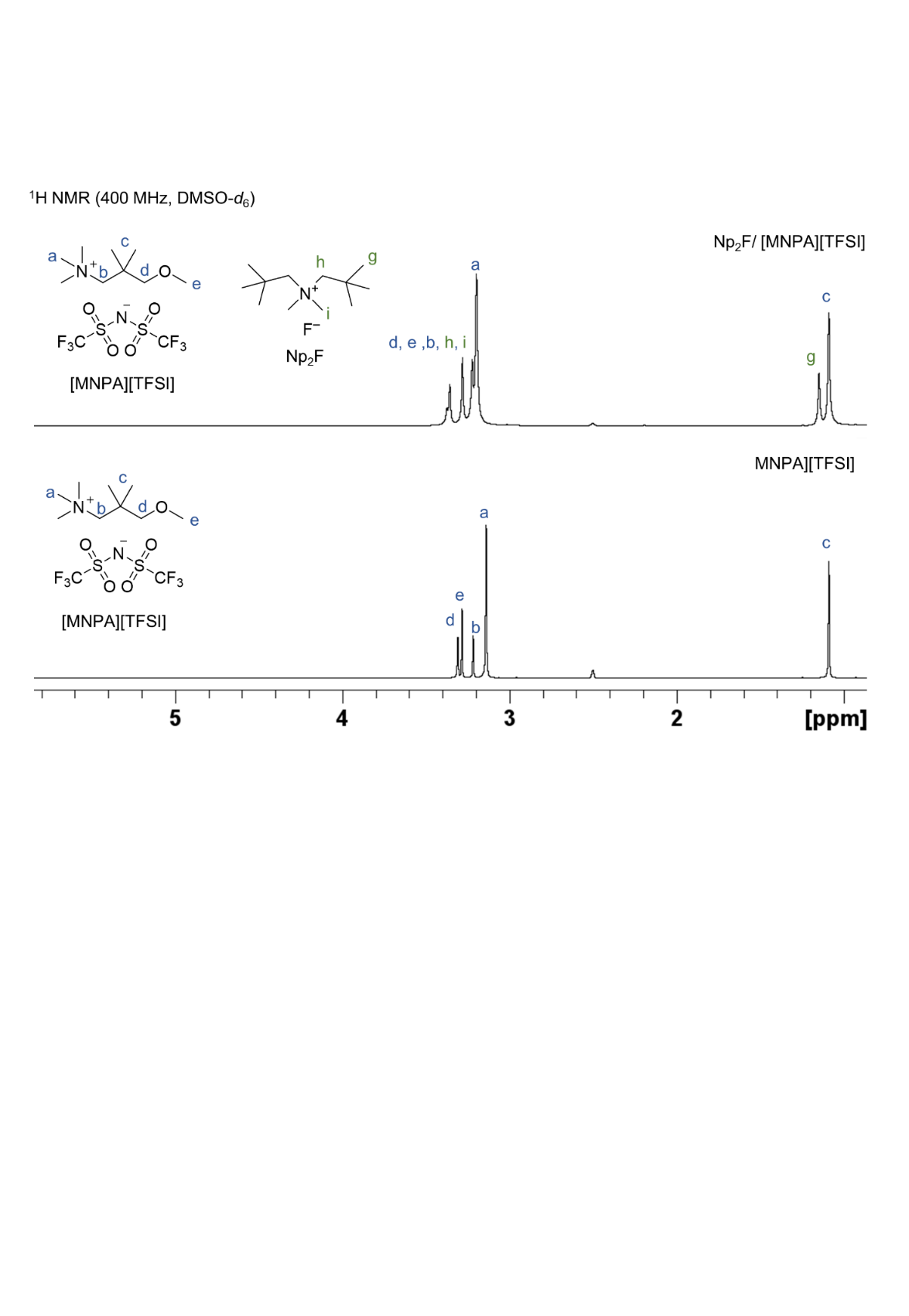


### Figure S4. ^1^HMNR of [MNPA][TFSI] (down) and [MNPA][TFSI]/Np_2_F (up) at room temperature.


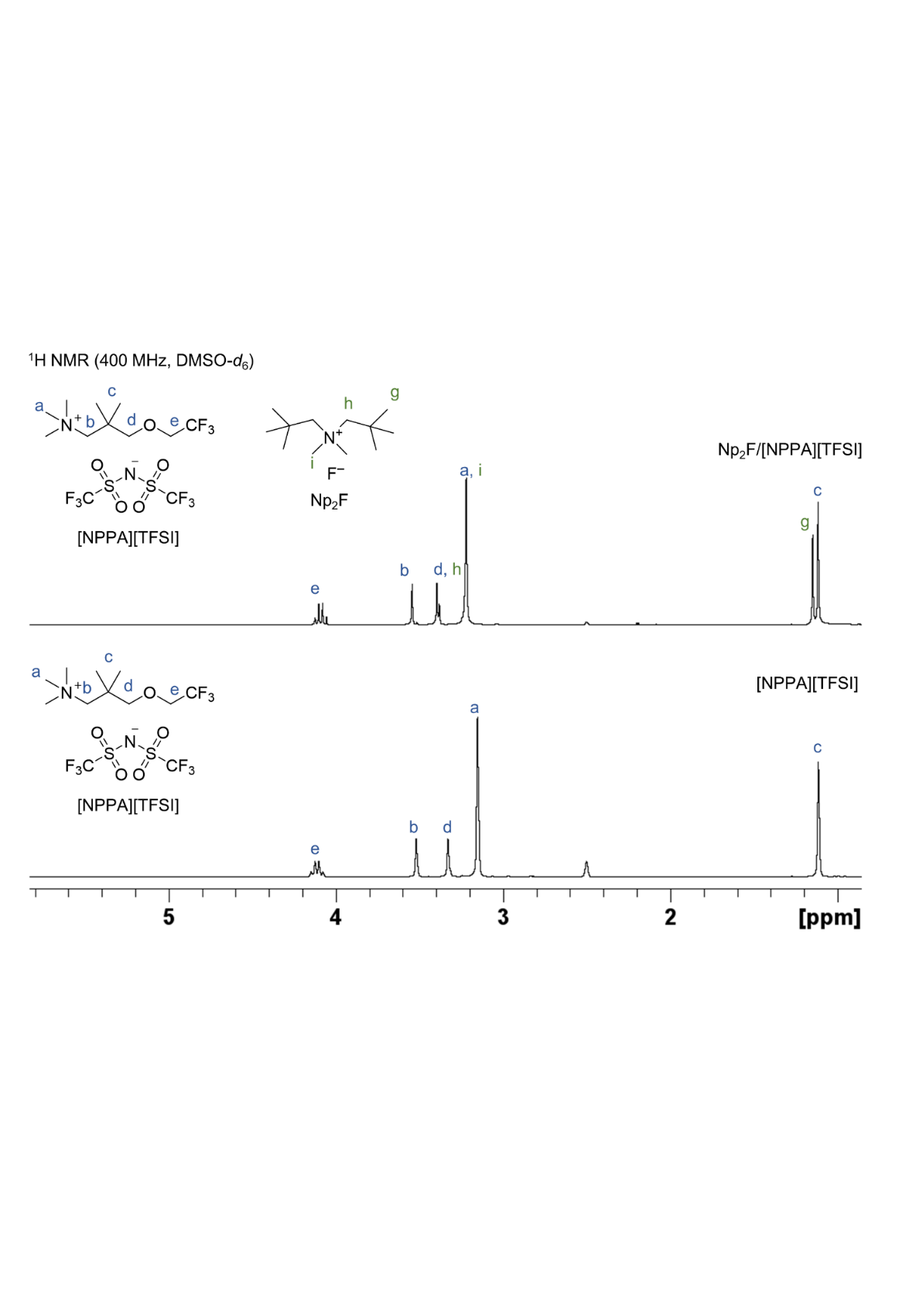


### Figure S5. 1HMNR of [NPPA][TFSI] (down) and [NPPA][TFSI]/Np2F (up) at room temperature.


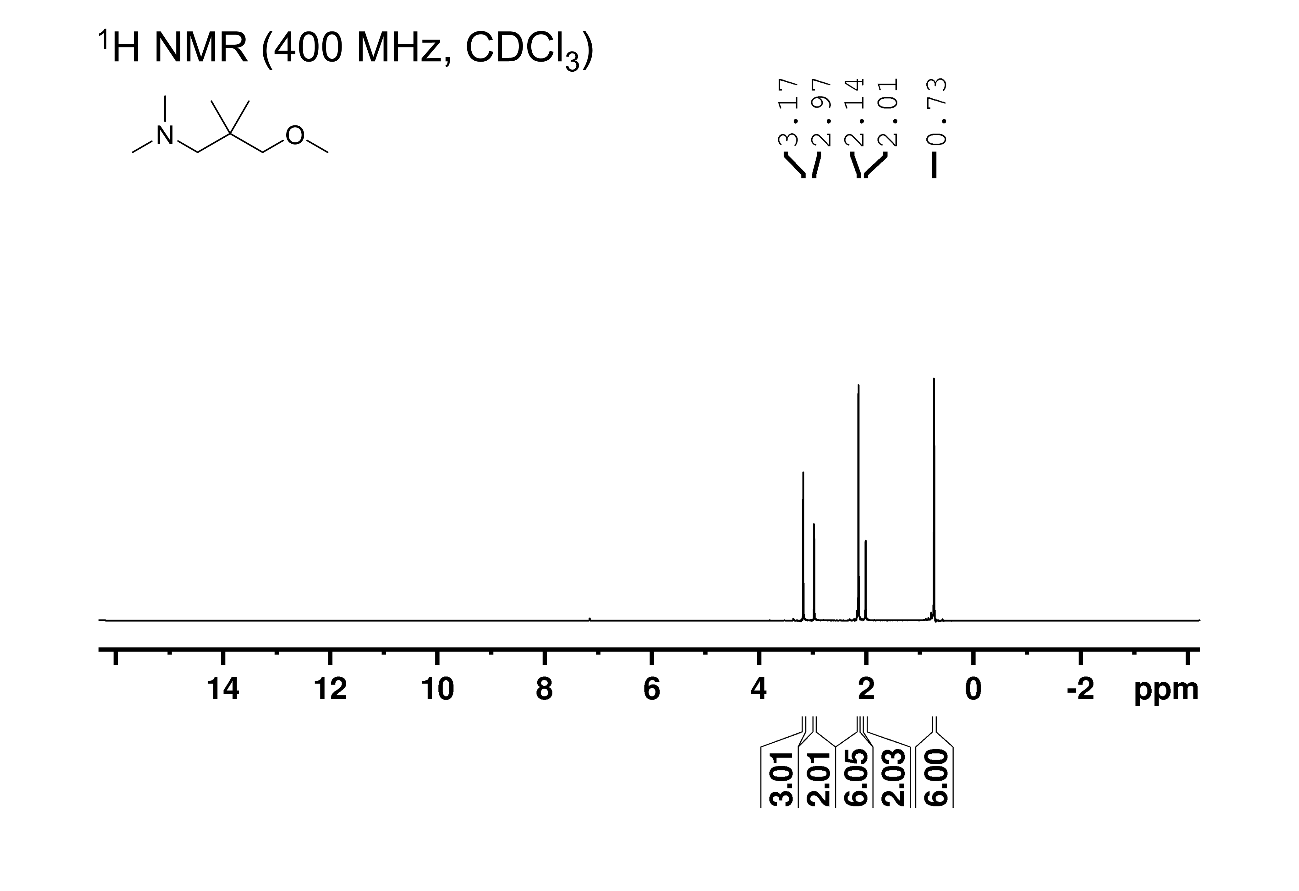


### Figure S6. ^1^H NMR of 3-methoxy-*N*,*N*,2,2-tetramethylpropan-1-amine.


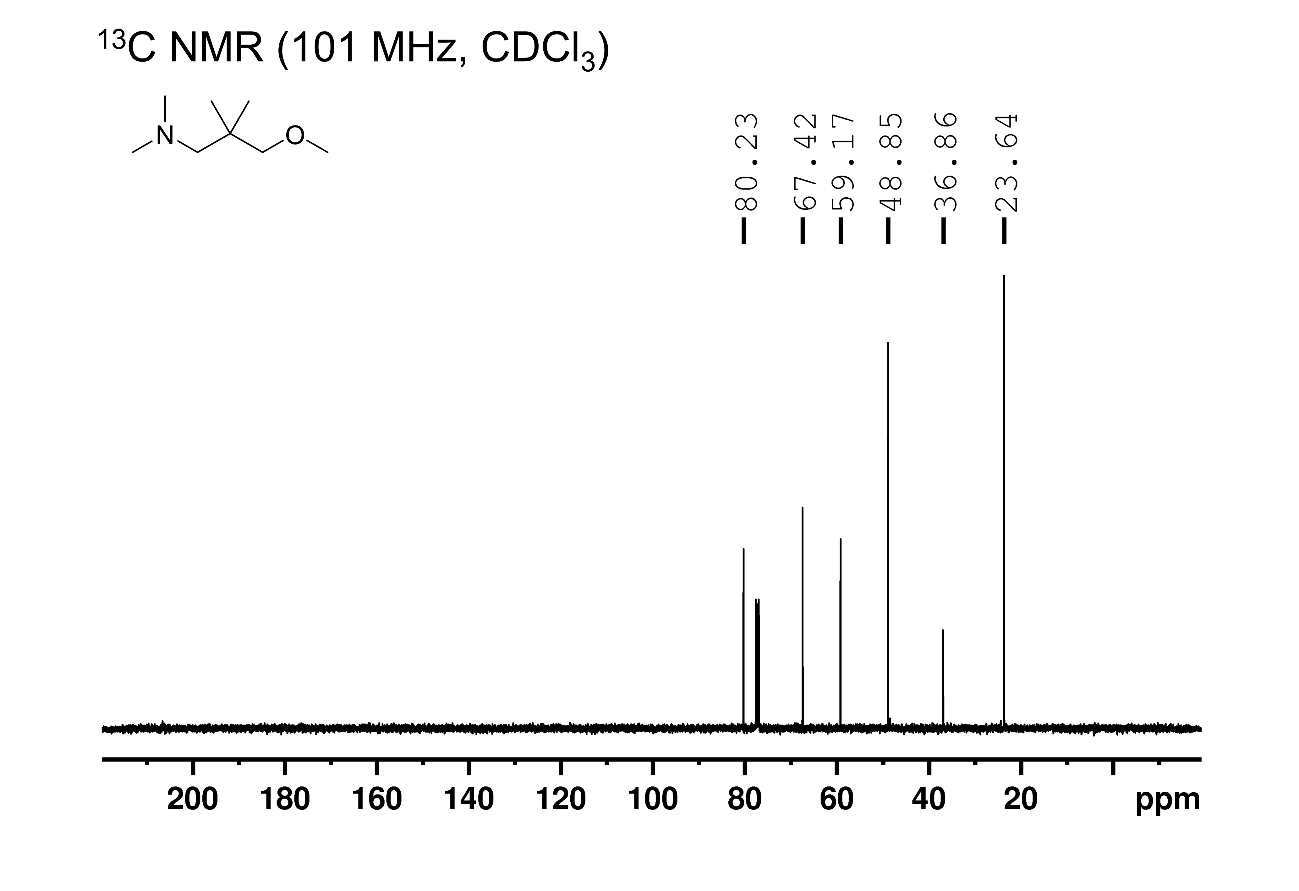


### Figure S7. ^13^CNMR of 3-methoxy-*N*,*N*,2,2-tetramethylpropan-1-amine.


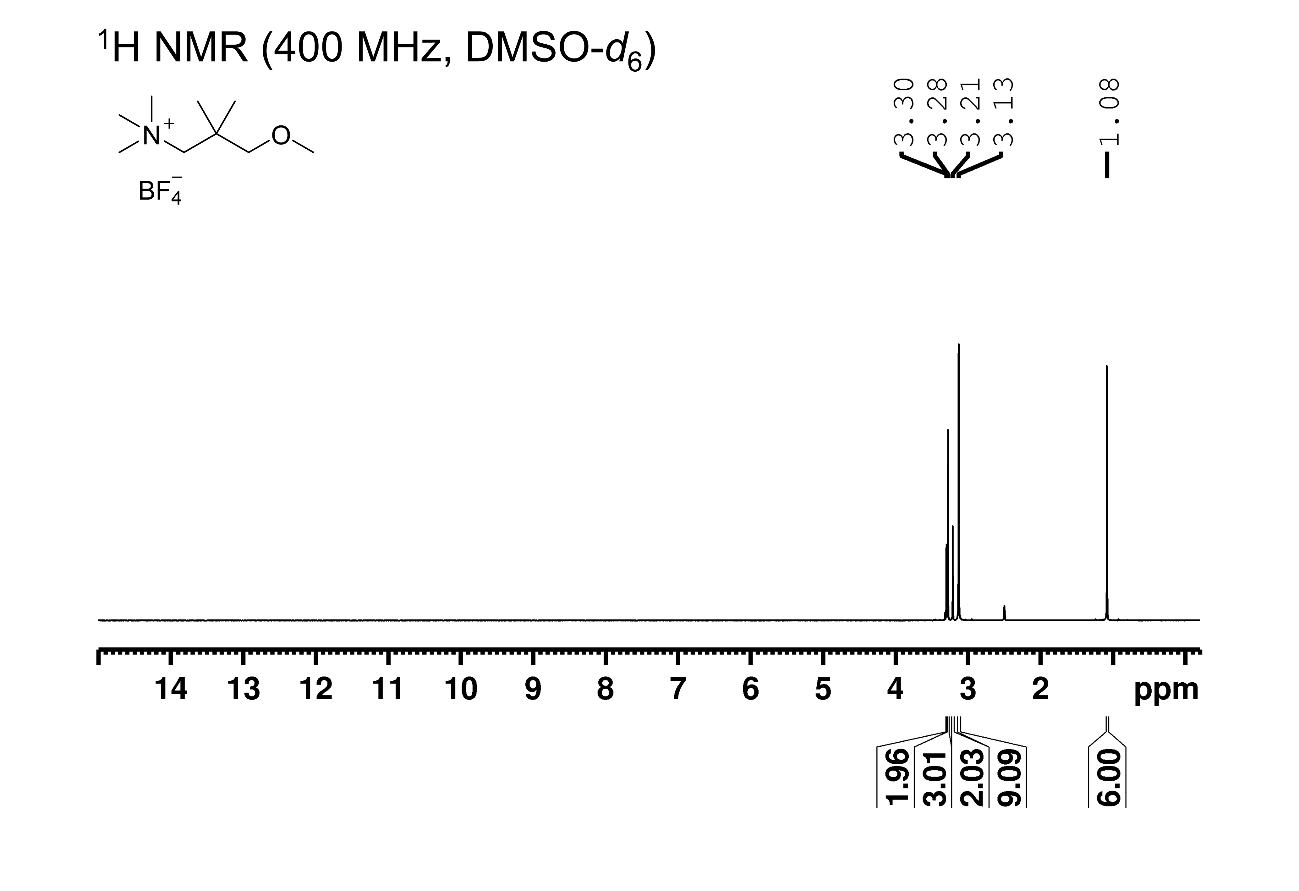


### Figure S8. ^1^H NMR of [MNPA][BF_4_].


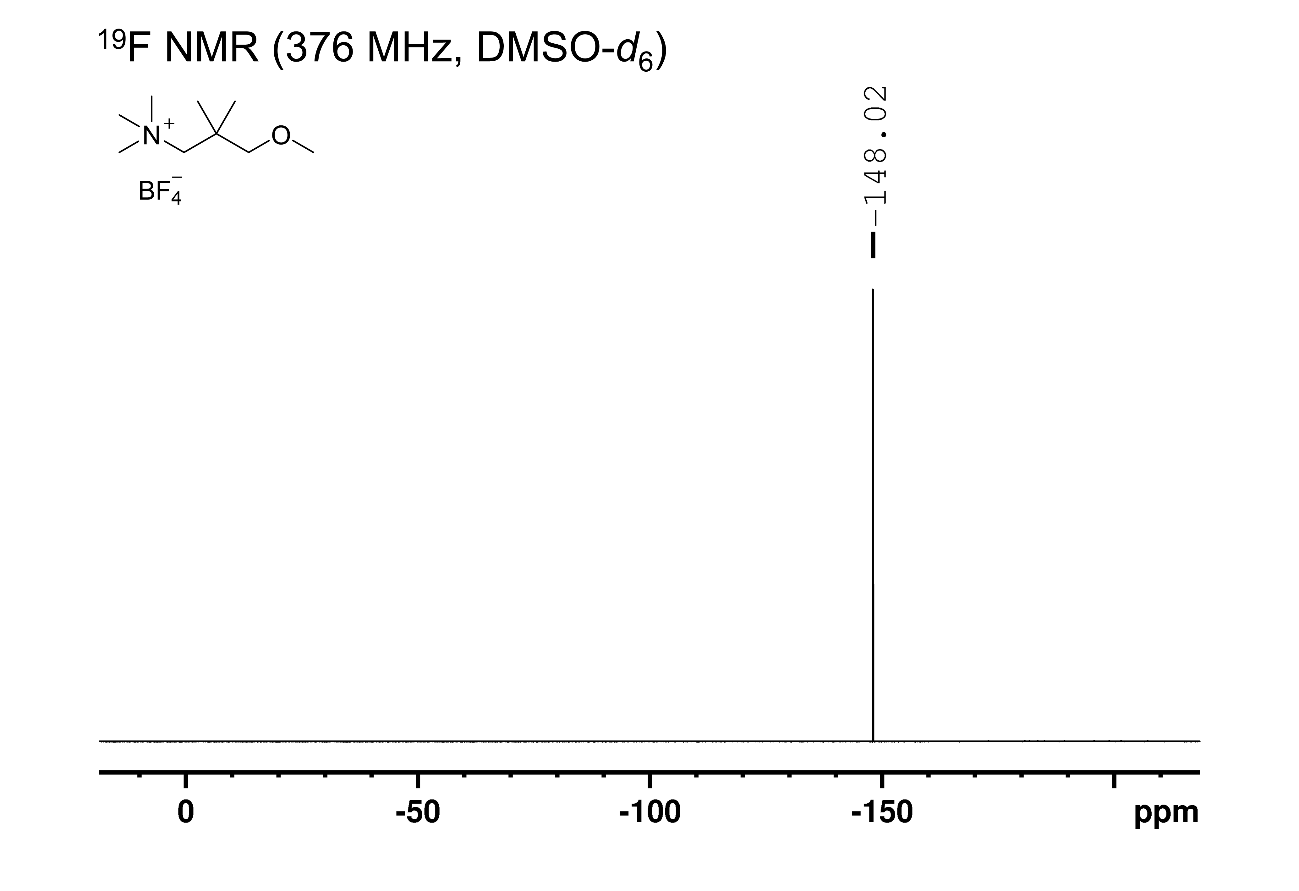


### Figure S9. ^19^F NMR of [MNPA][BF_4_].


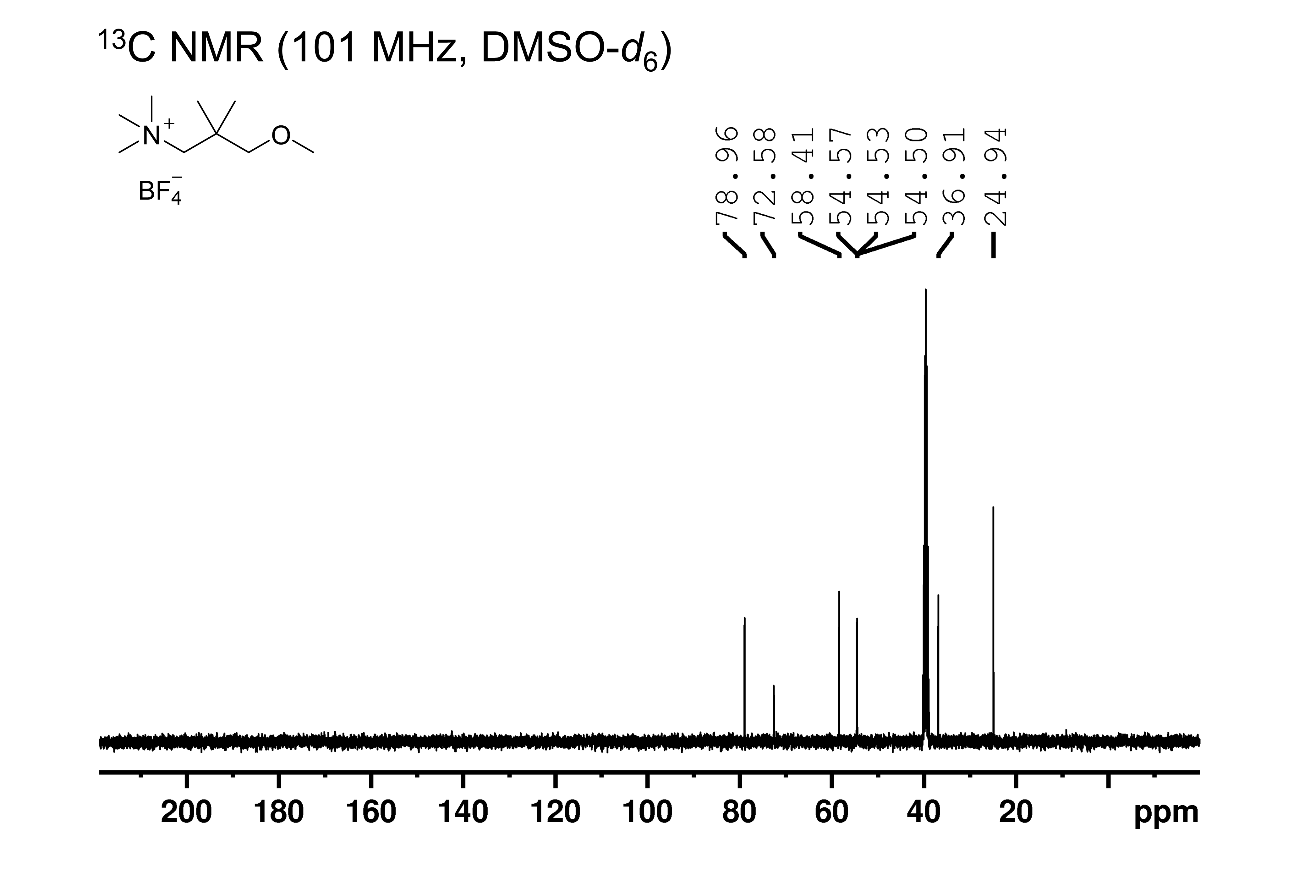


### Figure S10. ^13^C NMR of [MNPA][BF_4_].


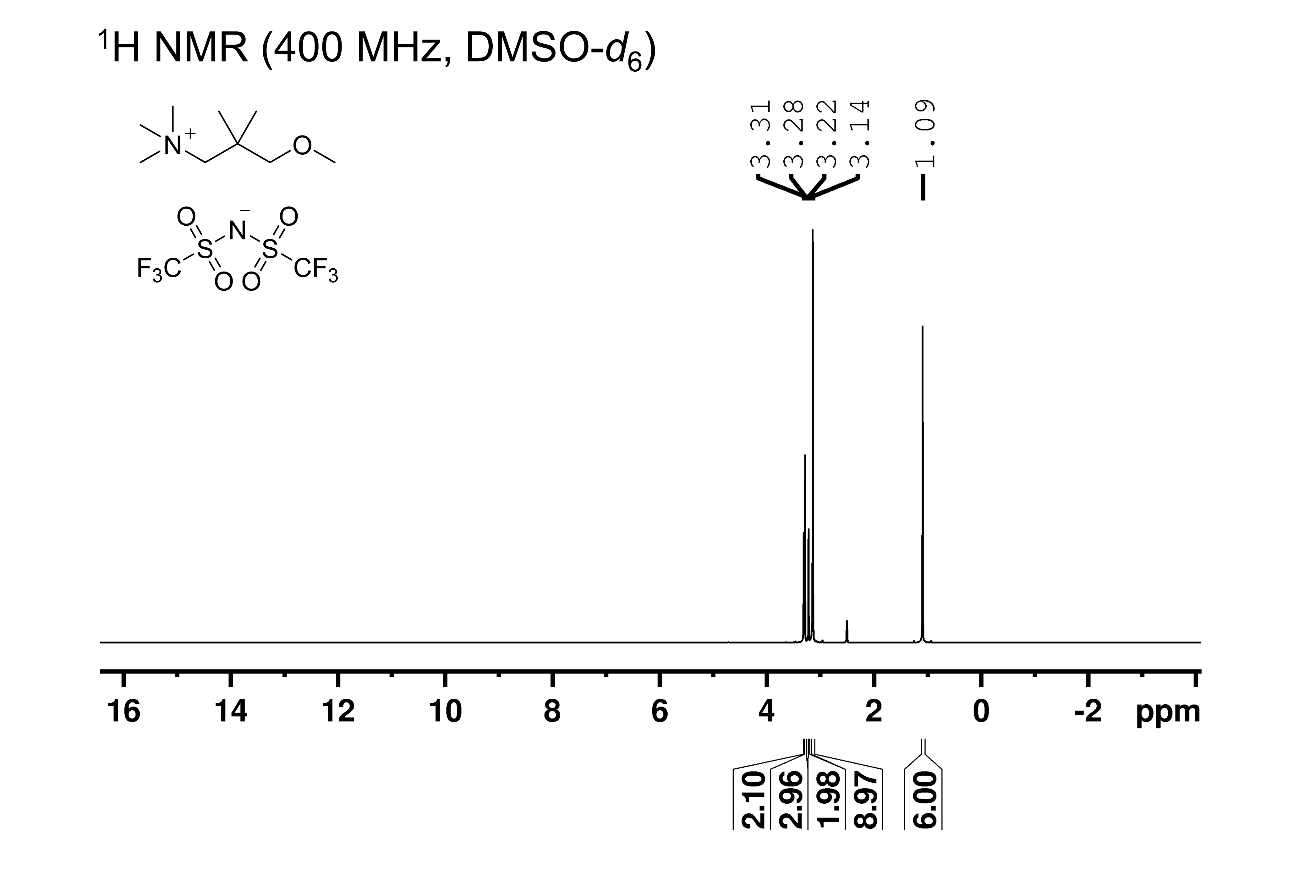


### Figure S11. ^1^H NMR of [MNPA][TFSI].


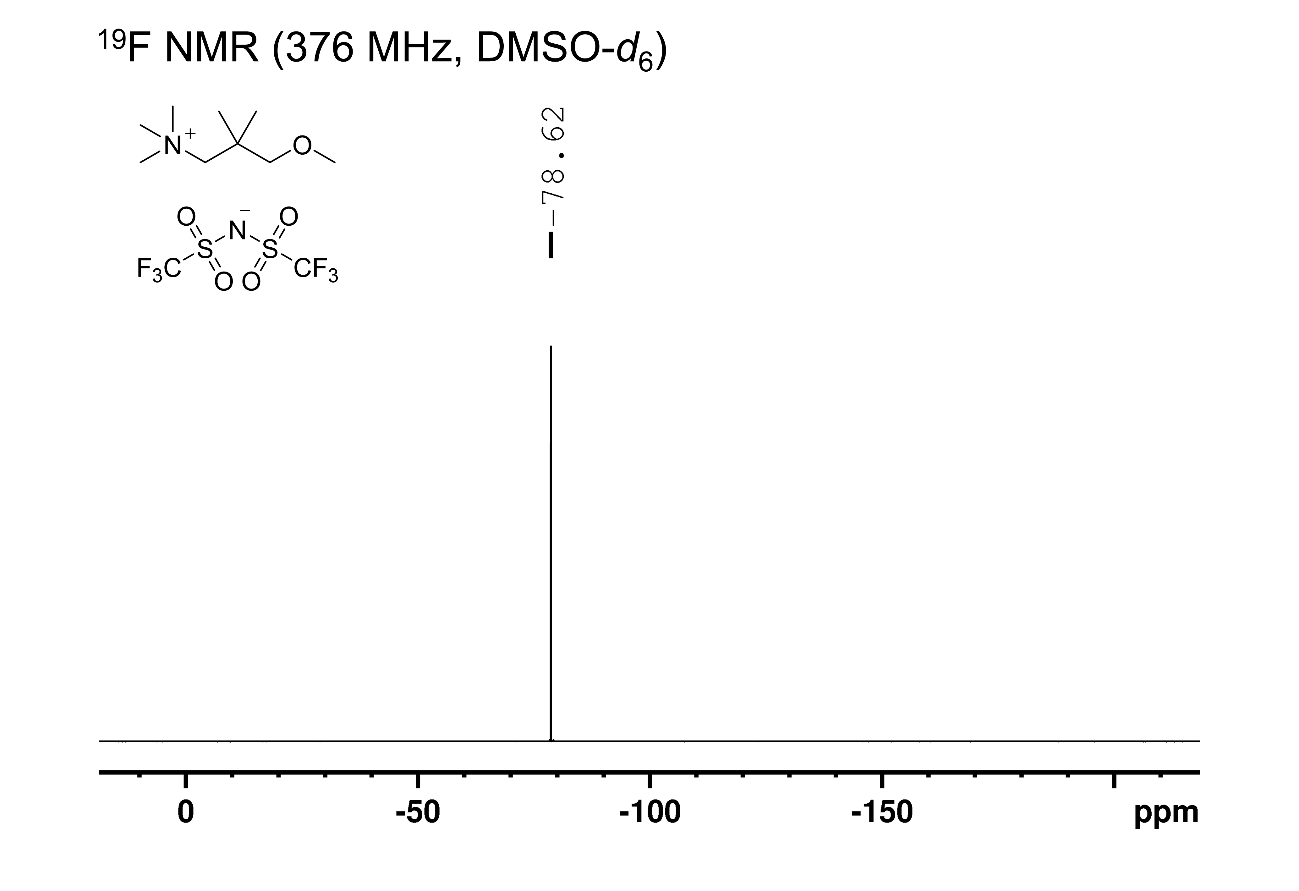


Figure S12. ^19^F NMR of [MNPA][TFSI].


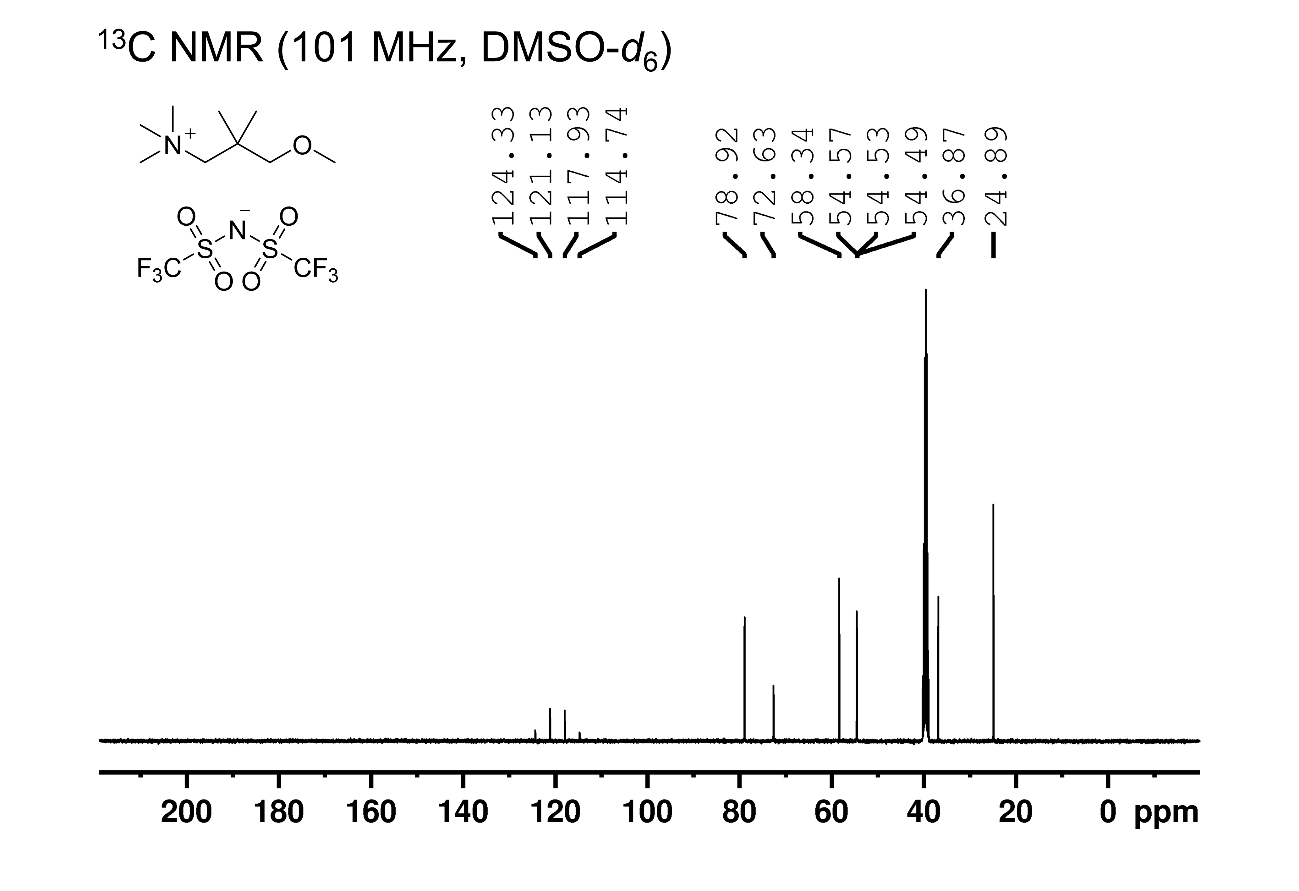


Figure S13. ^13^C NMR of [MNPA][TFSI].


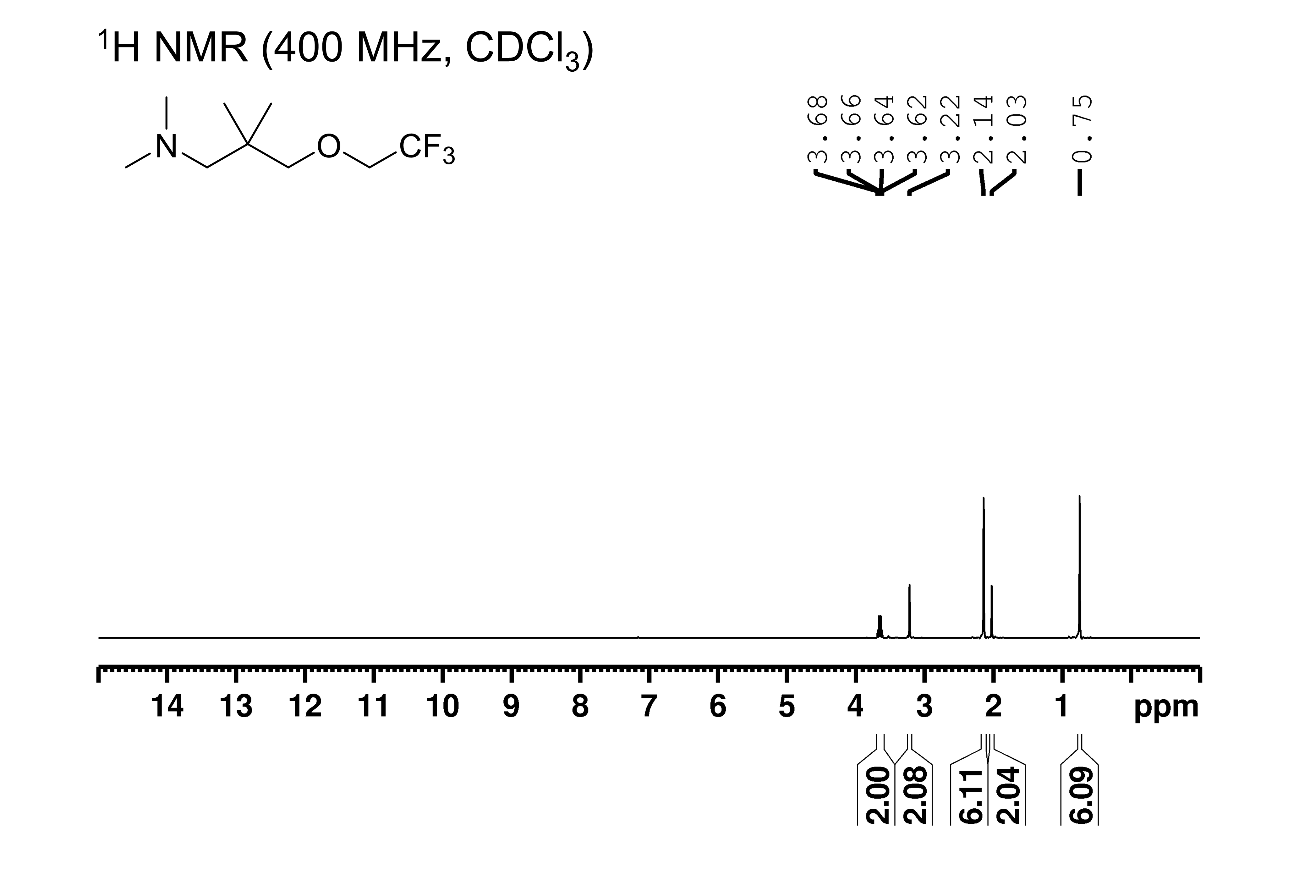


Figure S14. ^1^H NMR of *N*,*N*,2,2-tetramethyl-3-(2,2,2-trifluoroethoxy)propan-1-amine.


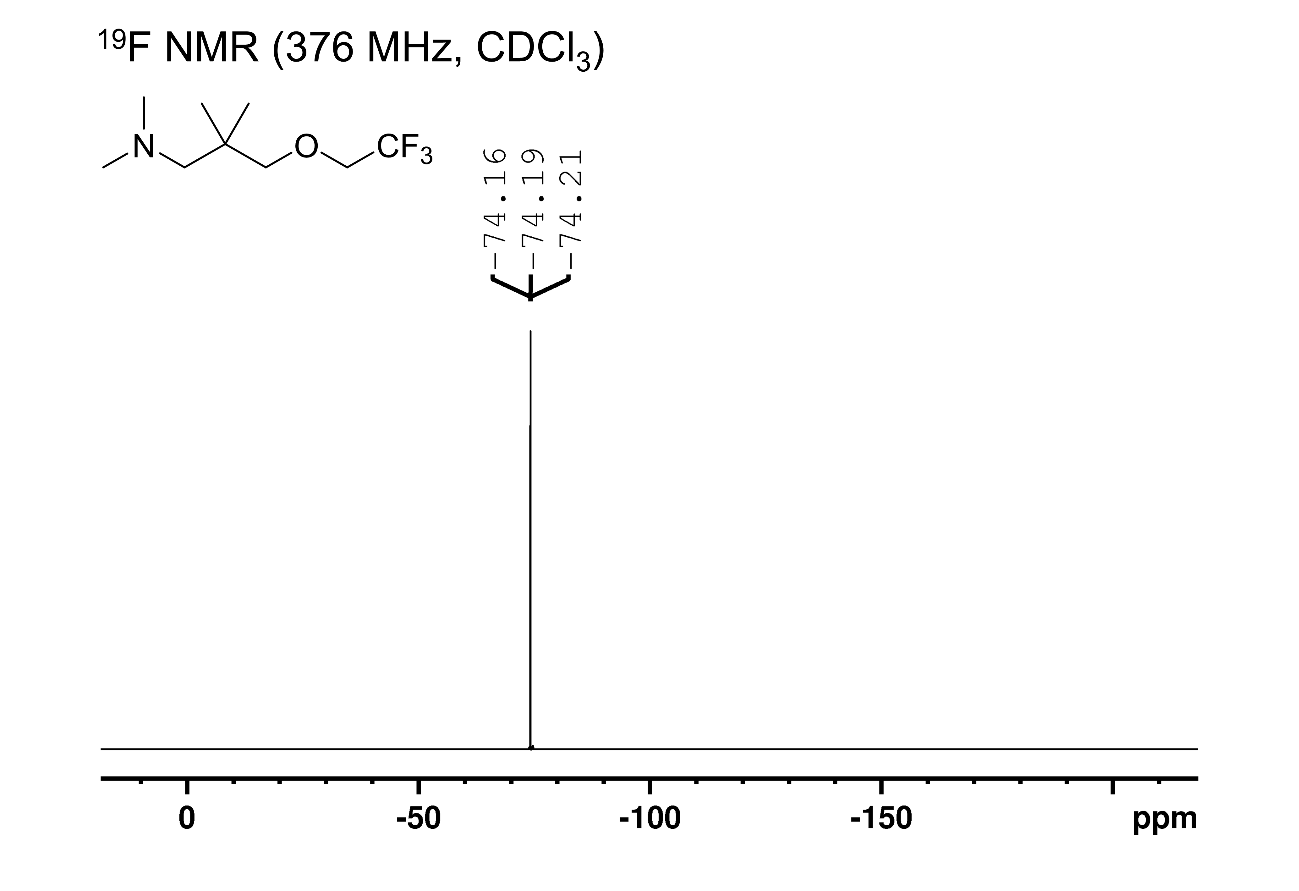


Figure S15. ^19^F NMR of *N*,*N*,2,2-tetramethyl-3-(2,2,2-trifluoroethoxy)propan-1-amine.


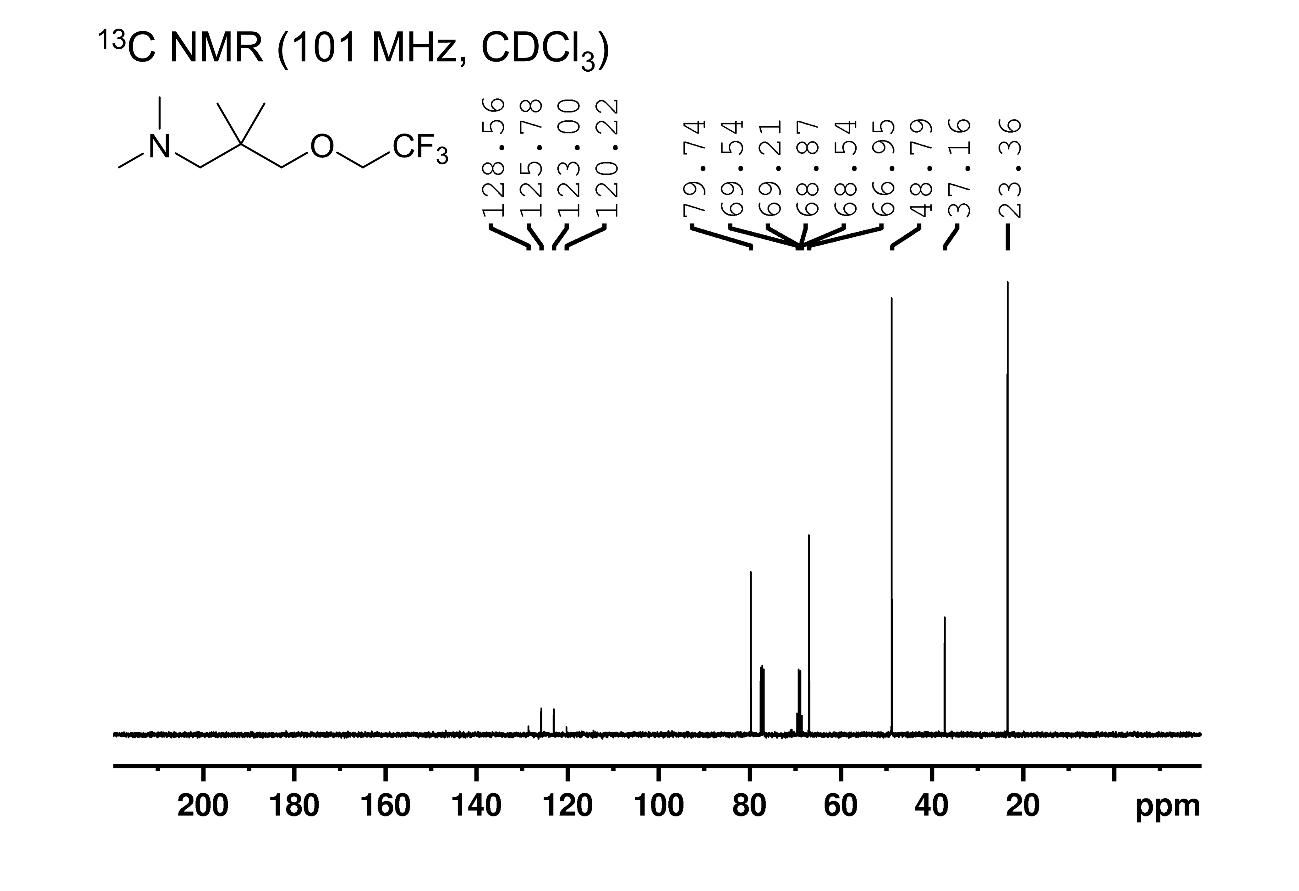


Figure S16. ^13^C NMR of *N*,*N*,2,2-tetramethyl-3-(2,2,2-trifluoroethoxy)propan-1-amine.


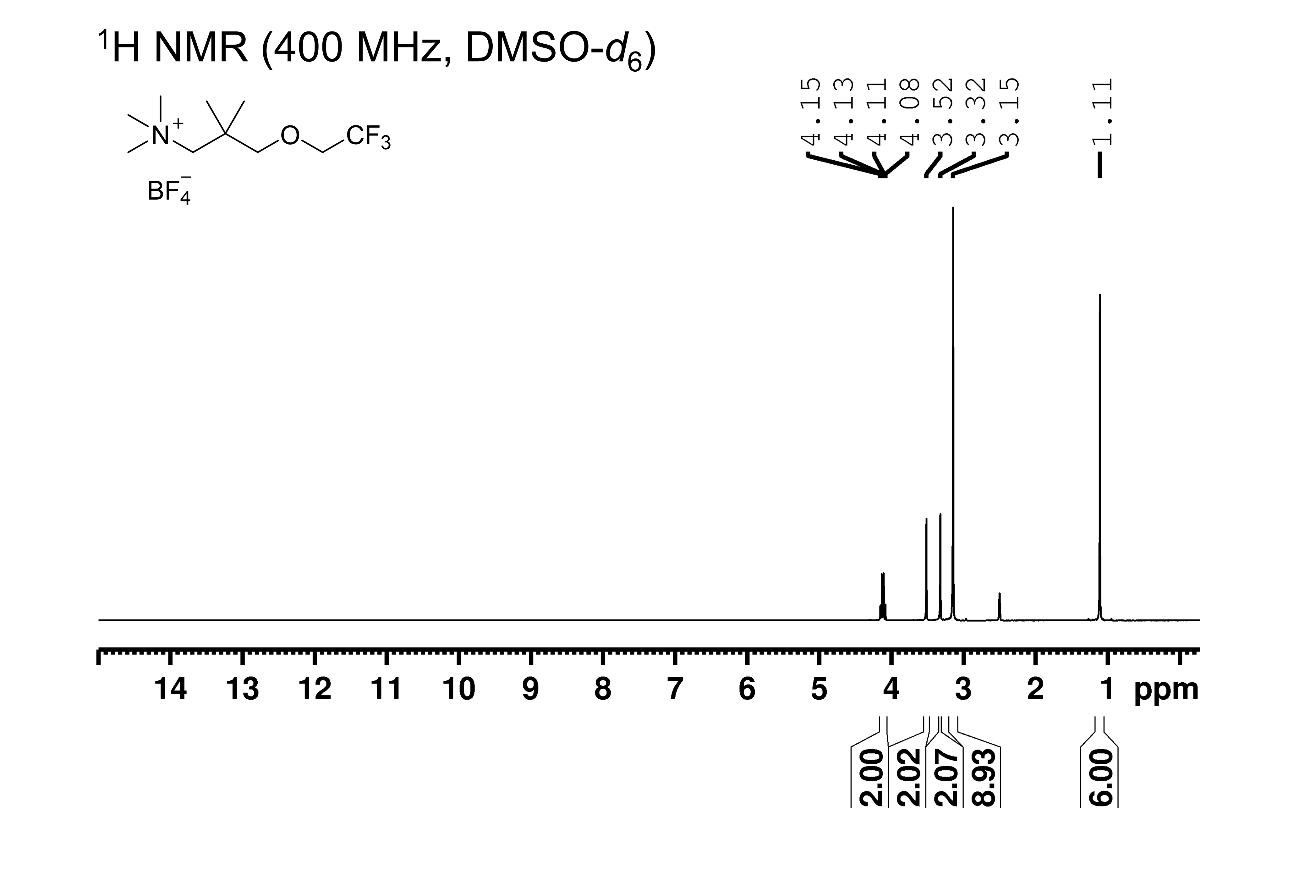


Figure S17. ^1^H NMR of [NPPA][BF_4_].


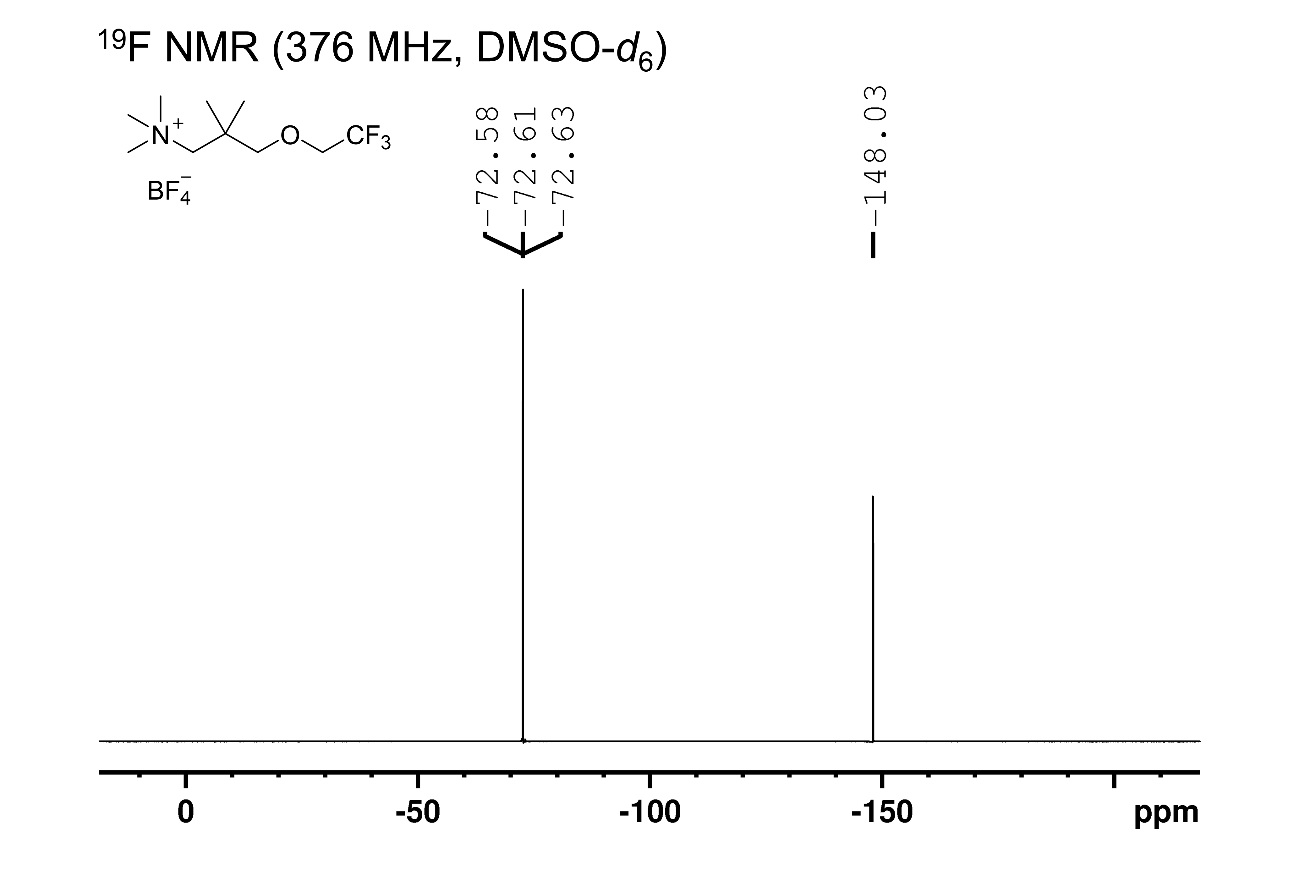


Figure S18. ^19^F NMR of [NPPA][BF_4_].


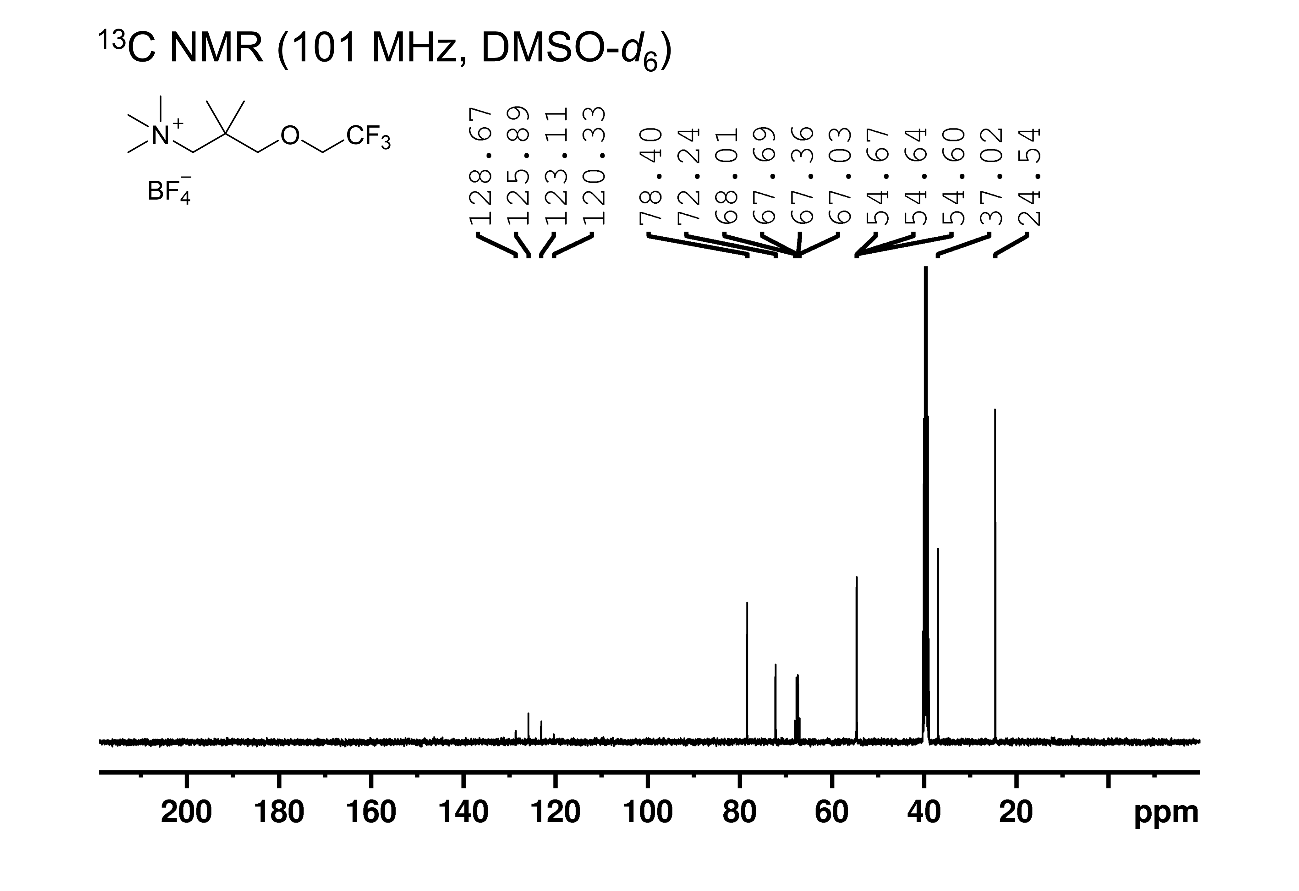


Figure S19. ^13^C NMR of [NPPA][BF_4_].


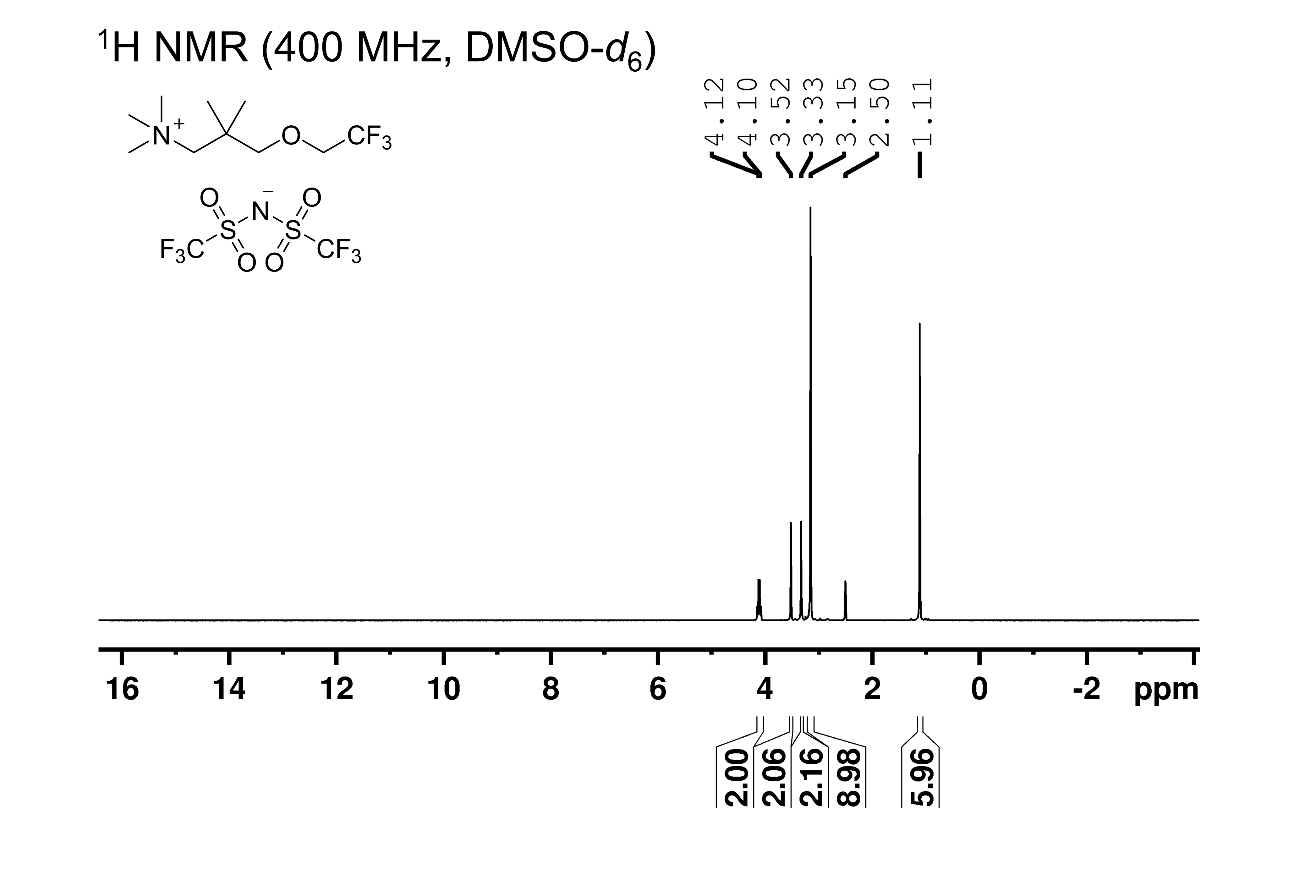


Figure S20. ^1^H NMR of [NPPA][TFSI].


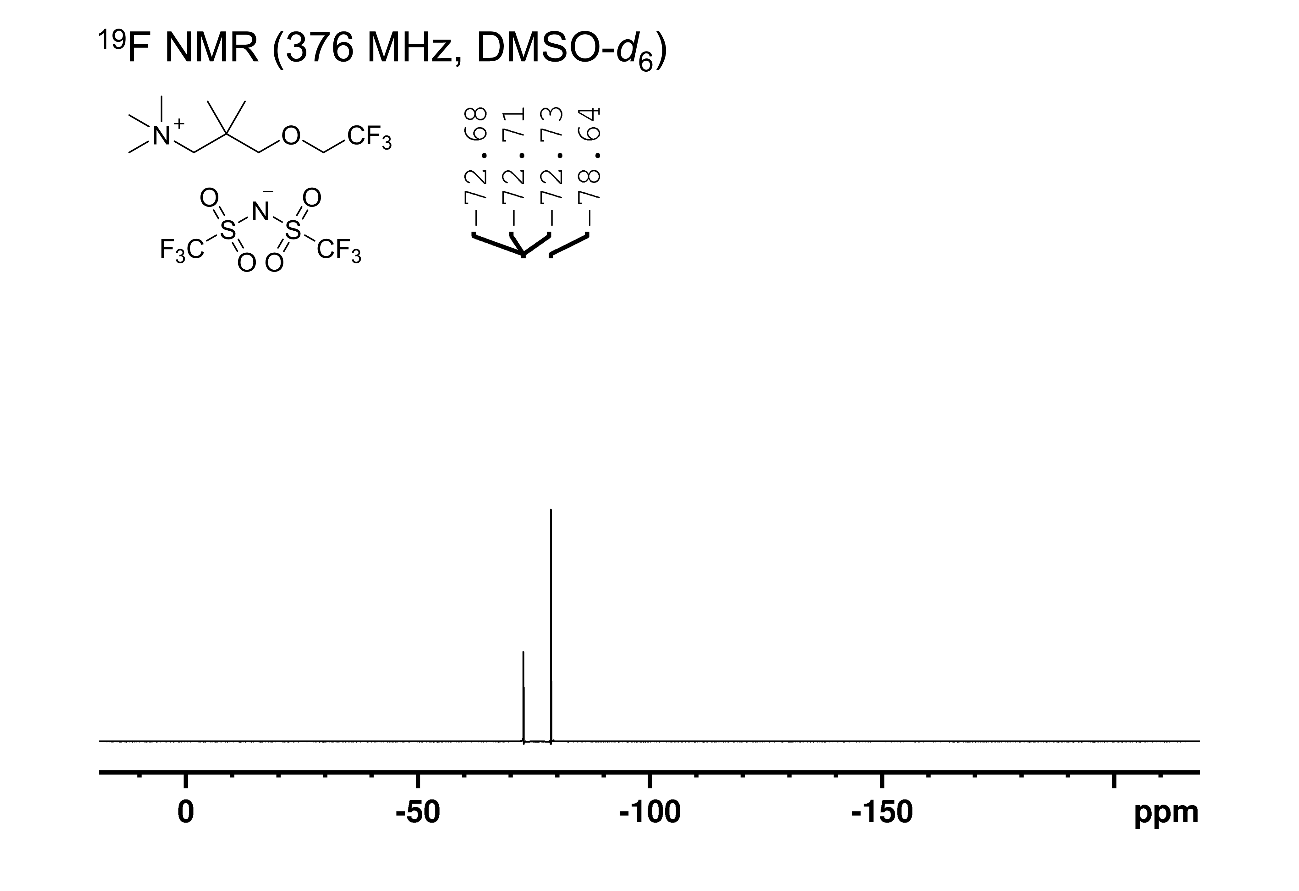


Figure S21. ^19^F NMR of [NPPA][TFSI].


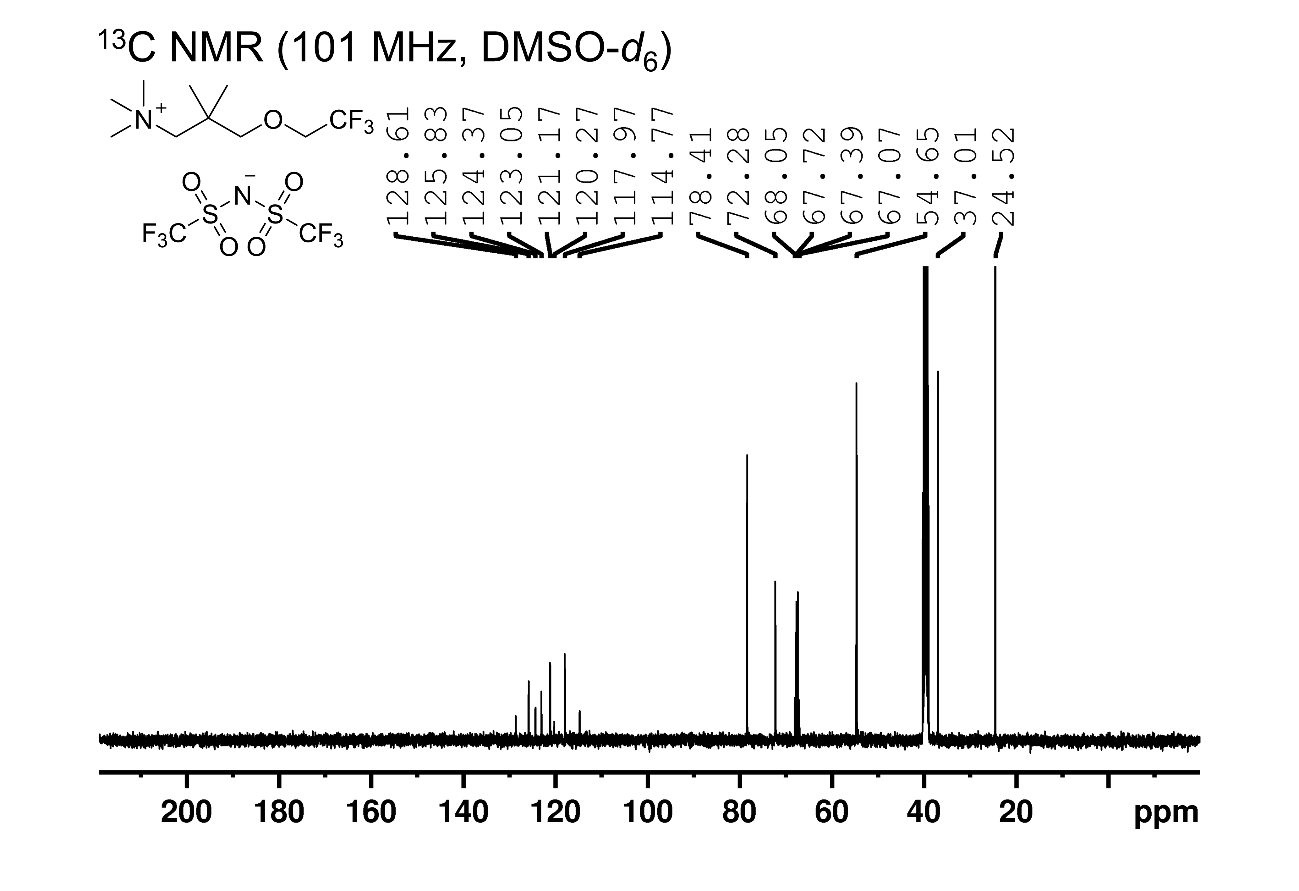


Figure S22. ^13^C NMR of [NPPA][TFSI].


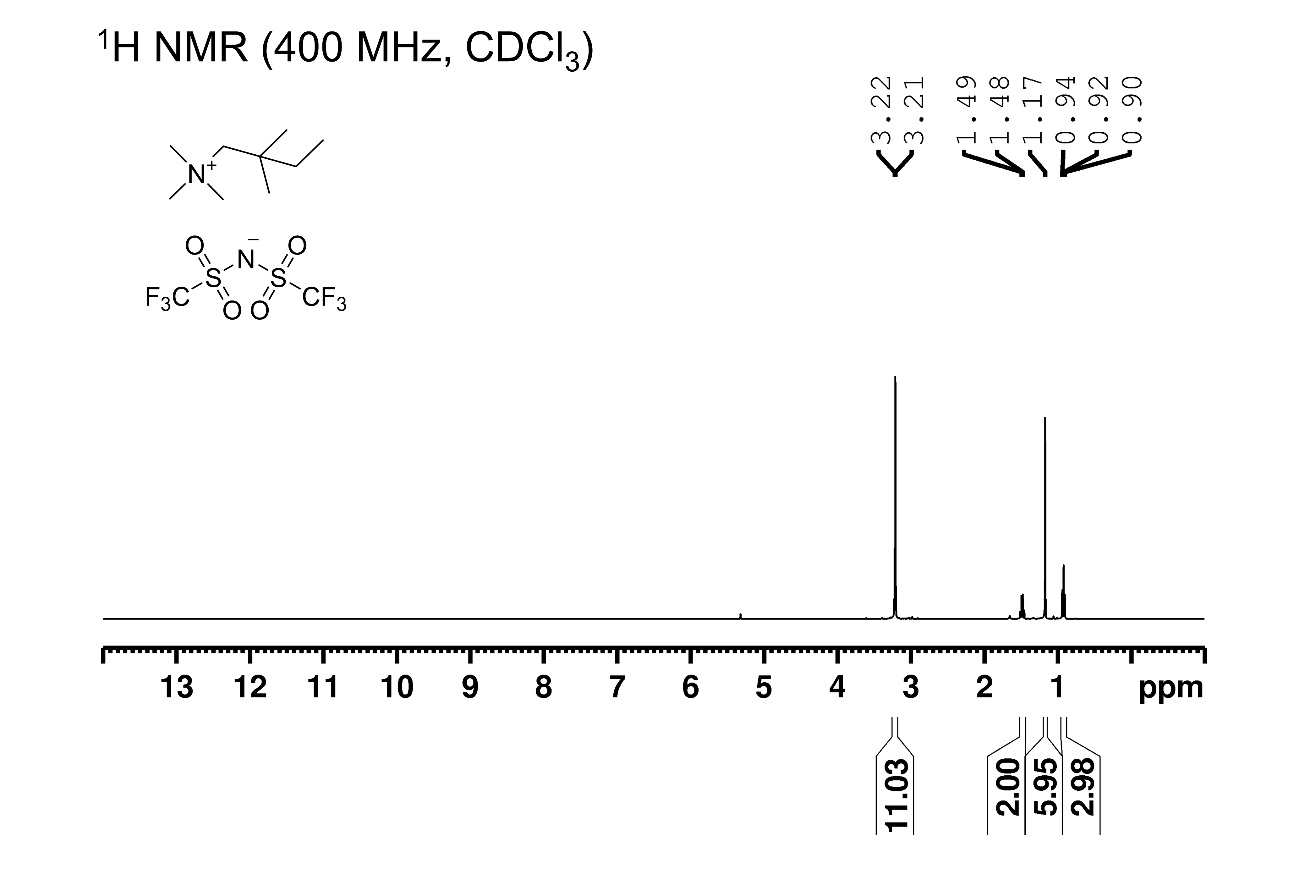


Figure S23. ^1^H NMR of [MeDMB][TFSI].


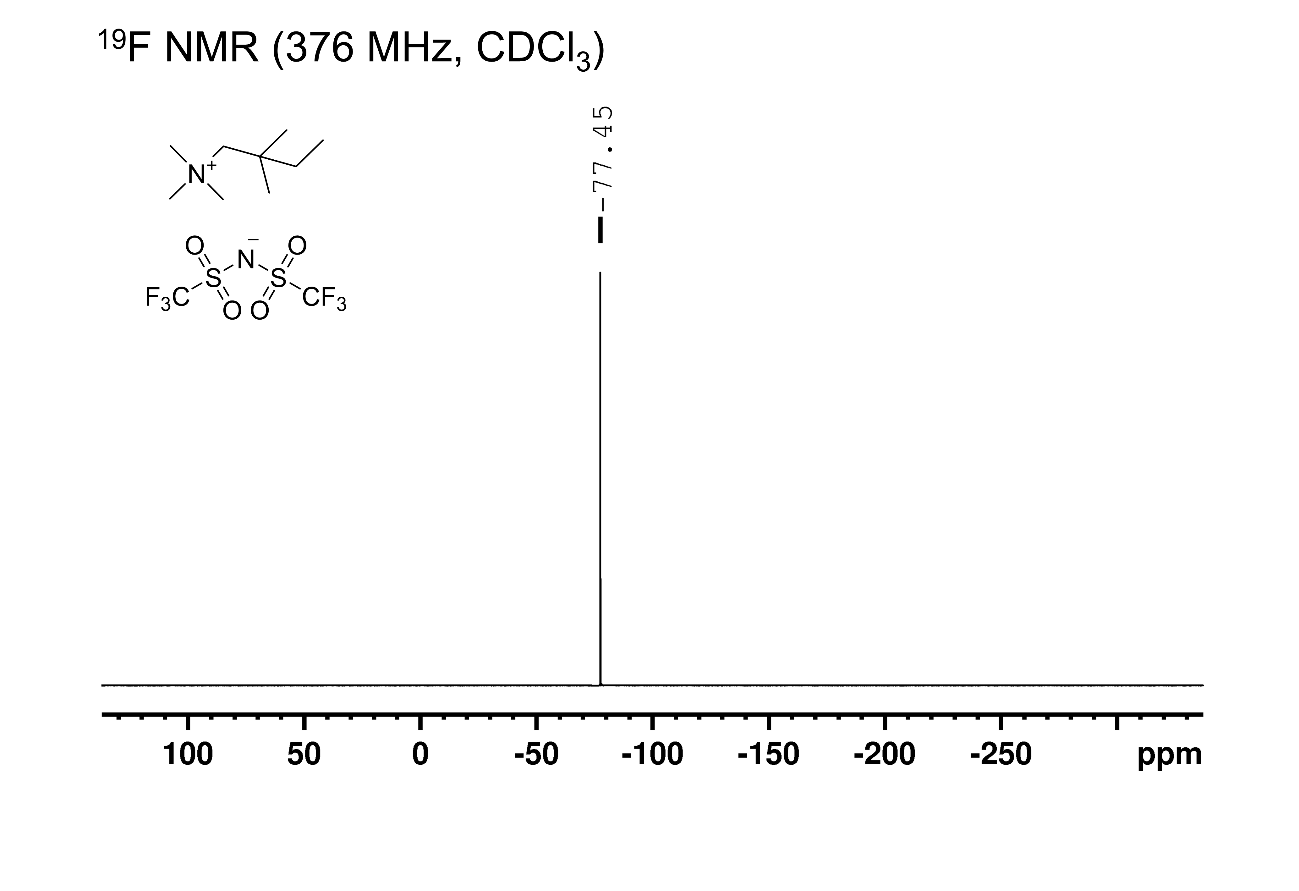


Figure S24. ^19^F NMR of [MeDMB][TFSI].


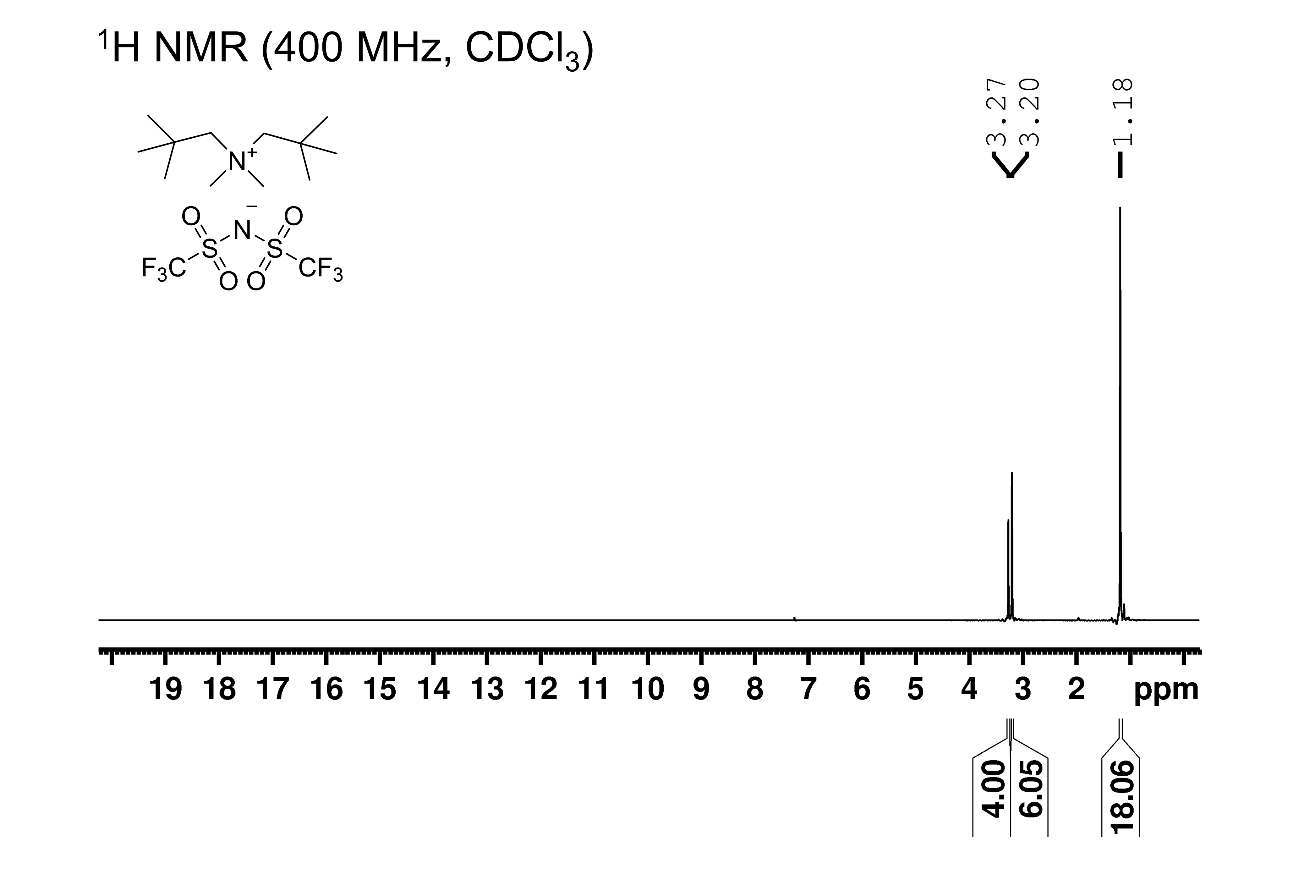


Figure S25. ^1^H NMR of [Np_2_][TFSI].


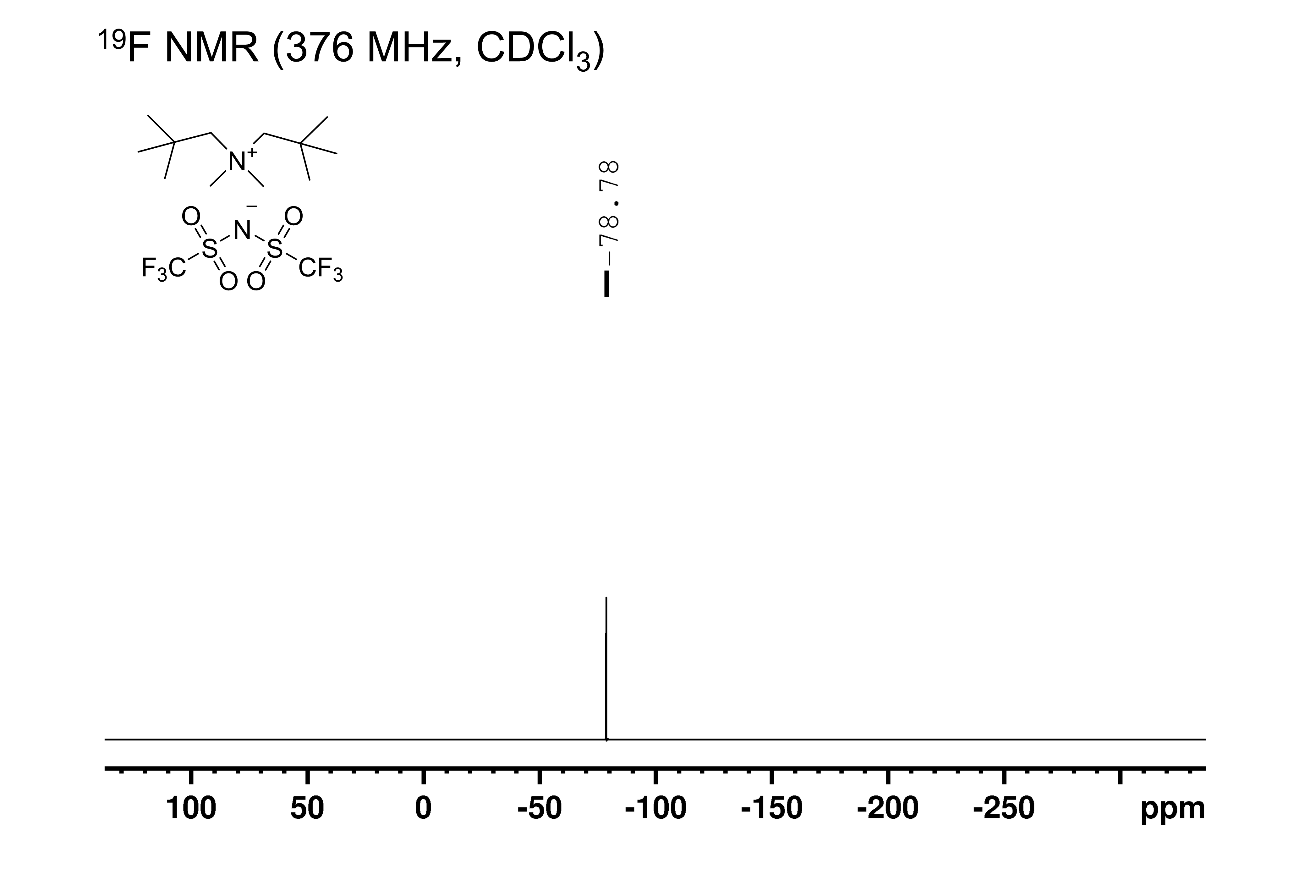


Figure S26. ^19^F NMR of [Np_2_][TFSI].


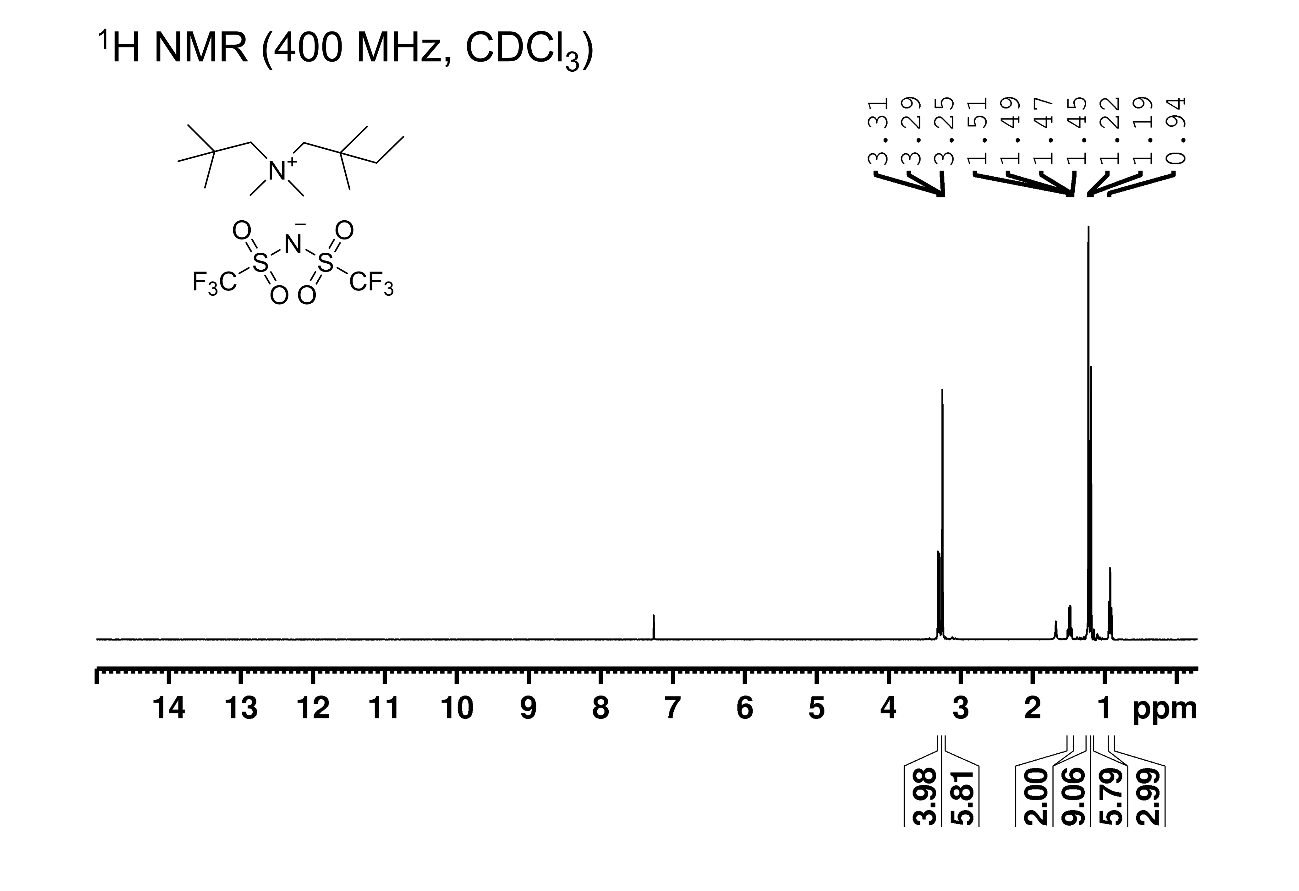


Figure S27. ^1^H NMR of [NpDMB][TFSI].


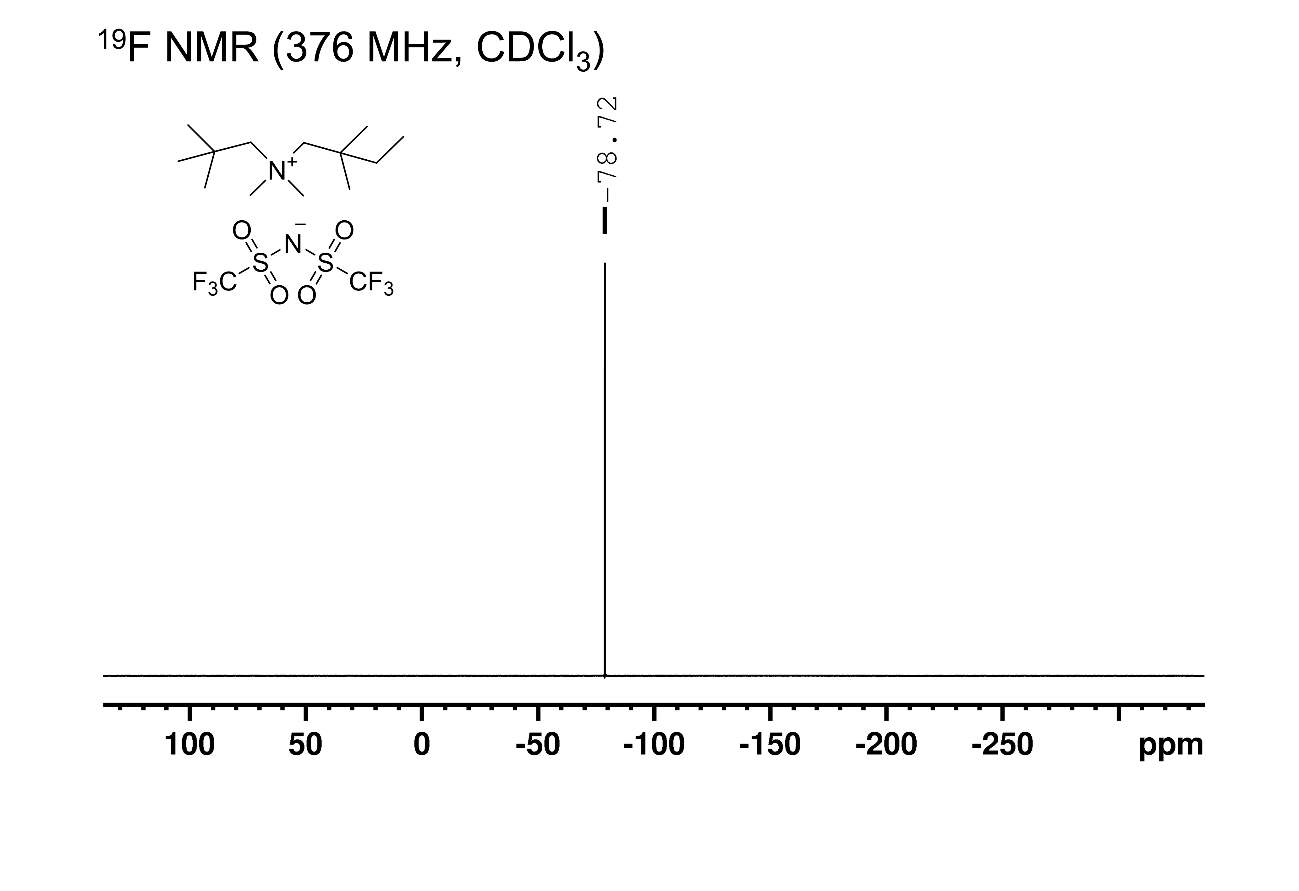


Figure S28. ^19^F NMR of [NpDMB][TFSI].


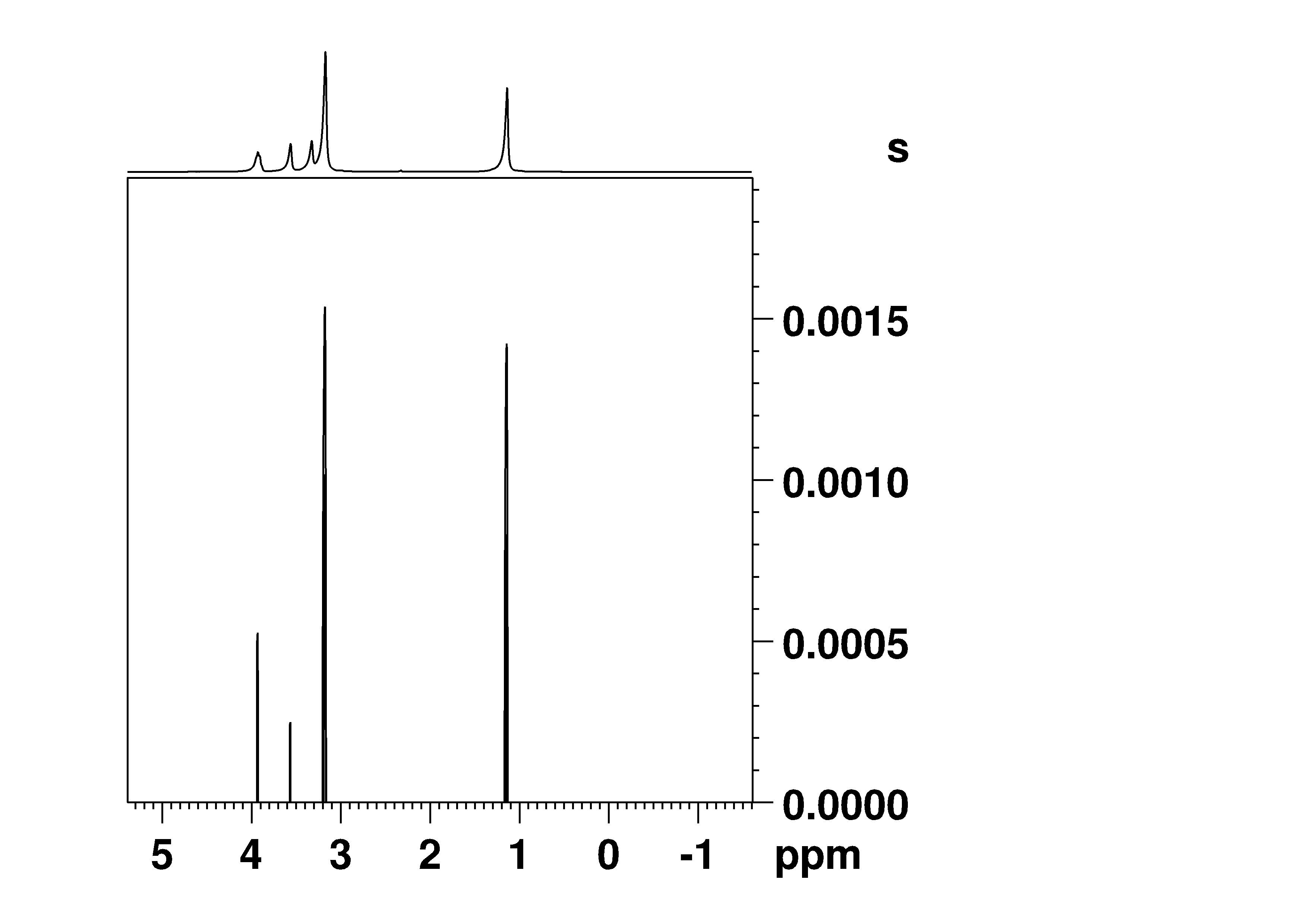


Figure S29. ^1^H DOSY NMR of [NPPA][TFSI] at 298 K.


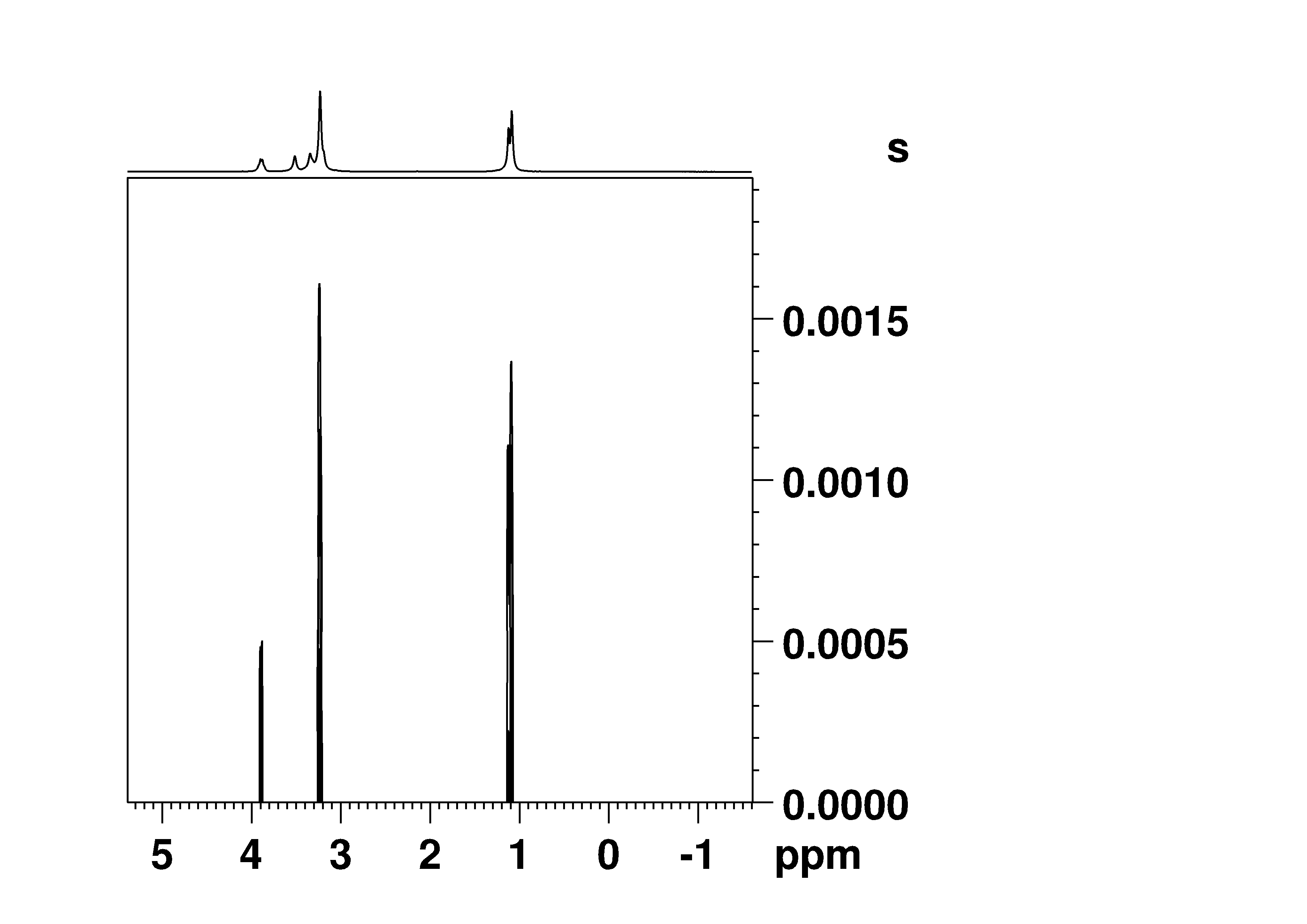


Figure S30. ^1^H DOSY NMR of [NPPA][TFSI]/Np_2_F at 298 K.


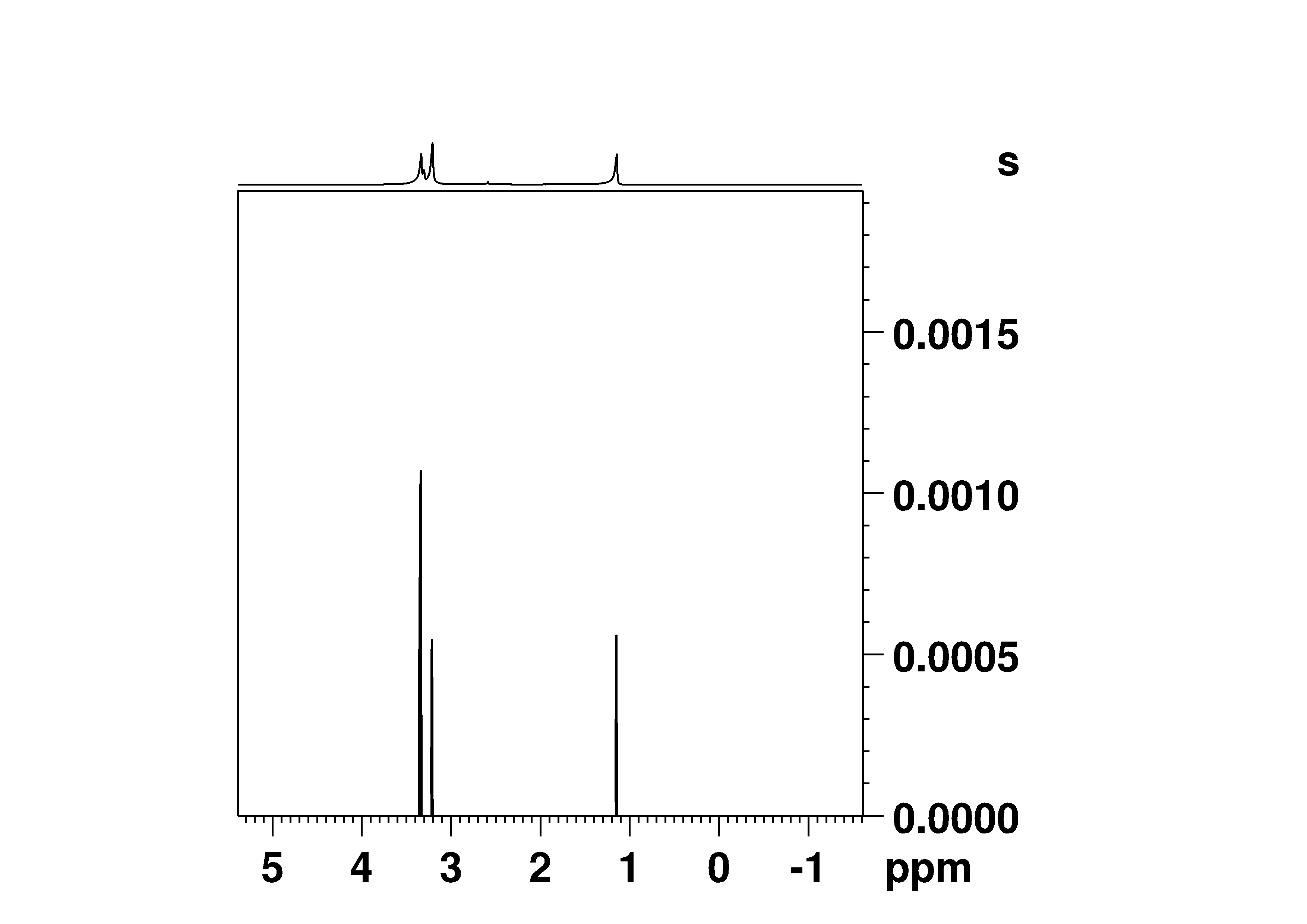


Figure S31. ^1^H DOSY NMR of [MNPA][TFSI] at 298 K.


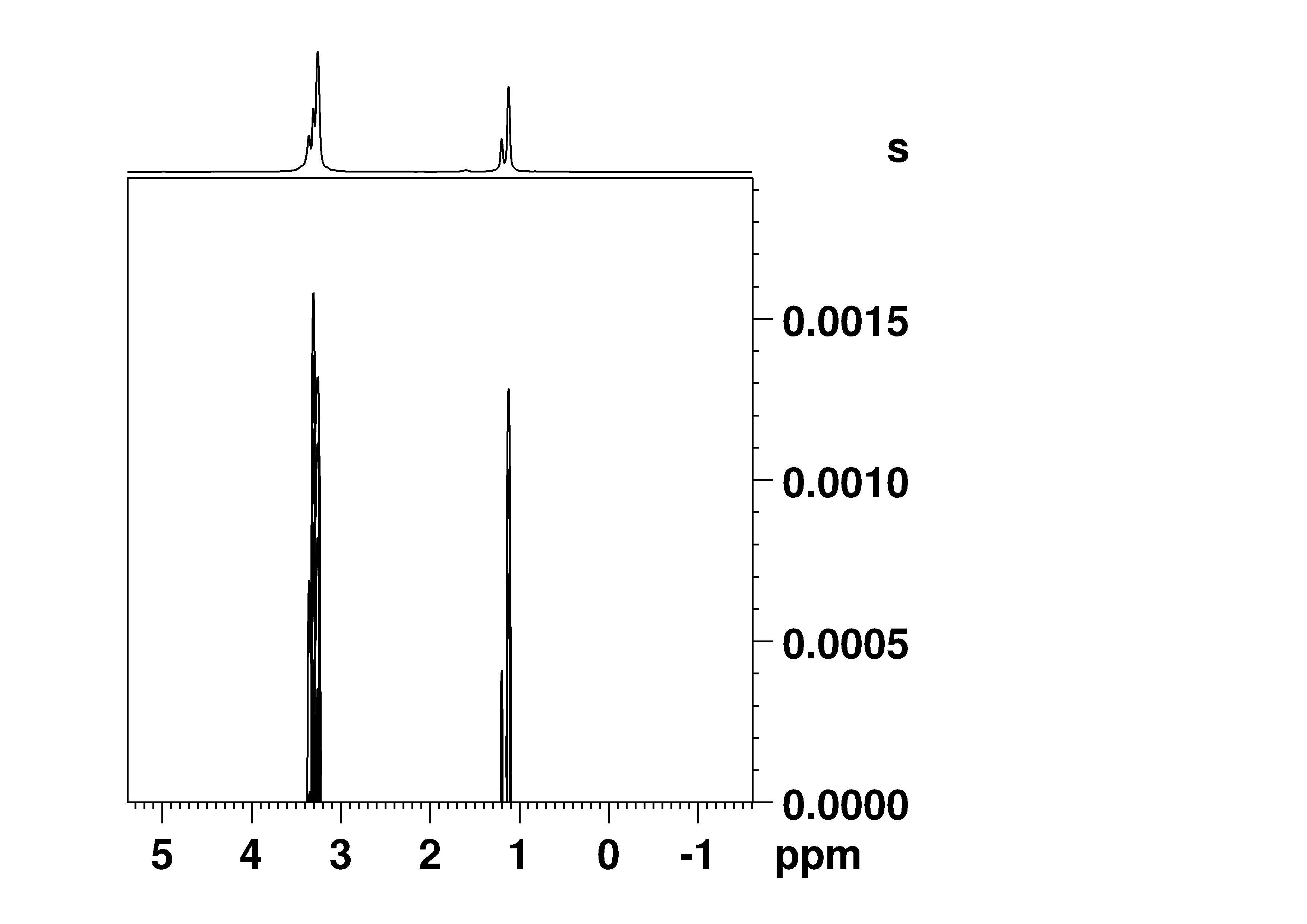


Figure S32. ^1^H DOSY NMR of [MNPA][TFSI]/Np_2_F at 298 K.

.
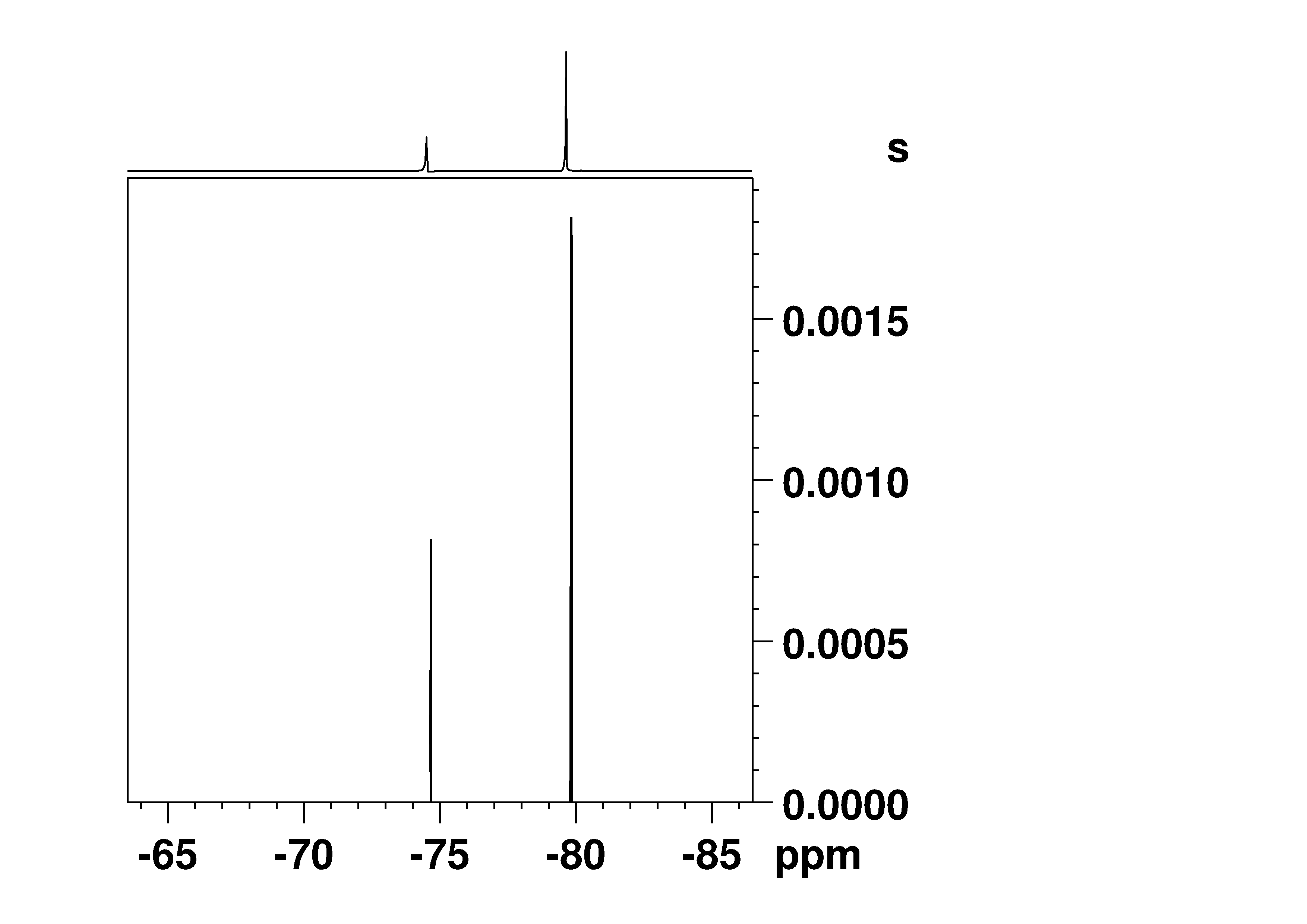


Figure S33. ^19^F DOSY NMR of [NPPA][TFSI] at 298 K.


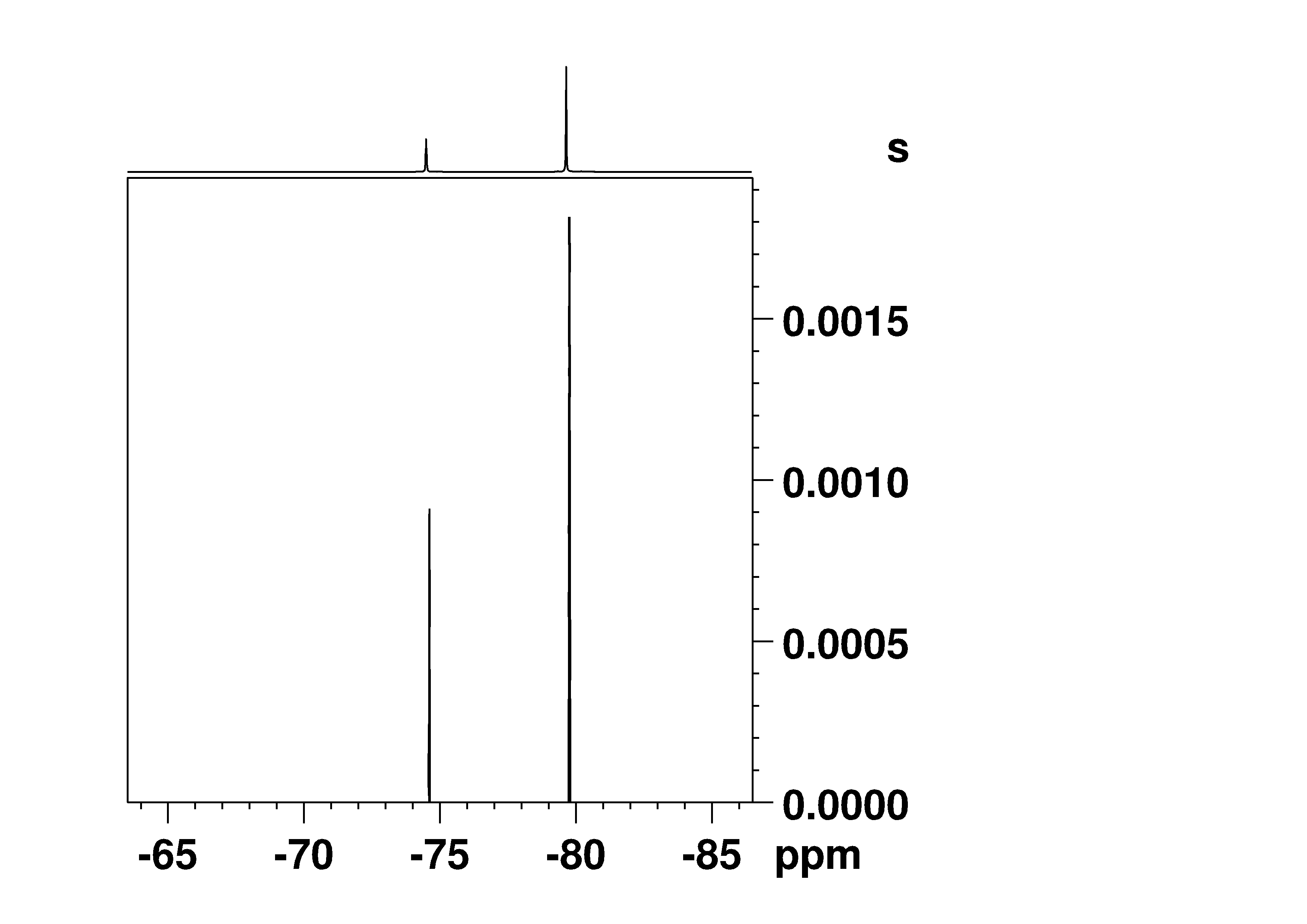


Figure S34. ^19^F DOSY NMR of [NPPA][TFSI]/Np_2_F at 298 K.


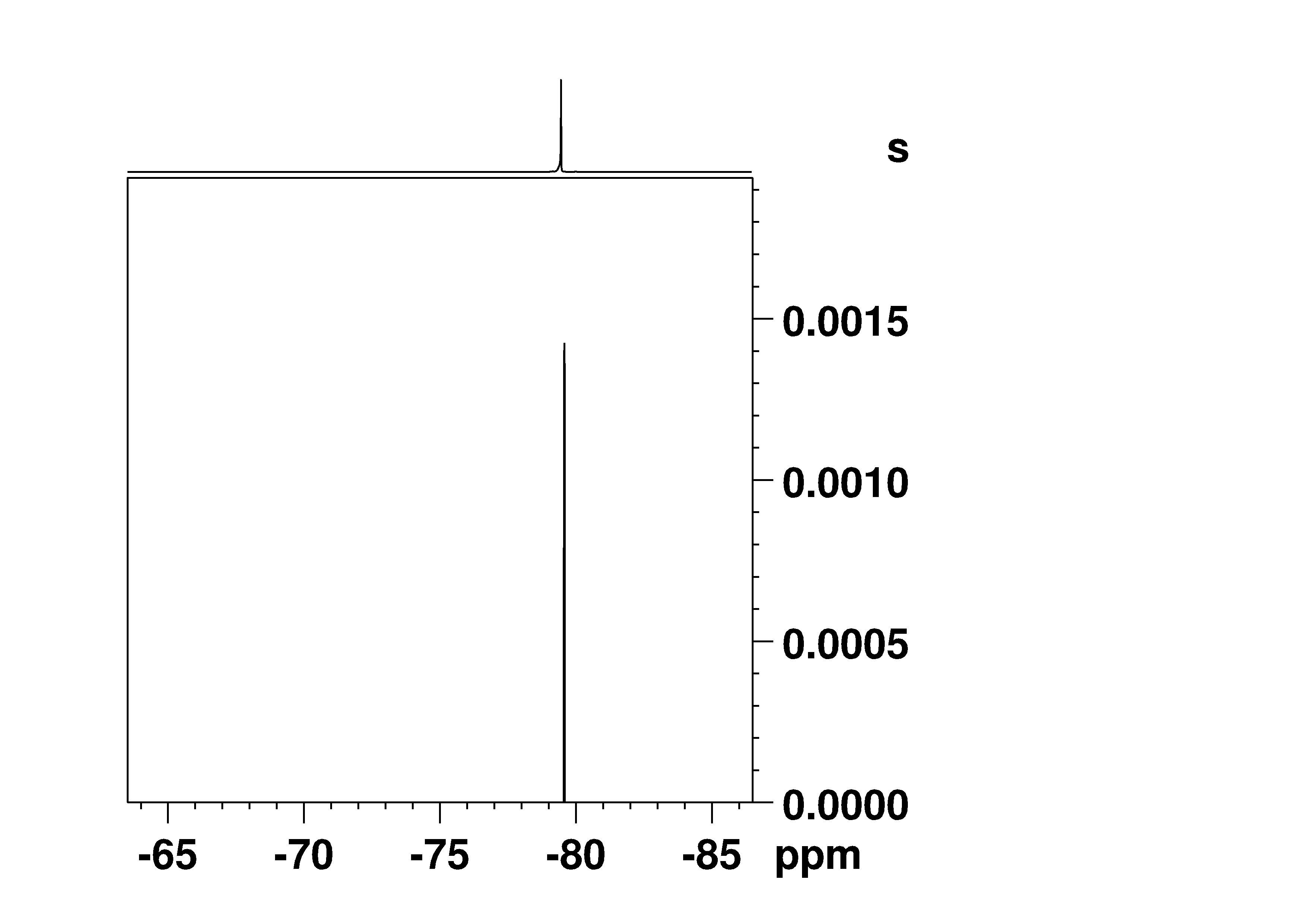


Figure S35. ^19^F DOSY NMR of [MNPA][TFSI] at 298 K.


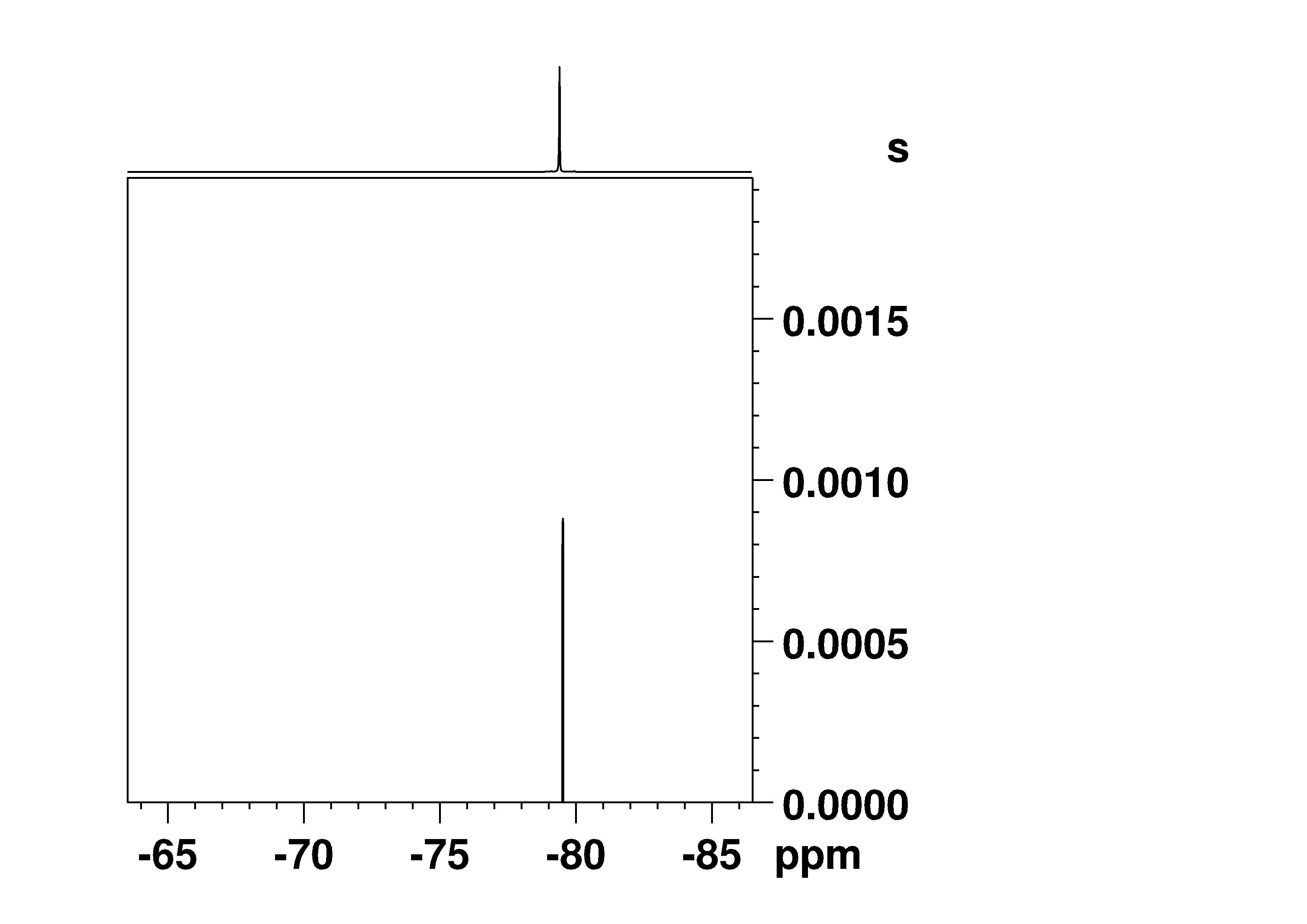


Figure S36. ^19^F DOSY NMR of [NPPA][TFSI]/Np_2_F at 298 K.


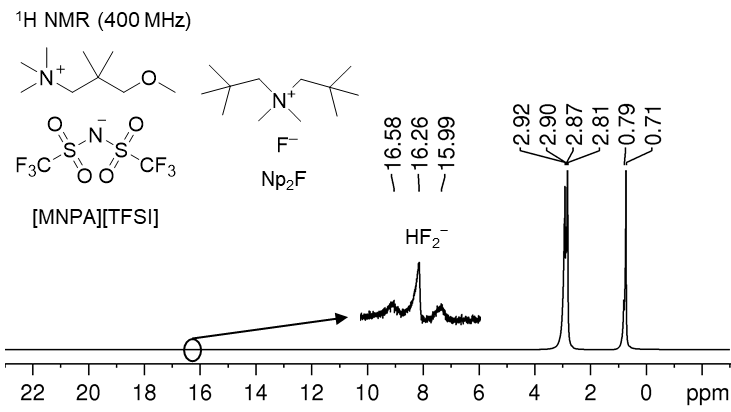


Figure S37. ^1^H NMR spectra for Np_2_F/[MNPA][TFSI].


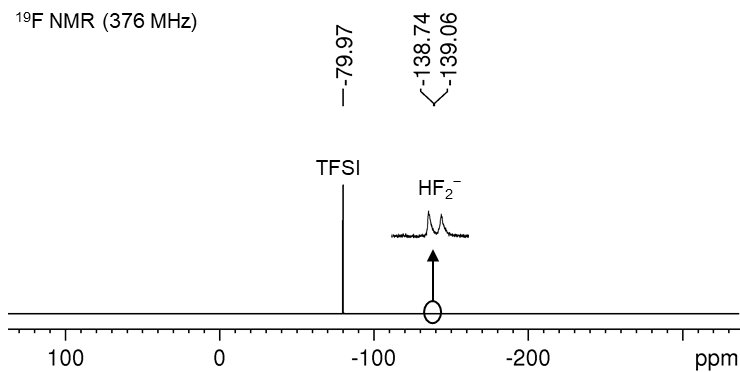


Figure S38. ^19^F NMR spectra for Np_2_F/[MNPA][TFSI].


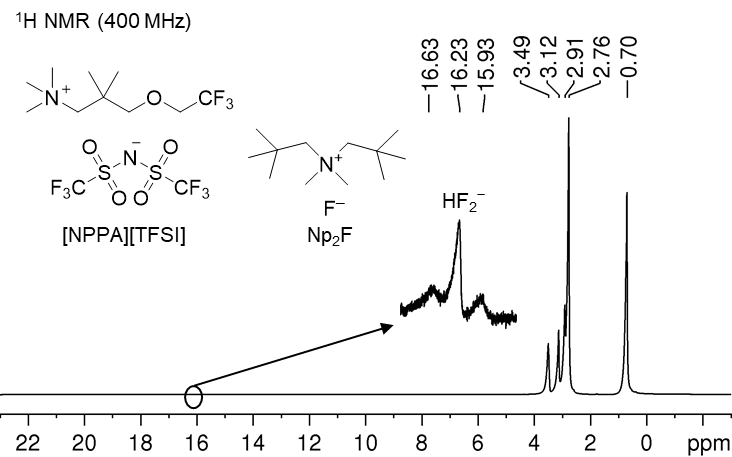


Figure S39. ^1^H NMR spectra for Np_2_F/[NPPA][TFSI].


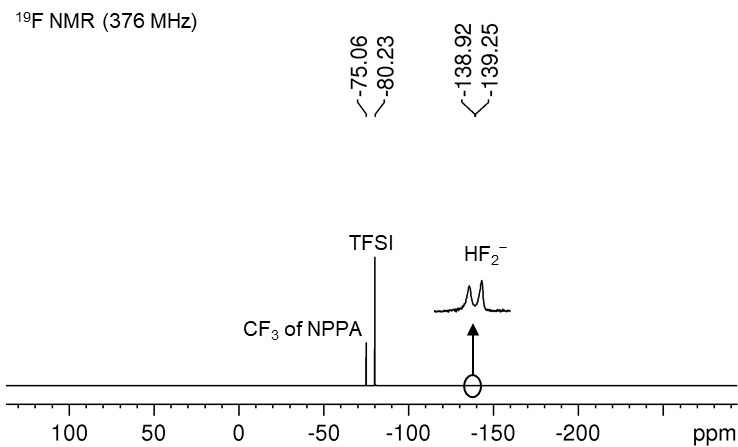


Figure S40. ^19^F NMR spectra for Np_2_F/[NPPA][TFSI].


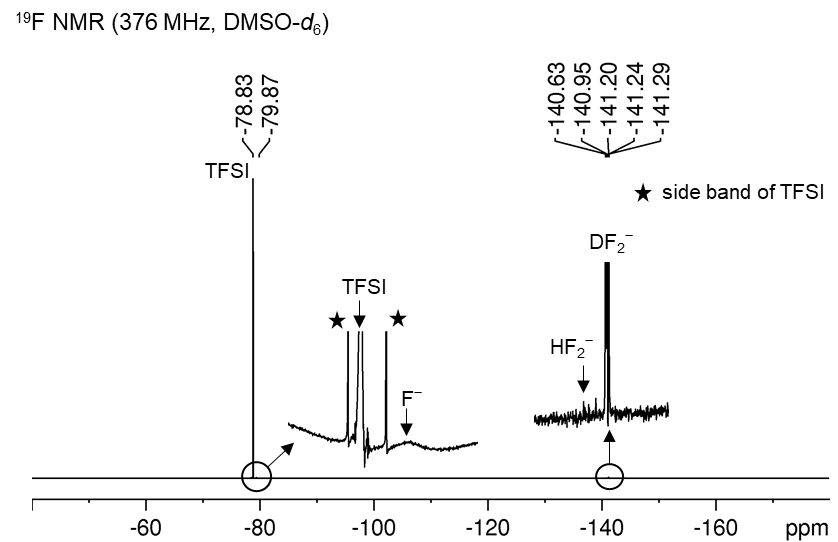


Figure S41. ^19^F NMR spectra for sampling Np_2_F/[MNPA][TFSI] in DMSO-*d*_6_.


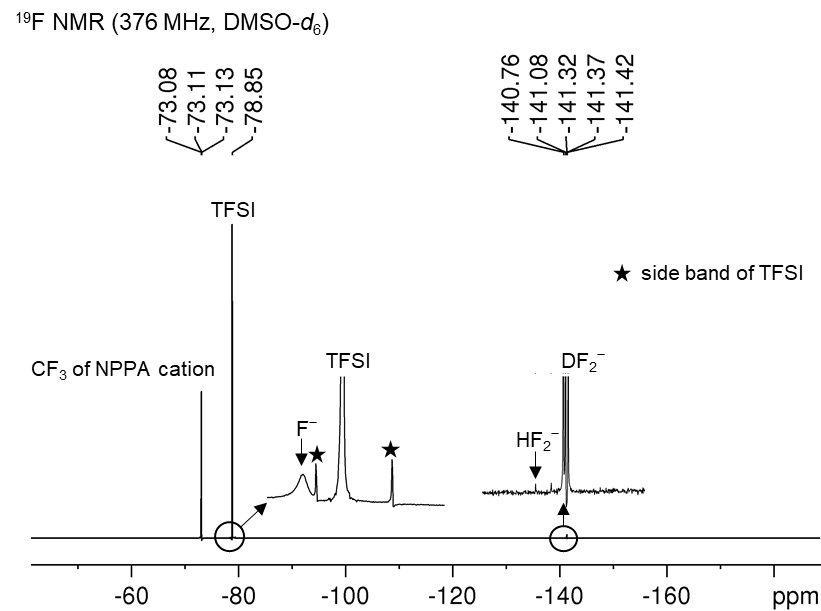


Figure S42. ^19^F NMR spectra for sampling Np_2_F/[MNPA][TFSI] in DMSO-*d*_6_.


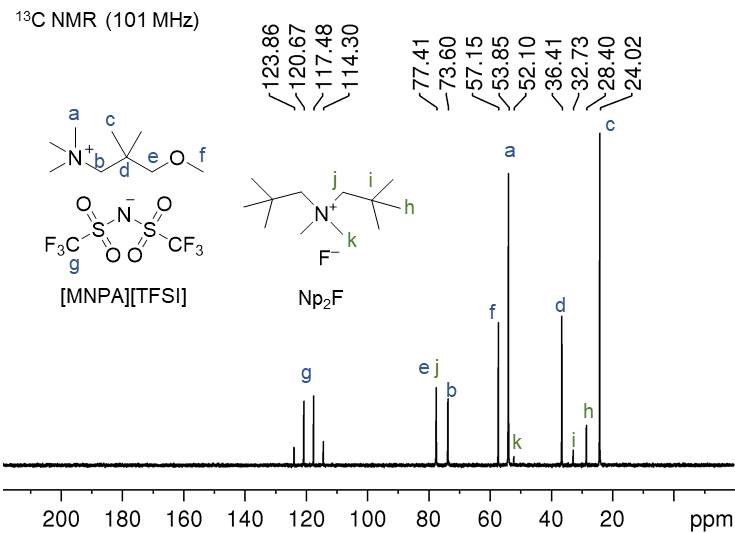


Figure S43. ^13^C NMR spectra for Np_2_F/[MNPA][TFSI].


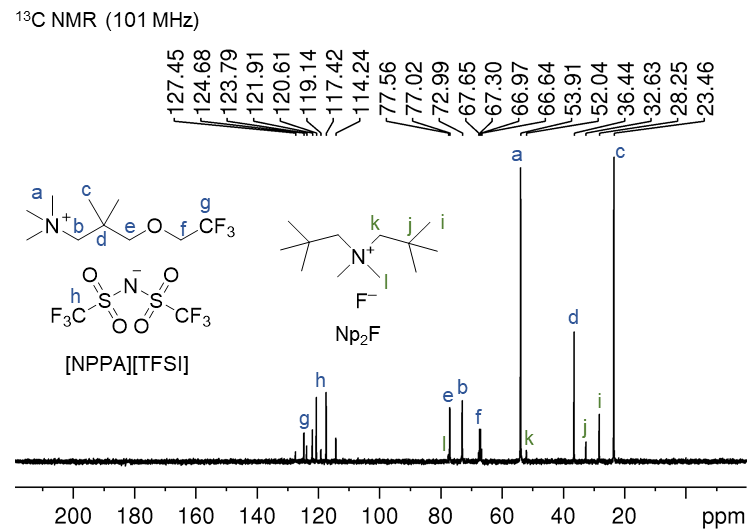


Figure S44. ^13^C NMR spectra for Np_2_F/[NPPA][TFSI].

## Battery Performance with 0.5 M Np_2_F/[NPPA][TFSI]


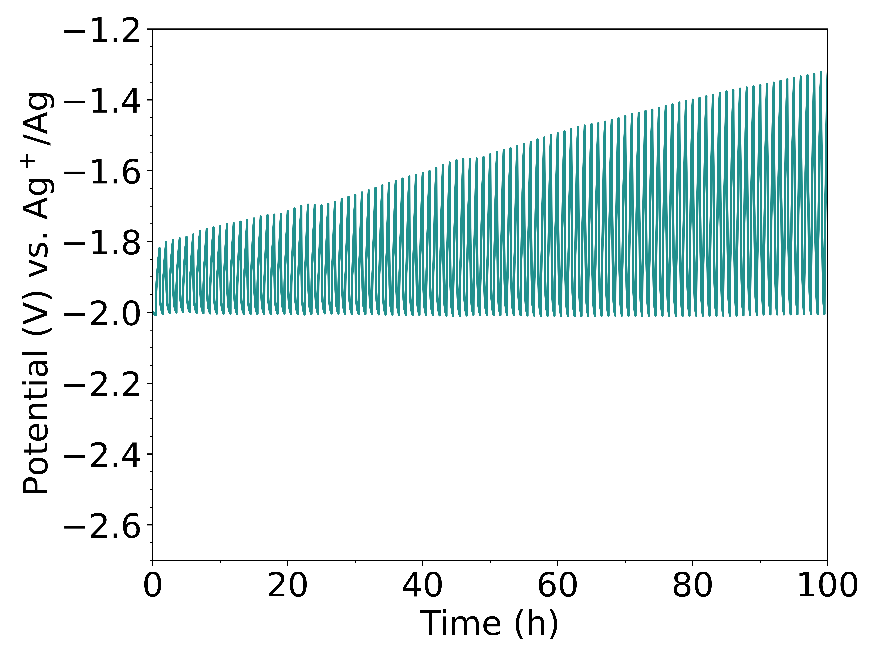


### Figure S45. Voltage profiles of the Pb/PbF_2_ electrode upon charging/discharging 1 μA/cm^2^ in 0.5 M Np_2_F/[NPPA][TFSI] electrolyte.

## Impedance Data


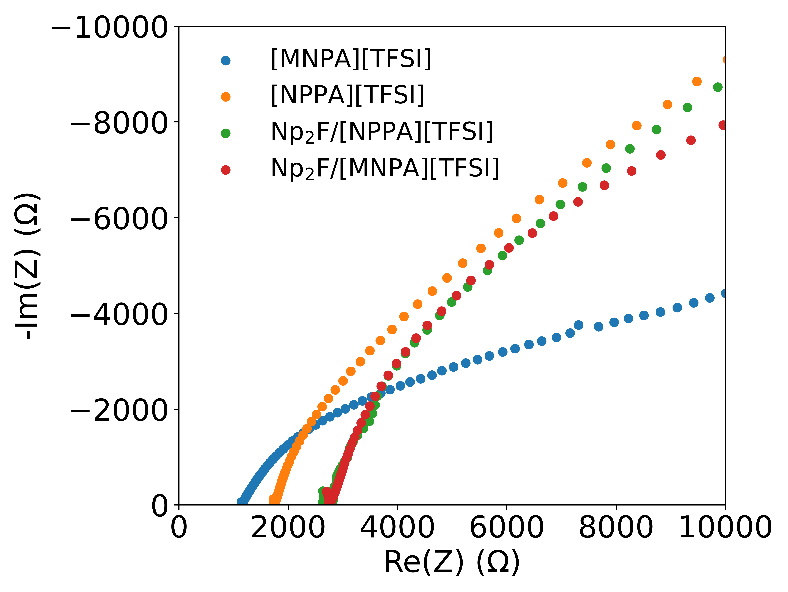


### Figure S46. Complex impedance data for the neat ionic liquids and ionic liquid electrolytes used in this work.

## Differential Scanning Calorimetry (DSC)


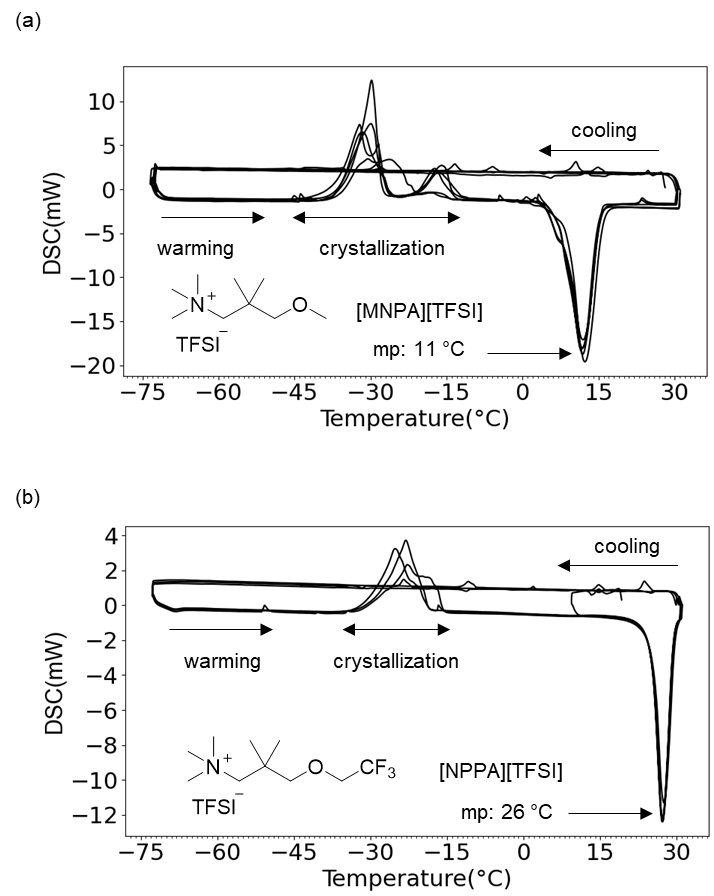


### Figure S47. DSC trace of (a) [MNPA][TFSI] and (b) [NPPA][TFSI] with multiple temperature cycles. The scan rate was 3 °C/min.

## Voltage Window


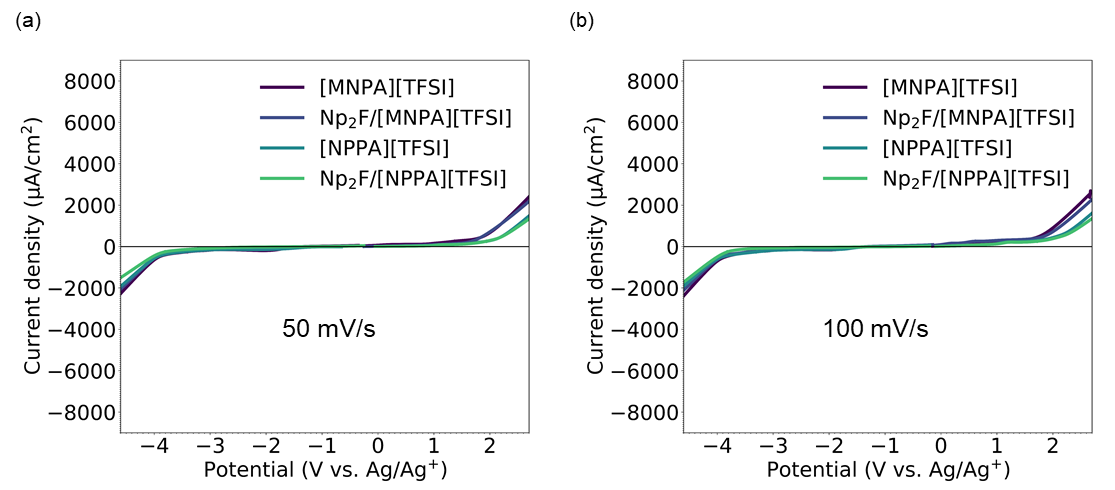


Figure S48. Linear sweep voltammetry for [MNPA][TFSI], 0.1 M Np_2_F/[MNPA][TFSI], [NPPA][TFSI], and 0.1 M Np_2_F/[NPPA][TFSI] measured at scan rates of (a) 50 mV/s and (b) 100 mV/s.

### Table S1. Voltage windows measured at different scan rates. The cutoff was set at 100 μA/cm^2^ for 1 mV/s, to 700 for 50 mV/s, and 1000 μA/cm^2^ for 100 mV/s. Potentials are referenced to Ag/Ag^+^.

| Electrolyte | Scan rate  (mV/s) | *E*_red_  (V) | *E*_ox_  (V) | *E*_window_  (V) |
| --- | --- | --- | --- | --- |
| [MNPA][TFSI] | 1 | –3.6 | 2.0 | 5.6 |
| [MNPA][TFSI] | 50 | –3.7 | 2.1 | 5.8 |
| [MNPA][TFSI] | 100 | –3.9 | 1.8 | 5.7 |
| [NPPA][TFSI] | 1 | –3.6 | 2.2 | 5.8 |
| [NPPA][TFSI] | 50 | –3.7 | 2.1 | 5.8 |
| [NPPA][TFSI] | 100 | –3.9 | 2.1 | 6.0 |
| Np_2_F/[MNPA][TFSI] | 1 | –3.6 | 1.8 | 5.4 |
| Np_2_F/[MNPA][TFSI] | 50 | –3.7 | 1.8 | 5.5 |
| Np_2_F/[MNPA][TFSI] | 100 | –3.9 | 1.9 | 5.8 |
| Np_2_F/[NPPA][TFSI] | 1 | –3.6 | 2.1 | 5.7 |
| Np_2_F/[NPPA][TFSI] | 50 | –3.9 | 2.1 | 6.0 |
| Np_2_F/[NPPA][TFSI] | 100 | –4.0 | 2.2 | 6.2 |


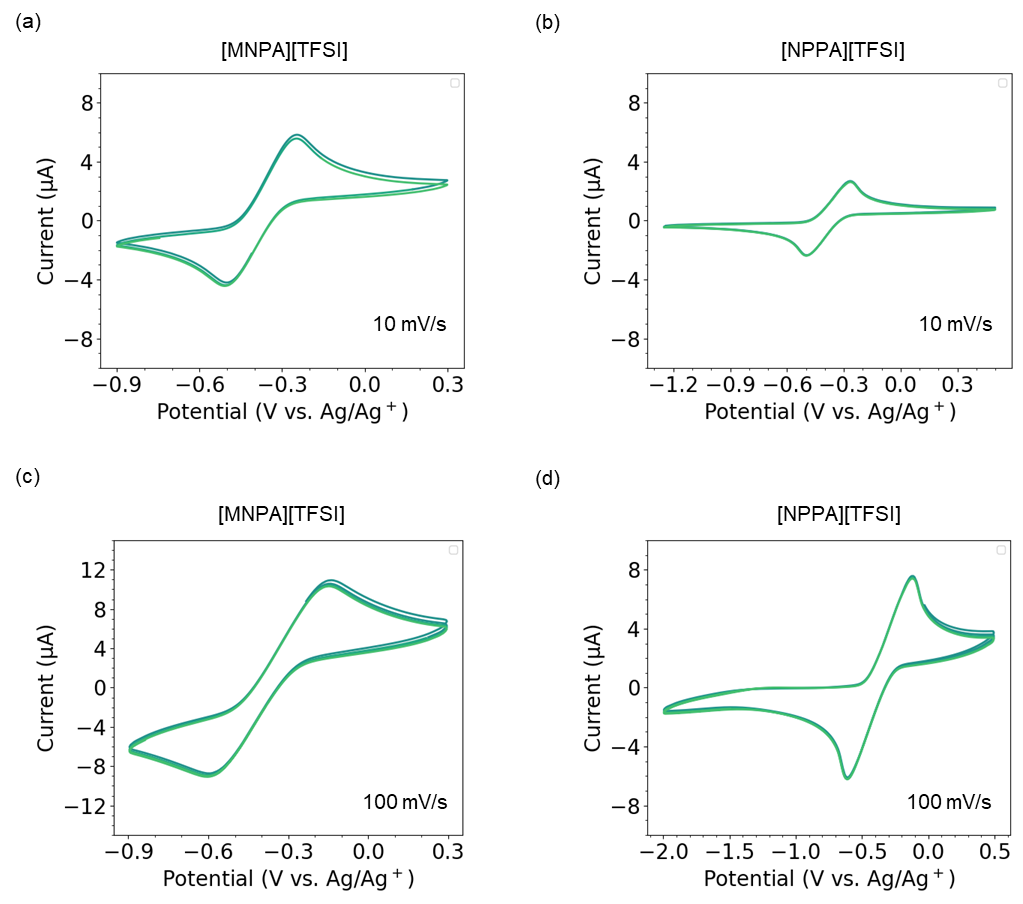


### Figure S49. Cyclic voltammograms of ferrocene in neat ionic liquids, (a) [MNPA][TFSI] with 10 mV/s scan rate, (b) [MNPA][TFSI] with 100 mV/s scan rate, (c) [NPPA][TFSI] with 10 mV/s scan rate, and (d) [NNPA][TFSI] with 10 mV/s scan rate.

### Table S2. The potential of ferrocene in neat ionic liquids. Potentials reported vs. Ag/Ag^+^.

| Electrolyte | Scan rate  (mV/s) | *E*_red_  (V) | *E*_ox_  (V) | *E*_1/2_  (V) |
| --- | --- | --- | --- | --- |
| [MNPA][TFSI] + Fc | 10 | –0.51 | –0.25 | –0.38 |
| [NPPA][TFSI] + Fc | 10 | –0.50 | –0.27 | –0.39 |
| [MNPA][TFSI] + Fc | 100 | –0.59 | –0.15 | –0.37 |
| [NPPA][TFSI] + Fc | 100 | –0.61 | –0.13 | –0.37 |

### Table S3. Voltage window, diffusivity, and conductivity values for various electrolytes with aprotic solvents. The values are reported for room temperature.

| Electrolyte | Voltage  Window (V) | Diffusivity  (m^2^s^–1^) | Conductivity  (mS/cm) | Ref. |
| --- | --- | --- | --- | --- |
| 1.1 M KF/18C6/PC | - | - | 0.64 | ^[15]^ |
| 1.1 M KHF_2_/18C6/PC | 3.7 | - | 1.7 | ^[15]^ |
| 0.45 M CsF-TPhBX-G4 | 2.4 | 6.28×10^−11^ | - | ^[16,17]^ |
| CsF(0.45)-FBTMPhB(0.5)-G4 | 1.9 | 7.63×10^−11^ | - | ^[17,18]^ |
| LiBOB_0.5_/CsF/G4 | 3.2 | - | 2.27 | ^[19]^ |
| Py/CsF/G4 | 2.6 | - | 2.7 × 10^−2^ | ^[20]^ |
| An/CsF/G4 | 3.4 | - | 3.2 × 10^−3^ | ^[20]^ |
| 0.5 M TSbFCl/DMSO | 2.5 | - | 0.74 | ^[21]^ |
| 0.7 M TMAF+TPFPB/DMSO | 1.7 | - | 6.1 | ^[22]^ |
| MPPF/TMPA-TFSA | 2.5 | - | 2.5 | ^[23,24]^ |
| 0.75 M Np_1_F/BTFE | 3.6 | 4.84×10^−10^ | 2.4 | ^[25]^ |
| 0.75 M Np_1_F/BTFE + G4 | 4.2 | - | - | ^[25]^ |
| 0.75 M Np_2_F/BTFE | - | 4.73×10^−10^ | 2.8 | ^[25]^ |
| 0.9 M anhydrous Np_2_F/BTFE | 3.5 | 2.68×10^−10^ | 2.8 | ^[1]^ |
| 0.9 M anhydrous MeDMBF/BTFE | 3.3 | 2.15×10^−10^ | 3.0 | ^[14]^ |
| 0.9 M anhydrous NpDMBF/BTFE | 3.5 | 2.87×10^−10^ | 4.0 | ^[14]^ |
| 0.5 M TMAF/[MMIm][TFSI] | 4.7 | 1.36×10^–11^ | 9.5 | ^[26]^ |
| 0.5 M Np_2_F/[MNPA][TFSI] | 5.4 | 3.44×10^−12^ | 0.8 | This work |
| 0.5 M Np_2_F/[NPPA][TFSI] | 5.7 | 2.41×10^−12^ | 0.8 | This work |


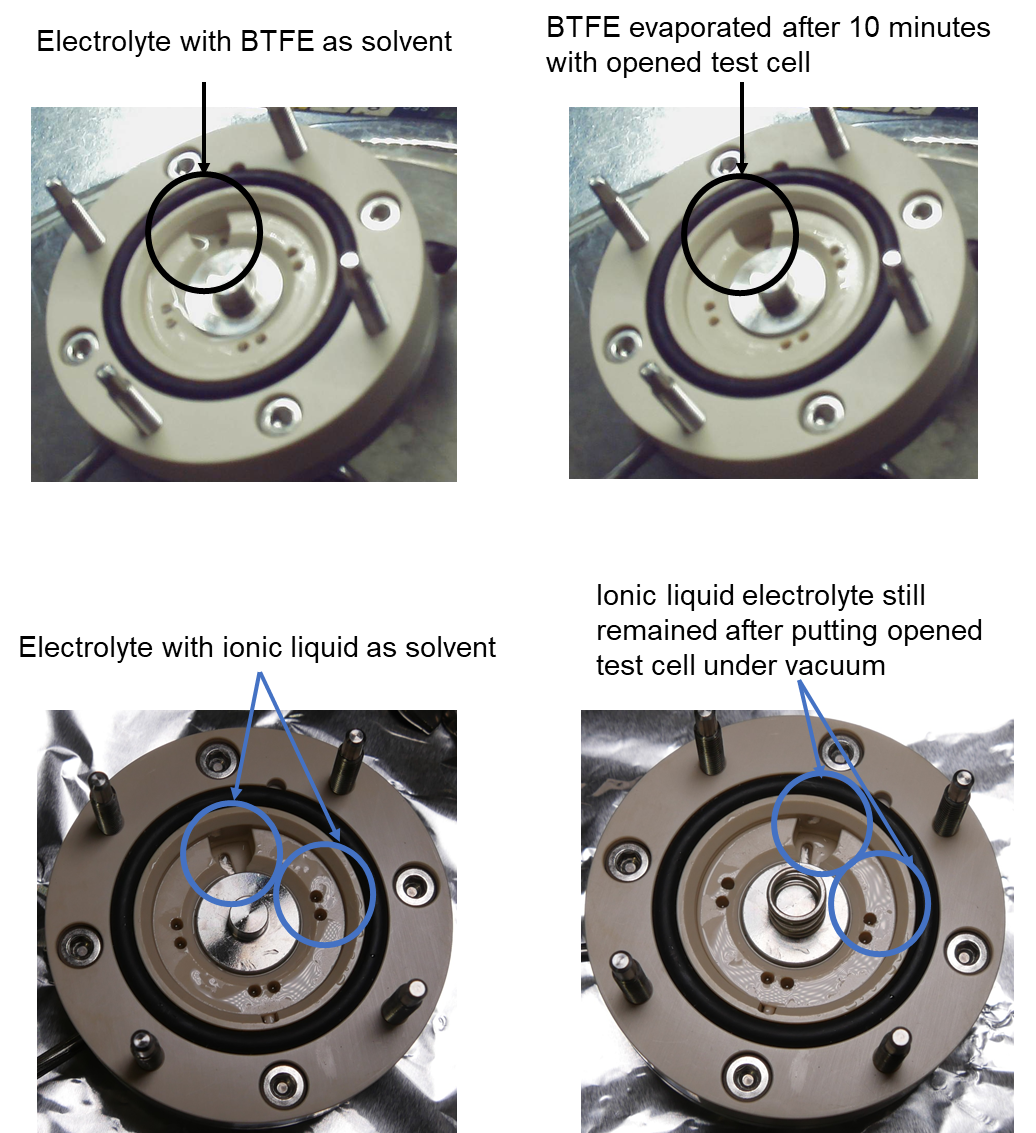


Figure S50. Photographs of the test cell.

## Battery Performance of PbF_2_ Working Electrode with 0.5 M Np_2_F/[MNPA][TFSI]


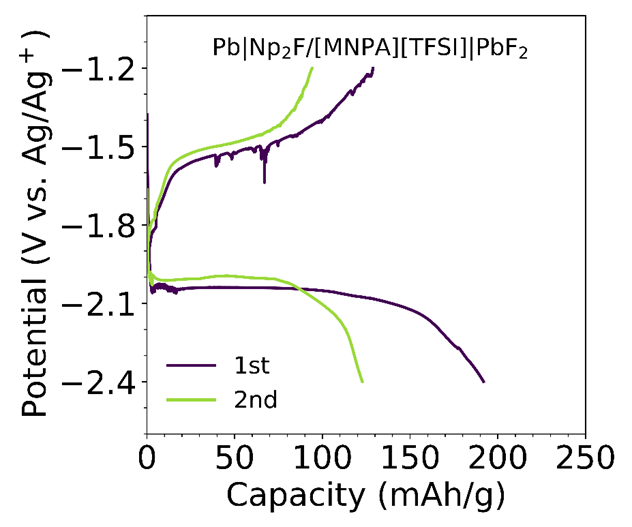


Figure S51. Charge/discharge curve (0.05 C) for the PbF_2_ electrode in 0.5 M Np_2_F/[MNPA][TFSI]. The loading mass of PbF_2_ was 0.4 mg. The initial discharge capacity was 192 mAh/g.

## Author Contributions

T.T. conceived the idea and designed the experiment. T.T. synthesized the materials and performed the measurements and characterizations. T.T., S.S. and T.S. fabricated the battery materials. S.S. and T.S. assisted with the electrochemical experiments. T.T., R.M., and A.W. prepared the original manuscript. The manuscript was revised by T.T., R.M., T.S., T.A., and A.W. The project was supervised by A.W.

## Reference

[1] T. Tan, R. Murdey, S. Sumitomo, T. Nakamura, M. A. Truong, A. Wakamiya, *Chem. Mater.* **2024**, *36*, 4553–4560.

[2] P. Zou, C. Wang, Y. He, H. L. Xin, R. Lin, *Nano Lett.* **2024**, *24*, 5429–5435.

[3] G. M. Sheldrick, *Acta Crystallogr. Sect. Found. Adv.* **2015**, *71*, 3–8.

[4] O. V. Dolomanov, L. J. Bourhis, R. J. Gildea, J. a. K. Howard, H. Puschmann, *J. Appl. Crystallogr.* **2009**, *42*, 339–341.

[5] G. M. Sheldrick, *Acta Crystallogr. Sect. C Struct. Chem.* **2015**, *71*, 3–8.

[6] F. Neese, *WIREs Comput. Mol. Sci.* **2022**, *12*, e1606.

[7] S. Grimme, S. Ehrlich, L. Goerigk, *J. Comput. Chem.* **2011**, *32*, 1456–1465.

[8] S. Grimme, J. Antony, S. Ehrlich, H. Krieg, *J. Chem. Phys.* **2010**, *132*, 154104.

[9] F. Weigend, R. Ahlrichs, *Phys. Chem. Chem. Phys.* **2005**, *7*, 3297–3305.

[10] F. Weigend, *Phys. Chem. Chem. Phys.* **2006**, *8*, 1057–1065.

[11] M. D. Hanwell, D. E. Curtis, D. C. Lonie, T. Vandermeersch, E. Zurek, G. R. Hutchison, *J. Cheminformatics* **2012**, *4*, 17.

[12] G. Knizia, J. E. M. N. Klein, *Angew. Chem. Int. Ed.* **2015**, *54*, 5518–5522.

[13] G. Knizia, *J. Chem. Theory Comput.* **2013**, *9*, 4834–4843.

[14] T. Tan, R. Murdey, S. Sumitomo, A. Wakamiya, *Sustain. Energy Fuels* **2025**, DOI 10.1039/D4SE01432D.

[15] S. Kawauchi, H. Nakamoto, R. Takekawa, T. Kobayashi, T. Abe, *ACS Appl. Energy Mater.* **2022**, *5*, 2096–2103.

[16] H. Konishi, T. Minato, T. Abe, Z. Ogumi, *Chem. Lett.* **2018**, *47*, 1346–1349.

[17] H. Konishi, R. Takekawa, T. Minato, Z. Ogumi, T. Abe, *Chem. Phys. Lett.* **2020**, *755*, 137785.

[18] H. Konishi, T. Minato, T. Abe, Z. Ogumi, *J. Electrochem. Soc.* **2017**, *164*, A3702–A3708.

[19] A. C. Kucuk, T. Minato, T. Yamanaka, T. Abe, *J. Mater. Chem. A* **2019**, *7*, 8559–8567.

[20] A. Celik Kucuk, T. Abe, *J. Fluor. Chem.* **2020**, *240*, 109672.

[21] D. Li, G. Li, Y. Yu, C. Li, *Adv. Mater.* **n.d.**, *n/a*, 2415106.

[22] Z. Fu, X. Yang, Y. Tian, X. Hu, Y. Wang, L. Lin, F. Kang, G. Wang, B. Li, D. Zhou, *Energy Storage Mater.* **2024**, *70*, 103533.

[23] K. Okazaki, Y. Uchimoto, T. Abe, Z. Ogumi, *ACS Energy Lett.* **2017**, *2*, 1460–1464.

[24] K. Okazaki, H. Nakamoto, T. Yamanaka, T. Fukunaga, Z. Ogumi, T. Abe, *Chem. Mater.* **2022**, *34*, 8280–8288.

[25] V. K. Davis, C. M. Bates, K. Omichi, B. M. Savoie, N. Momčilović, Q. Xu, W. J. Wolf, M. A. Webb, K. J. Billings, N. H. Chou, S. Alayoglu, R. K. McKenney, I. M. Darolles, N. G. Nair, A. Hightower, D. Rosenberg, M. Ahmed, C. J. Brooks, T. F. Miller, R. H. Grubbs, S. C. Jones, *Science* **2018**, *362*, 1144–1148.

[26] O. Alshangiti, G. Galatolo, C. Di Mino, T. F. Headen, J. Christianson, S. Merotto, G. J. Rees, Y. Delavoux, M. Swadźba-Kwaśny, M. Pasta, *ACS Energy Lett.* **2024**, *9*, 6104–6108.
